# The transmission game: Testing behavioral interventions in a pandemic-like simulation

**DOI:** 10.1126/sciadv.abk0428

**Published:** 2022-02-25

**Authors:** Jan K. Woike, Sebastian Hafenbrädl, Patricia Kanngiesser, Ralph Hertwig

**Affiliations:** 1 Center for Adaptive Rationality, Max Planck Institute for Human Development, Lentzeallee 94, 14195 Berlin, Germany.; 2 School of Psychology, University of Plymouth, Portland Square, Drake Circus, Plymouth PL4 8AA, UK.; 3 IESE Business School, Avenida Pearson, 21, 08034 Barcelona, Spain.

## Abstract

During pandemics, effective nonpharmaceutical interventions encourage people to adjust their behavior in fast-changing environments in which exponential dynamics aggravate the conflict between the individual benefits of risk-taking and its social costs. Policy-makers need to know which interventions are most likely to promote socially advantageous behaviors. We designed a tool for initial evaluations of the effectiveness of large-scale interventions, the transmission game framework, which integrates simulations of outbreak dynamics into large-group experiments with monetary stakes. In two studies (*n* = 700), we found substantial differences in the effectiveness of five behavioral interventions. A simple injunctive-norms message proved most effective, followed by two interventions boosting participants’ ability to anticipate the consequences of risky behavior. Interventions featuring descriptive norms or concurrent risk information failed to reduce risk-taking.

## Teaser

The effectiveness of five behavioral interventions to reduce risk-taking in a simulated outbreak varied substantially.

## INTRODUCTION

Government responses to the COVID-19 pandemic have relied heavily on nonpharmaceutical interventions (*1*–*4*). These interventions have been informed by epidemiological and econometric modeling that either simulates future impact or retrospectively evaluates effectiveness (*5*–*10*). Yet, population-wide models have often relied on mechanistic assumptions about human behavior in a novel and highly uncertain environment without grounding these assumptions in a solid empirical foundation. This is problematic, as the key nonpharmaceutical interventions—such as wearing masks, maintaining physical distance, and reducing contacts—require large-scale behavior change, which depends on individual compliance and cooperation. The behavioral sciences offer cognitive tools to foster behavioral change and cooperation (*11*–*13*). The effectiveness of these instruments to increase compliance with nonpharmaceutical interventions, however, has rarely been tested experimentally in circumstances reflecting the dynamics of infectious outbreaks. These circumstances produce nonlinear and fast-changing environments that are replete with conflicts between individual self-interest and the common good (e.g., if enough individuals reduce their social contacts and wear masks, others can free-ride and enjoy protection without altering their own behavior).

We propose a versatile approach that combines the utility of nonlinear simulations to map transmission dynamics with that of experimental investigations of human decision-making. In response to the COVID-19 pandemic, behavioral scientists have mostly conducted survey and scenario studies (see section E2). To overcome concerns about intention-behavior gaps and distorting effects due to social desirability and cheap talk, we build on existing research that has extended standard incentivized economic games, morphing public good games into vaccination games or adding risk components to dictator games (*14*–*17*). Experimental game paradigms address causal mechanisms (*18*) and offer the advantage of fast, cost-efficient, and ethical tests of scalable behavioral interventions (*19*). Specifically, we present and investigate a novel framework that simulates a progressive transmission process in a population and measures degrees of incentivized risk-taking in this evolving outbreak.

Our design aimed to map three key elements of the pandemic onto a game (see section S2): (i) nonlinear spread dynamics, (ii) the experience of a social dilemma (individually advantageous actions heighten collective risk), and (iii) the compounding of small transmission risks. Our framework makes it possible to test the effectiveness of a wide variety of behavioral interventions. It can be flexibly implemented with various group sizes, in asynchronous and synchronous settings, both offline and online, and is communicable to broad parts of the population.

The game, in its basic form, is thematically neutral and avoids epidemiological terminology to afford some distance to the omnipresent COVID-19 pandemic. We chose this approach for both methodological and ethical reasons. Avoiding pandemic-related terminology in describing the game ensures replicability of the study irrespective of the state of the pandemic and people’s respective beliefs and political attitudes (*20*, *21*). Moreover, neutrally framed interventions do not copy or compete with interventions already implemented outside the laboratory. A neutral framing also reduces the risk of adverse effects. For example, if an intervention in a neutral scenario led to an increase in undesired behaviors, then it would not affect participants’ behavior in the real world. Such negative effects on real-world intentions have been observed in studies with thick framing (*22*, *23*).

**Fig. 1.**
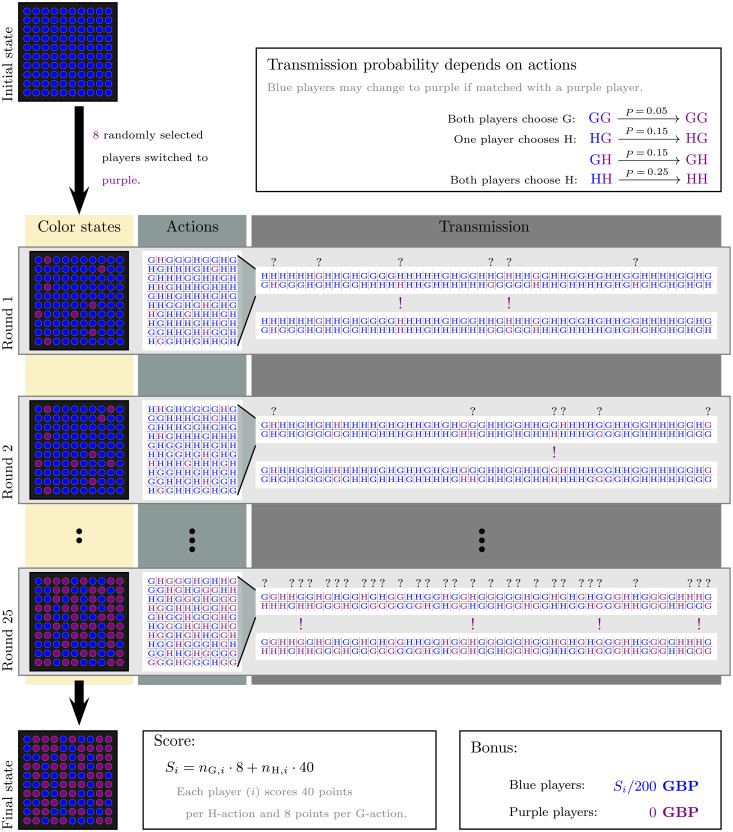
Transmission game rules. The game flow is shown from top to bottom (with hypothetical choices and round outcomes). All 100 players start as blue; then, 8 randomly selected players are switched to purple (infected; this represents the initial outbreak). In each of 25 rounds, players decide between two actions: action G offering low risk and low reward (8 points), and action H offering high risk and high reward (40 points). All players are randomly paired. Blue paired with purple players may switch to purple; the transmission probability is between 0.05 and 0.25 and is determined by the pair’s chosen actions (with a cumulative increase of 0.1 per choice of action H). Last, scores summed across all rounds are translated into payoffs for blue players only, at a rate of 1 GBP per 200 points.

In our game, participants were initially assigned the color blue (as a thematically neutral representation of being healthy) and were informed that any monetary payoff was contingent on them not changing from blue to purple (corresponding to being infected) throughout the game. Switches to purple were possible either at the start of the game through random alterations of color (early outbreak) or in the course of the game: Participants were randomly paired in each of 25 rounds. A blue player paired with a purple (infected) player faced a risk of color switch (virus transmission). In each round, and without knowing their color or the color of their partner, participants chose between two actions, G or H. Action G was associated with a low payoff and a low risk of switching to purple. Action H offered a higher payoff but at the expense of a higher risk of switching. In our studies, groups of 100 participants played the game online and asynchronously. The rules of the game are summarized in Fig. 1.

At the time of the study (May 2020), little was known about the exact risks associated with different transmission pathways or the precise effect of countermeasures. We thus chose parameters and elements that captured established qualitative aspects of the pandemic. The two actions (G and H) represented different degrees of embracing preventive measures such as wearing face masks and physical distancing (with G being the more careful choice). These measures were modeled to protect both the player choosing them and the player they were paired with, in the same way as face masks protect both the wearer and those in contact with them. The degree of protection was not complete, and a small chance of infection remained even when both participants chose action G (*P* = 0.05). At the same time, infection was clearly the less likely outcome, even when one (*P* = 0.15) or both participants (*P* = 0.25) chose action H. The different payoffs for the two actions represented opportunity costs and other costs of prevention; the loss of all payoff after changing to purple corresponded to the costs of infection.

**Fig. 2.**
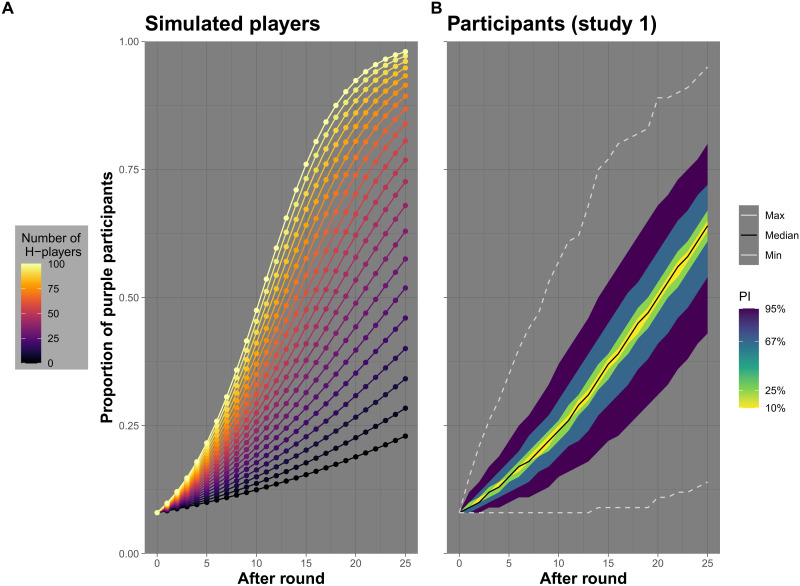
Simulated game results. (**A**) Average proportion of purple players at the beginning and after each of the 25 rounds for games in which a fixed number of players choose action H in all rounds (and the remainder choose action G in all rounds). Colors correspond to different numbers of players selecting action H. (**B**) Distributions of the proportion of purple players after each round in simulated games based on participants’ responses in study 1. The middle line corresponds to the median across 1,000,000 simulations; shaded areas show prediction intervals containing the inner 10%, 25%, 67%, and 95% of results.

Before experimentation, we conducted a series of simulations to map the relationship between player behavior and game outcomes. Figure 2A shows the average progression of color-switching rates in populations with different proportions of simulated players choosing the safe or the risky action across all rounds (see section S3 for details and additional results). On average, between 23.0% and 98.0% of players turn purple before the end of the game, and thus leave empty-handed. An analysis of individual expected payoffs demonstrates that the game constitutes a social dilemma: No matter how many participants choose the safe action G, the expected payoff for choosing the risky action H is higher. At the same time, the expected payoff for safe players decreases with every choice of the risky action. The average population payoff decreases with the percentage of risky choices across the population (once they exceed 10% of all choices). Along with this social dilemma, the game also entails a property of many resource-scarce environments, a risk-reward tradeoff (*24*): Higher expected payoffs are, all other things being equal, associated with a higher risk of ending the game empty-handed.

On the basis of these simulations, we conducted two preregistered empirical studies with U.S. participants (see section S1). Study 1 (*n*_1_ = 100) evaluated the framework by testing the variability of decisions without intervention. It also produced the data for some of the behavioral interventions tested in study 2. Study 2 (*n*_2_ = 600) investigated the effectiveness of five behavioral interventions (between subjects) in reducing the choice of the risky action relative to a control condition (see also Table 1). We chose these interventions because they resembled nonpharmaceutical interventions commonly used throughout the early pandemic to encourage safe behaviors. Some of our interventions focused on explaining transmission dynamics. Others used data from study 1 to provide information about the number of color switches or choice frequencies—similar to information that has been available throughout the pandemic (case rates and observations of others’ behavior). We describe each of the interventions in turn (see section S7 for screenshots of all conditions, and sections E9 to E12 for further details about the interventions).

**Table 1.** List of interventions in study 2.

**Intervention**	**Short name**	**Summary**	**Timing**
Learning from others’ outcomes	LFO	In each round of the game, a line graph shows the number of purple participants in study 1 from the start to the round corresponding to the current round.	During the game
Observing behavior	OBS	In each round of the game, participants see the number of participants who chose action G in the corresponding round of study 1.	During the game
Messaging	MES	After an initial explanation, participants see a text message in each round: “Choose action G to protect your and other players’ bonus money.”	Before and during the game
Simulator	SIM	Participants see the number of purple participants in a line graph across rounds in repeatedly simulated scenarios.	Before the game
Chain of infection	COI	Participants see a demonstration of the potential cumulative effects of early infections in later rounds.	Before the game

In the learning from others’ outcomes intervention (*25*), participants could learn about the consequences of other players’ decisions by observing the outcomes of a group of previous participants (respondents in study 1). Throughout the health crisis, nations have had the opportunity to observe each other’s performance to learn how best to respond to the virus (*26*). Graphs of rising case numbers have featured prominently in news coverage and offered decision-makers insights into the dynamics of infections and changes in risk (*27*). In our intervention, participants observed the number of purple players in a previous game (study 1). Specifically, they were shown the number of purple participants from the start of that game to the round corresponding to the round they were currently playing in study 2. We chose a simulated game with an outcome close to the expected outcome, and we presented the stepwise increase in purple players across rounds in a line graph accompanied by a sentence describing the state of the game. The numbers for each datapoint were displayed in tooltip fields. Although we did not disclose which decisions resulted in an increase in purple players, it was evident that only risky actions (H) could cause such an increase. A participant who underestimated the consequences of risky actions could adjust their expectations based on these numbers.

We also tested two norm interventions (*28*–*30*). The first, the observing behavior intervention, focused on the behavior shown by other participants. Information on what others typically do (the descriptive norm) has been found to be an agent of change, although not always in the intended direction (*29*). For example, information about how many others wear a mask or have reduced their physical contacts can positively affect the behavior of individuals who want to conform with the majority. At the same time, it may justify copying minority behavior when conformity is far from universal and thus have a negative effect. In our study, participants in this intervention condition read a sentence about how many participants had chosen the low-risk action (action G) in the corresponding round of study 1.

The second norm intervention, the messaging intervention, focused on behavior that others typically approve or disapprove of (i.e., injunctive norms). Going beyond simple instructions, this intervention establishes morally relevant reasons for choosing careful over riskier behavior. Government messages to the public often invoke these types of norms. For example, early in the pandemic, the U.K. government used the slogan “Stay Home—Protect the NHS—Save Lives” to directly connect the decisions available to the decision-maker (i.e., staying home or going out) to beneficial outcomes with an increasingly moral significance. In contrast, the decision not to stay home [and therefore not to protect the National Health Service (NHS), and not to help save lives] appears morally inappropriate. In our study, we presented participants with an explanation about the potentially detrimental consequences of choosing the action with the higher payoff (action H), namely, the increased probability of turning purple and losing the bonus payment for both oneself and others. At the same time, we highlighted that the lower risk action (G) avoided such harms. In addition, we displayed a single-sentence summary (“Choose action G to protect your and other players’ bonus money”) prominently in each round.

Last, we included two interventions aimed at boosting participants’ ability to make informed decisions (*31*, *32*). These interventions targeted possible sources of misunderstanding in the game situation so that decision-makers were empowered to align their choices with their preferences and to make good, autonomous decisions. Specifically, we aimed at improving participants’ ability to grasp the exponential dynamics of transmission. A recent study found that participants implicitly assumed linear increases in infection numbers (*33*). This could lead to them underestimating future prevalence based on available data, which would likely bias risk estimates downward and result in objectively riskier choices than they would make given accurate beliefs.

The simulator intervention provided a game simulator tool that permitted participants to repeatedly sample possible outcomes and offered insights into the game dynamics for different levels of risk-taking across players (*34*). Simulations featured heavily in the early scientific response to the COVID-19 epidemic; for example, they were used in newspaper articles to demonstrate different outbreak dynamics (*35*) or the consequences of restrictions such as travel bans (*36*). In our intervention, participants witnessed five scenarios that differed in the proportion of simulated H-choices per round (1%, 25%, 50%, 75%, and 99%; the remainder were G choices). Participants selected a scenario via a button click that started a simulation run. A line graph illustrated the progression of the number of purple players across rounds, and a sentence summarized the final state. To encourage participants to experience the variance between and within simulation scenarios, each of the five scenarios had to be run at least three times before participants could proceed to the game (with no upper limit for exploration).

The chain of infection intervention demonstrated how early risk-taking and color switches could cascade down to later rounds and result in transmission chains affecting many players over time. This intervention was inspired by newspaper articles using simple visual demonstrations of nonlinear transmission, such as vertical binary tree diagrams (*37*) or people arranged in concentric circles with infections spreading outward from smaller to larger circles (*35*). Before the game, participants were shown a multilevel tree that expanded in several steps and explained the far-reaching consequences of early infection over the rounds of the game.

Table 1 summarizes the five interventions. One hundred participants were assigned to each intervention; an additional 100 participants in a control condition played the game without intervention. A detailed description of interventions with screenshots and simulation code is provided in the Supplementary Materials.

## RESULTS

### Games without intervention in study 1 and study 2

Study 1 provided a proof of concept: Game behavior varied, and the risky action was chosen in 35.6% of all choices across rounds and participants, resulting in an average simulated percentage of 63.2% purple players across 1,000,000 simulations of the game (see Fig. 2). The control condition of study 2 replicated this pattern, with 41.8% risky choices (and 69.1% of players ending the game as purple).

### Interventions in study 2

**Fig. 3.**
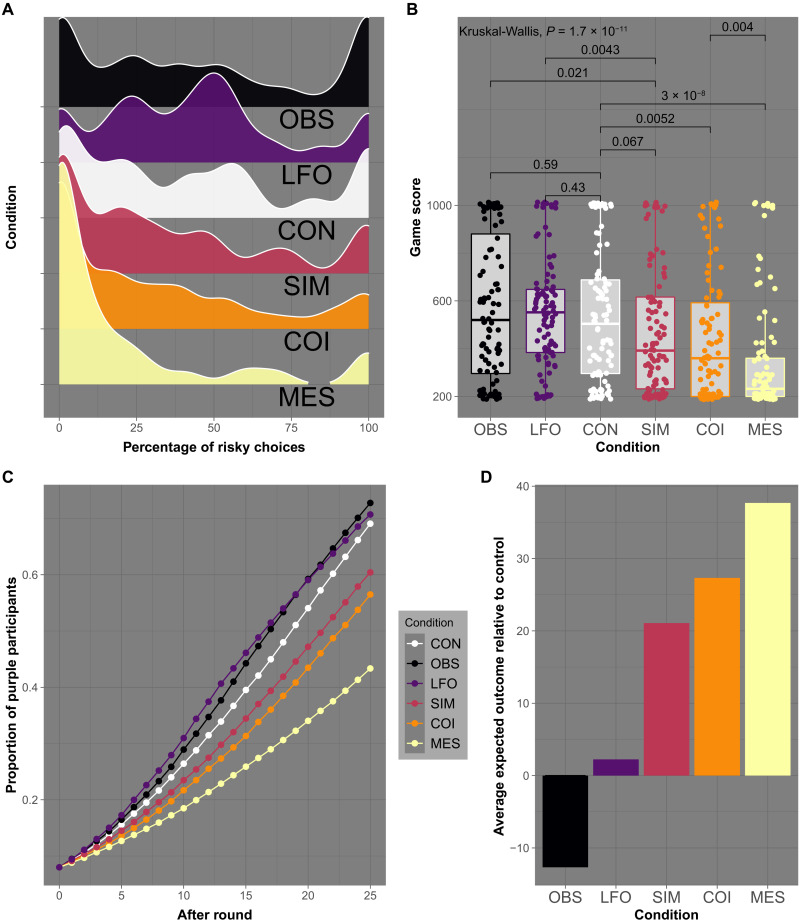
Results of study 2. (**A**) Distribution of the percentage of choices of the risky action (H) per participant by condition (ordered by descending means from top to bottom; OBS, observing behavior; LFO, learning from others’ outcomes; CON, control; SIM, simulator; COI, chain of infection; MES, messaging). (**B**) Boxplots (showing quartiles) of points scored after 25 rounds by condition, with dots representing individual scores. Numbers above brackets are *P* values for Mann-Whitney *U* tests comparing condition pairs. (**C**) Average proportion of purple (“infected”) participants after each round across 1,000,000 simulated games per condition. (**D**) Average expected outcomes per condition relative to the control condition (*M* = 141.59) with purple players scored as 0.

We found notable differences in effectiveness across the five intervention conditions (see Fig. 3, A and B). We report the findings in order of ascending effectiveness and relative to the control condition. In two of the five intervention conditions, the proportion of choices of the risky action was even higher than in the control condition: 45.8% risky choices in the observing behavior condition, resulting in 72.8% purple players, and 44% risky choices in the learning from others’ outcomes condition, resulting in 70.7% purple players (see Fig. 3C). The other three conditions—messaging, simulator, and chain of infection—led to fewer choices of the risky action and a lower percentage of purple players relative to the control condition. Following the two boosting interventions (simulator and chain of infection), participants chose the risky action in 33.6% (60.4% purple) and 30.0% (56.5% purple) of cases, respectively. The largest reduction was observed in the messaging condition with 18.6% risky choices, resulting in only 43.3% purple players. The average expected payoff reached a maximum in this condition (see Fig. 3D).

### Predictors of game behavior in study 2

Across participants in study 2, the number of individual choices of action H (high risk, high reward) was positively related to individuals’ general risk attitude (*38*) and negatively related to social value orientation (*39*). This is consistent with action H increasing the probability of ending the game empty-handed while at the same time increasing personal expected payoffs at the cost of other players’ payoffs.

We explored these relationships further after classifying the pattern of actions chosen across rounds into four categories: A group of 148 participants chose the safe option G in every single round (all safe, 24.7%). A group of 73 participants chose the risky option H in every single round (all risky, 12.2%). A group of 93 participants started the game with risky choices in at least one round and then chose the safe option for the rest of the game. Thus, their sequence of decisions showed a single change from consistently risky choices to consistently safe choices (risky-safe, 15.5%). As the number of purple players can only grow over time, higher transmission probabilities become more dangerous in later rounds. We demonstrate justifications for this decision pattern and the resulting flattening-of-the-curve effect in simulation studies in sections S3 and S4. The other 286 participants showed no clear decision patterns that could be meaningfully distinguished. All of them switched more than once between actions, and we categorized them into a single category (switch, 47.7%).

**Fig. 4.**
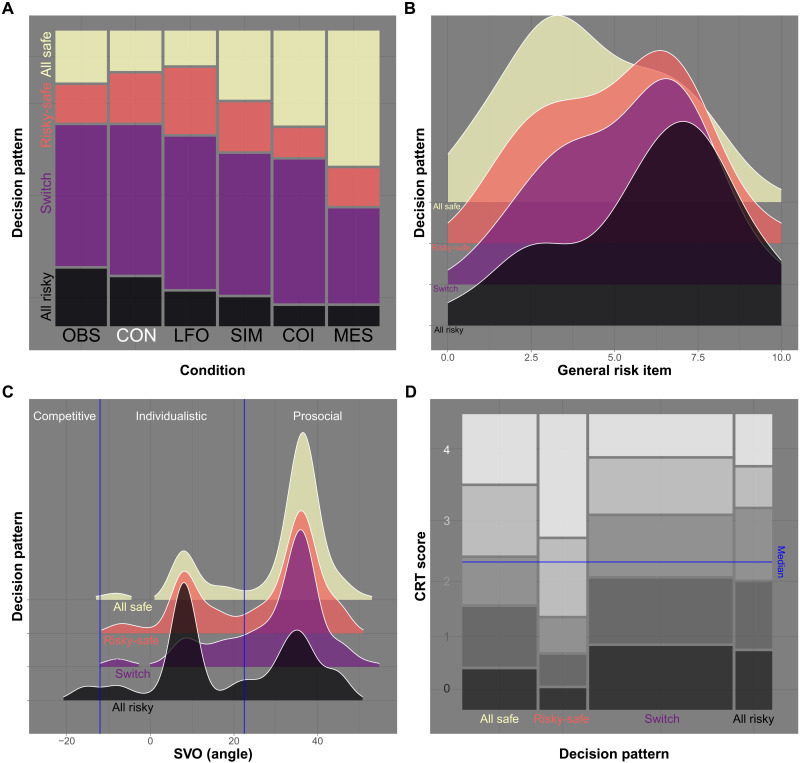
Decision patterns and predictors. (**A**) Mosaic plot showing the relative frequency of the four decision patterns across the six conditions (OBS, CON, LFO, SIM, COI, and MES). (**B**) Wave plot of the distribution of responses to the general risk item by decision pattern across all conditions. (**C**) Wave plot of the distribution of angles measured with the ring measure of social value orientation (SVO) by decision pattern across all conditions. Participants with lower angles are classified as competitive, with medium angles as individualistic, and with larger angles as prosocial. (**D**) Mosaic plot of the distribution of the five possible scores (0 to 4) on the cognitive reflection test (CRT) by decision pattern across all conditions. The height of rectangles corresponds to the relative frequency of scores in a given class, the width of rectangles to the relative frequency of the decision pattern in the sample. Rectangles intersected by the vertical line represent median values for the respective decision pattern.

We then analyzed differences across the four groups in terms of relative frequency across conditions, risk preference, social value orientation, and cognitive reflection (see Fig. 4). The relative frequency of decision patterns differed across conditions [χ^2^(15) = 60.08, *P* < .001; Fig. 4A]. Consistent with the results reported above, all-safe patterns were more frequent and all-risky patterns less frequent in the messaging, chain of infection, and simulator conditions. The learning from others’ outcomes condition showed the largest number of participants choosing a risky-safe strategy, consistent with the idea that they responded to but did not anticipate the rise in purple participants in the previous study. The four groups also differed in their responses to the general risk-taking item, with participants in the all-safe group reporting the lowest amount of risk-taking (*M* = 4.28) and participants in the all-risky group the highest amount [*M* = 6.06; *F*(3,596) = 12.89, *P* < .001, partial η^2^ = 0.06; see Fig. 4B]. Similarly, groups differed in terms of their social value orientation [*F*(3,596) = 12.09, *P* < .001, partial η^2^ = 0.06; see Fig. 4C]. Here, the estimated weighting angle for participants in the all-risky group was the lowest across groups (*M* = 19.07), indicating a tendency toward individualistic and competitive preferences. Last, participants in the risky-safe group scored highest (*M* = 2.87) on the four-item cognitive reflection test (*40*), and participants in the switch group scored lowest [*M* = 1.81; *F*(3,496) = 15.44, *P* < .001, partial η^2^ = 0.07; see Fig. 4D]. This is consistent with the finding that risky-safe strategies are associated with higher survival rates than switch strategies scoring the same number of points (see section E6).

### Postquestionnaire results in study 2

**Fig. 5.**
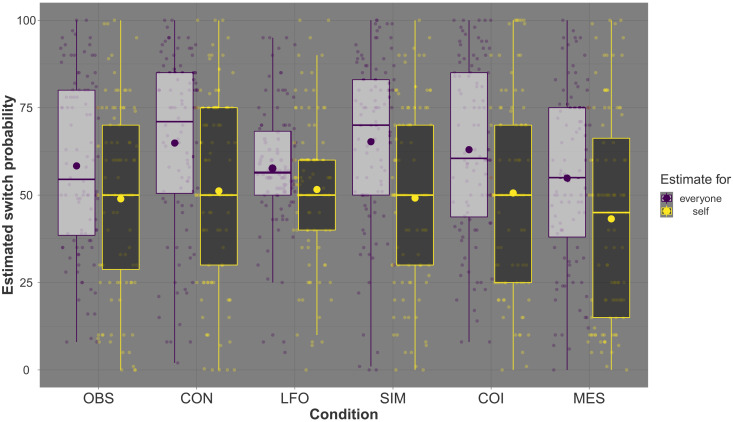
Estimates of one’s own and the average player’s probability of switching to purple by condition (OBS, CON, LFO, SIM, COI, and MES). Participants estimated their own probability of ending the game as purple, as well as that of the entire population. Small dots represent individual estimates (in percent). Boxes show quartiles, large dots represent mean estimates, and whiskers the value range (with a maximum length of 1.5 · interquartile range).

After the game, participants in all conditions estimated their individual risk of switching to purple as lower than that of the average participant [*F*(1,594) = 116.57, *P* < .001, η^2^ = 0.16; see Fig. 5]. They thus exhibited the same relative optimism that has been observed in studies on COVID-19–related risk perception (*41*). Consistent with our goal of designing a thematically neutral scenario (and thus avoiding distortions due to social desirability, politically motivated reasoning, etc.), only a minority of participants (<15%) made a connection between the game and the pandemic when directly probed for such a link. The neutrality of our framing shielded our interventions from having detrimental consequences for participants’ preventive health behavior outside the laboratory; such negative spillover effects have been observed in previous research (*22*, *23*).

## DISCUSSION

The most important result of our investigation is that the behavioral interventions varied widely in effectiveness. Although these differences would have been difficult to predict precisely in advance, they map onto some previous findings. For instance, some previous interventions focusing on descriptive norms have backfired and resulted in boomerang effects (*29*), in the same way as our observing behavior intervention. In the learning from others’ outcomes condition, taking risks was most pronounced in early rounds, consistent with previous observations that participants tend to anticipate a linear development in environments with exponential growth (*33*). As a result, they underestimated the consequences of earlier actions and only adapted to safer levels in later rounds. In contrast, both boosting interventions succeeded in decreasing the level of risk-taking, with the simpler chain of infection intervention proving more effective than the simulator intervention. A postexperimental questionnaire found that some participants struggled to process the information offered. Self-assessed level of comprehension of the two boosting interventions proved to be predictive of the interventions’ effectiveness, and the simpler of the two interventions reached a larger audience. Last, the messaging condition had the largest impact on behavior. This confirms the utility of communicating injunctive norms in shaping human behavior (*28*) and validates common practice by governments and health organizations in the COVID-19 pandemic. Note that the message communicated is more than a mere statement of a rule—it also offers moral reasons for following that rule.

In designing our transmission game, we chose a scenario that abstracted away some of the details of the real-world pandemic. This accomplished two purposes: First, we were able to insulate the game from the impact of real-world interventions that most participants would have experienced before the study. The neutral framing also buffered the study against the politicization of responses to the pandemic that affected some countries more than others and fluctuated throughout the months (*20*, *21*, *42*). We believe that this eliminated a serious threat to the replicability of results from studies using our paradigm. Second, we were able to reduce the complexity of the game to a level that did not overburden study participants, as evidenced by comprehension checks and open-format commentaries. Adding further elements to the game may have led to confusion and caused decision errors. Nonetheless, our game omits some general features of pandemics, including the dynamics of recovery, the differential distribution of health risks across segments of the population, and the stochastic nature of health outcomes. An interest in system-level effects specific to the current pandemic (e.g., threats to the health care system; work-from-home policies) would also require changes to the game format.

To maximize the number of independent observations, we implemented the scenario asynchronously without real-time interaction between participants. Previous attempts to implement synchronous interactions in larger groups with crowdsourced participants have experienced high attrition rates (*43*), with attrition likely concentrated in impulsive participants—who form a subgroup of interest when studying preventive behavior (*44*). The asynchronous format can be adapted to most testing conditions, and we present a complete paper-and-pencil variant in section E14.1. The asynchronous format does not allow the provision of feedback on color states at the time of decision-making, which limits its potential to test warning or contact tracing interventions (*19*). In section E14, we discuss several possible variants of the game that would be suitable for addressing a variety of additional research questions in the future. Furthermore, we analyze the performance of different strategies and the dynamics of transmission through a range of simulation scenarios (section E4). We also offer an analytic solution for populations with shared strategies or single individuals deviating from shared strategies (sections E5 and E6). These tools can be used to derive the final distribution of the number of purple participants and to conduct best-response analyses.

In 2005, players in an open-world multiplayer role-playing game experienced an unintended spread of a virtual infection causing fatal diseases in their avatars. It developed into a pandemic; multiple epidemiological mitigation strategies were attempted but failed, and game servers ultimately had to be reset (*45*). The simulated pandemic environment of our transmission game offers a more controlled testbed for the systematic study of behavioral interventions. The next steps would be to extend the set of behavioral interventions and study the effect of combining and fine-tuning interventions (e.g., combining messaging with a boost). Moreover, by changing its parameters and framing, researchers can easily reconfigure the transmission game to focus on particular psychological or economic factors. Interventions can be adapted for specific target groups and contexts beyond nonpharmaceutical interventions: As a case in point, vaccination decisions are emerging as pivotal in the battle against the pandemic (*46*). The population-wide uptake of vaccines may depend on the ability of behavioral interventions to foster cooperation and maintain preventive behaviors over a transitional phase (*47*, *48*, *49*).

Our results highlight the need to carefully test and select interventions aimed at encouraging the public adoption of preventive measures during a pandemic. We found evidence that normative messaging can reduce risk-taking behavior. However, the effectiveness of interventions aimed at boosting participants’ grasp of exponential transmission risks depended on how well they understood the intervention. This would suggest that a subgroup of recipients could benefit from a combination of normative and educative interventions. In contrast, interventions that provide information about others’ behavior or case rates did not reduce the amount of risk-taking, which casts doubt on the effectiveness of merely reporting daily case numbers to induce behavioral change. Our studies demonstrate the usefulness of pandemic-like simulations in the laboratory to safely and ethically test behavioral interventions at the beginning of a health crisis.

## MATERIALS AND METHODS

A more extensive description of game conditions, measures, and discussions of the methodology can be found in sections E3, E7, E9, and E10.

### Study 1

#### Participants

Study 1 analyzed the responses of 100 participants who played the transmission game without intervention. Of these, 55 categorized themselves as male, 43 as female, and 2 chose different categories. Participants were aged between 18 and 78 years (*M* = 30.7, *SD* = 12.23). Participants completed the survey between 14 May and 15 May 2020.

Participants were recruited via Prolific, with the following requirements being set: (i) participant location in the United States; (ii) no use of virtual private server (VPS), virtual private network (VPN), or proxy; (iii) availability of JavaScript to be executed within the survey; (iv) use of Firefox, Chrome, or Safari; and (v) a screen resolution of at least 800 × 600. In addition, we applied a number of quality control measures (see below).

#### Ethics

The study was approved by the Ethics Committee of the MPI for Human Development in Berlin (A 2020-16). Participants gave electronic consent after reading a consent form before beginning the study.

#### Tasks and measures

All measures are described in detail in the Supplementary Materials (see links in section S1), along with full texts and screenshots. Participants were instructed on the rules of the game and payments before playing. The game can be numerically described by nine parameters (see Fig. 1 and Table 2). The number of rounds (1), the transmission probabilities (4 to 6), and the values of the action (7 to 8) were shown at each round in the game, along with the accumulated points and potential payoff.

**Table 2.** List of parameter values.

**Number**	**Parameter**	**Value**
1	Number of rounds	25
2	Number of participants	100
3	Number of purple participants at the start	8
4	Transmission probability: GG	0.05
5	Transmission probability: GH/HG	0.15
6	Transmission probability: HH	0.25
7	Value of action G	8
8	Value of action H	40
9	Points per GBP	200

In a postquestionnaire, participants were asked to describe their game strategy, to answer questions about their goals and motivation in the game, and to estimate both their own and the other players’ behavior and likelihood of switching to purple. They were also asked about similarities between the game and their previous experience and invited to send hypothetical messages to different player types and to comment on the game in general.

Study 1 also contained a broad range of further measures and items. In particular, participants completed a number of personality and cognitive measures, as well as measures assessing political attitudes, economic preferences, and health-related questions.

The personality measures included the 24-item Brief HEXACO Personality Inventory [with the six scales; Honesty-Humilty (H), Emotionality (E), Extraversion (X), Agreeableness (A), Conscientiousness (C), and Openness to Experience (O)] (*50*), the refined 11-item version of the Hong Psychological Reactance Scale (*51*), the 9-item Oxford Utilitarianism Scale (*52*), and a 16-item measure of the dark core of personality (*53*).

Cognitive measures included a four-item version of the cognitive reflection test (*40*), combining items from two sources (*54*, *55*), a four-item measure of understanding exponential growth developed by the authors, the four-item Berlin Numeracy Test (*56*), and a brief three-item measure of subjective numeracy (*57*).

Political attitudes were measured with the 12-item Social and Economic Conservatism Scale (*58*) and with single items asking whether participants had registered to vote and tapping attitudes toward presidential candidates (in 2020) (*59*), political position, party affiliation, and religiosity. Climate change skepticism was measured with six items (*60*, *61*).

The survey was complemented by three incentivized economic games: the dictator game (*62*), a money-burning task (*63*), and a variant of the die-under-the-cup task (*64*). Participants further completed the six-item version of the ring measure of social value orientation (*39*) and four single-item measures tapping general risk preference (*65*), trust, time preference (*38*), and impatience (*66*).

Health-related questions included a measure of subjective life expectancy (*67*) and a general health question (*68*). We also implemented questions tapping COVID-19–related attitudes and behaviors, including measures of worries, compliance with mitigation measures, attitudes toward social distancing, belief in misinformation (*69*, *70*), and acceptance of tradeoffs. Participants were also asked to comment on the COVID-19 crisis in general. We collected data on age, gender, education, household income, and employment status.

#### Incentives

Participants received both a fixed compensation of GBP 5.00 and a variable bonus payment that depended on their decisions in four parts of the study. The transmission game was simulated just once to determine participants’ payoffs (in contrast, the reported results are averages across many simulations): They received GBP 0.01 per 2 points scored if their color was still blue at the end of that simulation (resulting in a bonus payment of up to GBP 5.00 for this game). Participants also received bonus payments for their choices in the dictator game, the money-burning game, and the dice game. We sent a message via Prolific to each participant about their result. Final payments ranged between GBP 5.00 and GBP 10.54 (with an average of GBP 6.31).

#### Data quality protocol and sample selection

Given various reports of problems with data quality on Prolific at the time of the study, we implemented a number of protective measures in the setup of our survey. First, we verified the location requirement in multiple steps: (i) The browser location was checked via JavaScript and Qualtrics. (ii) Two services were used to flag responses for suspicious IP addresses indicating the use of VPN connections and VPS (iphub.info and proxycheck.io). (iii) Participants had to enter the abbreviation of the U.S. state they were in. (iv) Participants had to pass two attention checks (drawn from a larger set) at the beginning of the study. (v) Participants had to pass the comprehension checks with an acceptable score (see links in section S1 for details).

An initial total of 131 participants started the task. Of these, 25 were excluded at the beginning of the study: Two participants were flagged due to suspicious IP addresses (possible proxy use or VPN server), 3 participants did not respond to initial attention check questions, and 20 participants failed at least one of the two attention checks. Of the 106 participants who passed these checks, 4 dropped out before the game or during the instructions, and 2 did not continue after having completed the game.

We assessed the distribution of errors in comprehension checks after 100 participants had completed the survey. Two outliers (with 50 and 71 errors, respectively; at least twice as many errors as anyone else) were excluded and replaced by two new participants. For study 2, we hardcoded a threshold of 25 into the survey flow.

### Study 2

#### Participants

Participants were recruited via Prolific, with the same requirements being set as in study 1. We prevented double participation via Prolific’s option to block those who had participated in selected previous studies. Again, we applied a number of quality control measures (see below).

In study 2, we analyzed the responses of 600 participants who played the transmission game in groups of 100 across the six conditions: learning from others’ outcomes, observing behavior, messaging, simulator, chain of infection, and a control condition without intervention. Of these, 299 categorized themselves as male (49.8%), 297 as female (49.5%), 3 chose different categories, and 1 preferred not to answer the question. Participants were aged between 18 and 75 years (*M* = 32.6, *SD* = 12.39). Participants completed the survey between 19 May and 21 May 2020.

#### Ethics

The study was approved by the Ethics Committee of the MPI for Human Development in Berlin (A 2020-16). Participants gave electronic consent after reading a consent form before beginning the study.

#### Tasks and measures

All measures are described in detail in the Supplementary Materials (see links in section S1). In study 2, participants played the transmission game in one of six conditions. Intervention conditions included condition-specific instructions and condition-specific postquestionnaires in addition to a general postquestionnaire.

Moreover, study 2 included a subset of the measures administered in study 1, with some variations. Personality measures were reduced to the Brief HEXACO Personality Inventory [with the six scales; Honesty-Humilty (H), Emotionality (E), Extraversion (X), Agreeableness (A), Conscientiousness (C), and Openness to Experience (O)] (*50*) and the Hong Psychological Reactance Scale (*51*). The Emotionality and Honesty-humility subscales from the Brief HEXACO Inventory were replaced by the 10-item versions from the HEXACO-60 (*71*). Cognitive measures were limited to the cognitive reflection test (*40*, *54*, *55*). We again administered the Social and Economic Conservatism Scale (*58*); single-item measures tapping attitudes toward presidential candidates (*59*), political position, party affiliation, and religiosity; single-item measures of risk-taking (*65*), trust, and time preference (*38*); and the ring measure of social value orientation (*39*). COVID-19–related questions included measures of worries, compliance with mitigation measures, and the acceptance of tradeoffs. We assessed the same demographic variables as in study 1.

#### Incentives

Participants received both a fixed compensation of GBP 2.25 and a variable bonus payment for their decisions in the transmission game (the only part of study 2 affecting the bonus payment). The transmission game was simulated just once for each condition to determine participants’ payoffs. They received GBP 0.01 per 2 points scored if their color was still blue at the end of that simulation (resulting in bonus payments of up to GBP 5.00). Given the substantial number of participants who switched to purple, we added a symbolic GBP 0.10 without prior announcement to each participant’s bonus payment and sent a message via Prolific about the outcome. Final payments thus ranged between GBP 2.35 and GBP 7.35 (with an average of GBP 3.39).

#### Data quality protocol and sample selection

The data quality procedure was the same as for study 1 with one exception: For study 2, a cutoff of 25 errors in the comprehension checks was hard coded into the survey flow so that participants with more than 25 errors were not able to proceed to the game. This is consistent with the policy implemented for study 1 (see the Supplementary Materials for details).

The task page was accessed 897 times in total. Of these, 238 participants were excluded at the beginning of the study: 26 participants were flagged because of a suspicious IP address (possible proxy use, VPN server, or compromised server; the two IP checkers disagreed only in one of these cases), 20 did not submit their attention check responses, 185 failed at least one of the two attention checks, 2 were not located in the United States, 4 did not give valid answers to the question about their state abbreviation (with false answers as “United States of America” or “COVID-19”), and 1 participant did not give consent and left the study.

Of the 659 participants who passed these checks, 25 dropped out during the general game instructions, 4 during the simulator instructions, and 3 after the transmission game but before the end of the survey. A total of 21 participants committed too many errors in the attention checks (25 or more) and were automatically prevented from further participation (and asked to return the task). A further four participants were identified as having completed part of the survey twice (e.g., reloading the survey on a different device after interrupting their first attempt). Even if the second attempt was completed, these participants were excluded from the analysis. The participant who took longest to finish the survey gave inconsistent or incomprehensible responses throughout the task (e.g., espousing extremely liberal and conservative positions simultaneously). These problems were identified while the study was active, and these participants’ places in the conditions were filled by later participants. Last, the randomization algorithm assigned one participant to the wrong condition at the very end of the study. We collected one additional participant in the correct condition and excluded the additional data point from the analysis (this was decided before the participant had completed the task and followed the preregistered protocol).

### Simulations

The simulations underlying the results shown in Fig. 2 are part of a more extensive set that we conducted to investigate theoretical properties of the transmission game and probe the stability of empirical findings. All simulations were conducted in Matlab with usually 1,000,000 trials per simulation condition. The simulation code and result summaries are presented in the Supplementary Materials (see links in section S1). For example, we investigated the impact of different levels of risk-taking and compared different strategies of switching between actions. Results illustrate the social dilemma at the core of the game and how the risky-safe pattern of distributing risky actions is superior to alternatives. We show result distributions for all games based on empirical data and demonstrate the robustness of results in respect to resampling.

### Analytical tools

We developed analytical tools to calculate the probability distribution of possible final states for special cases of games. We programmed these tools in Matlab for two possible types of scenarios: One type in which every simulated player chooses the same strategy, and one type in which a single individual deviates from the shared strategy. We were able to cross-validate analytical and simulation results and run best-response analyses that we present in the Extended Supplementary Materials with code and results.

## Supplementary Materials

### This PDF file includes:

Sections S1 to S8 Figs. S1 to S62 Tables S1 to S3

## Notes

View/request a protocol for this paper from *Bio-protocol*.
