# Supplementary material for: The transmission game: Testing behavioral interventions in a pandemic-like simulation
Source: Sci Adv. 2022 Feb 25;8(8):eabk0428. doi: 10.1126/sciadv.abk0428 (PMC8880790; doi:10.1126/sciadv.abk0428)
Supplement: Supplementary file 1 — Sections S1 to S8 Figs. S1 to S62 Tables S1 to S3 [file sciadv.abk0428_sm.pdf]

Science Advances
AAAS

Supplementary Materials for

The transmission game: Testing behavioral interventions in a pandemic-like simulation

Jan K. Woike*, Sebastian Hafenbrädl, Patricia Kanngiesser, Ralph Hertwig

*Corresponding author. Email: woike@mpib-berlin.mpg.de

Published 25 February 2022, *Sci. Adv.* **8**, eabk0428 (2022)
DOI: 10.1126/sciadv.abk0428

This PDF file includes:

Sections S1 to S8
Figs. S1 to S62
Tables S1 to S3

S1 Organization of the Supporting Material

S1.1 Contents of different documents

The full supporting material consists of nine separate documents with this document serving as an overview document. The other eight documents are the following (please follow the links to reach the relevant repository):

1. Extended Supplementary Materials (**E**): `https://doi.org/10.7910/DVN/ILDEM0`

2. Study Materials Study 1 (**SM1**): `https://doi.org/10.7910/DVN/RHR2A5`

3. Study Materials Study 2 (including JavaScript code; **SM2**): `https://doi.org/10.7910/DVN/ILDEM0`

4. Variables and Measures Study 1 (including R code; **VM1**): `https://doi.org/10.7910/DVN/RHR2A5`

5. Variables and Measures Study 2 (including R code; **VM2**): `https://doi.org/10.7910/DVN/ILDEM0`

6. Supporting Analyses Study 1 (including R code; **SA1**): `https://doi.org/10.7910/DVN/RHR2A5`

7. Supporting Analyses Study 2 (including R code; **SA2**): `https://doi.org/10.7910/DVN/ILDEM0`

8. Simulation Code and Results (including R and Matlab code; **SCR**): `https://doi.org/10.7910/DVN/ILDEM0`

The Extended Supplementary Materials add to the Materials in this document, in particular with the following additions:

- An extended presentation of study goals and framework with references to the literature that motivated our choices,

- an extended discussion of the framework development process, including literature-based justifications of design decisions,

- additional simulation results,

- the development of analytical tools for calculating expected results of special cases of the game,

- additional analytical results related to best responses to group strategies and equilibria,

- additional results for Study 1 and Study 2,

- an extended discussion of results, and

- variants of the Transmission game, inlucding a paper-and-pencil variant, parameter variations, and additional components.

There are two documents each (one for each Study) for study materials, scales, and analyses. A seventh document contains the code and results for all simulated games. Study materials include all item texts and screenshots of many parts of the study as they were shown to participants. The documents focusing on variables and measures present inter-item correlations and Cronbach's alphas for scales used in the study and document sources for variables. These documents were compiled using knitR within Overleaf, and contain the full R code used to create results using the raw data files. The documents with supporting analyses were created with knitR as well and likewise include all written code used to generate results and figures. Finally, the simulation documents provide the Matlab code used for simulations, and contain the R code for analysing the simulations' output files. This document was compiled directly from RStudio.

S1.2 File location, reproducibility and code

All other Supplementary Material documents are hosted on the Harvard Dataverse in two Dataverses together with all data referenced and used within these documents. For Study 1 the data set and documents (356 pages) are published as:

J. K. Woike, S. Hafenbrädl, P. Kanngiesser, R. Hertwig, Replication Data for: Study 1 (The Transmission Game: Testing behavioral interventions in a pandemic-like simulation), *Harvard Dataverse*, V1, 2021, https://doi.org/10.7910/DVN/RHR2A5.

1. **SM1**: 113 pages
2. **VM1**: 181 pages
3. **SA1**: 62 pages

For Study 2 the data set and documents (756 pages) are published as:

J. K. Woike, S. Hafenbrädl, P. Kanngiesser, R. Hertwig, Replication Data for: Study 2 (The Transmission Game: Testing behavioral interventions in a pandemic-like simulation), *Harvard Dataverse*, V2, 2021, https://doi.org/10.7910/DVN/ILDEM0.

1. **SM2**: 128 pages
2. **VM2**: 124 pages
3. **SA2**: 96 pages
4. **SCR**: 162 pages
5. **E**: Extended version of the Supplemnentaty Materials, 246 pages

S1.3 Selection for this document

The information in this document is mostly a selection of results taken from the seven other documents. We added a short section detailing the game rules and discuss some possible game variants in the final section. We present the material related to the game framework (Study 1) and the five interventions (added in Study 2), omitting comprehension checks and postquestionnaires (see **SM1** and **SM2**). We added a brief description of selected explanatory variables.

We present results supporting the selection of scales from Study 1 that were subsequently implemented in Study 2 (for more details about all measures see **VM1** and **VM2**). We briefly discuss the relationship between these predictors, game results, political preferences and reactions to COVID-19 (for more results see **SA1** and **SA2**).

As the game rules contain stochastic components that will potentially change outcomes in repeated play-throughs of a game with given choices, we use the simulation method to illustrate likely outcomes and their distribution across 1,000,000 simulations per condition, both for the actual data and for theoretical data. The latter helps to illustrate maximizing and dominated strategies and the social dilemma at the core of the game. The theoretical results also provide context for evaluating the empirical results (for more simulations and results, see **SCR**).

We connect our approach to many recent studies focused on the current pandemic. Due to the relatively short amount of time since the beginning of the pandemic, many of these studies have not been peer-reviewed at this stage. We do not understand our referencing as an unconditional endorsement of these studies, but rather as an attempt of helping to connect independent strands of research and documenting parallel findings and inter-relationships.

S1.4 Links to preregistrations

We preregistered the materials for both studies on the Open Science Foundation platform. Please find links to the two preregistrations below:

- Study 1: `https://osf.io/kc2an/`,
- Study 2: `https://osf.io/uacfy/`.

S1.5 Intervention names in main text and Supplementary Materials

After completing the Supplementary Materials, we decided to change the names of our intervention conditions to render them more intuitive for a multidisciplinary audience. We did not change the names in the Supplementary Materials, though, as they appear in variable names and throughout the pre-registration, in figures and results files. Table S1 presents the mapping from names and abbreviations used in the manuscript to names and abbreviations used throughout the Supplementary Materials (including this document) and code files.

Table S1

Mapping of names and abbreviations of interventions in the manuscript to names and abbreviations in the Supplementary Materials.

Manuscript			Supplement	
Intervention	Short name		Intervention	Short name
Learning from others' outcomes	LFO	⟶	Vicarious learning	VIC
Observing behavior	OBS	⟶	Descriptive norms	DES
Messaging	MES	⟶	Injunctive norms	INJ
Simulator	SIM	⟶	Simulator	SIM
Chain of Infection	COI	⟶	Chain	CHN

S2 The Transmission Game Framework

S2.1 Brief summary of game paradigm and chosen interventions

S2.1.1 Rationale for the construction of the transmission game paradigm

Our study proposes a novel paradigm to experimentally study decision-making in pandemic-like scenarios and the effectiveness of intervention measures. In developing the transmission game paradigm, we were guided by a list of desirable properties that we wanted to achieve by design: We wanted to capture central elements of the health crisis and introduce them in a neutrally-framed game scenario. Participants in large groups were to encounter the equivalent of a spreading infection among their group and face a social dilemma. Individually profitable actions as opposed to safer and less profitable actions should dynamically increase the risk for self and others to incur severe losses at the end of the game. We intended the game paradigm to be communicable to broad parts of the population and to be flexible enough for groups of various sizes (including very large groups), both offline and online. In addition, the paradigm should be deception-free (*64*). We wanted participants to make choices independent of each other to increase the statistical power of group comparisons. Finally, we wanted to create a paradigm that would allow us to test a range of interventions. We explain and justify details of game and interventions in the Extended Supplementary Materials (Section E3 and Section E9, `https://doi.org/10.7910/DVN/ILDEMO`).

S2.1.2 Choice of interventions for Study 2

We implemented five different interventions in Study 2. Specifically, we included a vicarious learning intervention showing the percentage of "infected" participants in a previous game (Study 1) up to the round for which a decision had to be made. Further, we tested two norm-based interventions focusing on how many participants chose the low risk option in the previous game and a brief message explaining the negative consequences of choosing the higher risk option for oneself and other players. Finally, we included two different boosting interventions (1) highlighting how high-risk choices can initiate cascading consequences and result in transmission chains of infection and (2) illustrating the game dynamics through a simulator of game choices and outcomes.

S2.2 Study goals

S2.2.1 Goals of Study 1

In Study 1, we implemented the paradigm without interventions in a single group of 100 participants on Prolific, all located in the United States. In addition to the game, we included a postquestionnaire, a broad range of possible predictors, and several survey scales measuring COVID-19-related attitudes. The preregistered study had three main goals: (1) demonstrate that participant behavior shows substantial variance, (2) collect data for realizing some of the interventions planned for Study 2, and (3) perform an exploratory analysis of predictors and select a promising subset for Study 2.

S2.2.2 Goals of Study 2

In Study 2, we compared game behavior in a control condition (between-subjects) with behavior in five intervention groups (one group per intervention). A total of 600 players participated on Prolific, all located in the United States. There were two main goals in Study 2: (1) Estimate the relative effectiveness of the five interventions in reducing the number of risky decisions by participants, and (2) attempt to replicate the findings for predictor variables in Study 1.

S2.3 Rules of the standard transmission game

For easy reference, we offer a brief summary of the rules at this point. We also reproduce the instructions given to participants in the "Selected Materials" section in this document.

We will start with a brief list of all rules and then explain their implications in more detail.

1. There are 100 players in the game.
2. Each player is in one of two states, blue or purple.
3. No player can see their own state or the state of other players.
4. A player can change from blue to purple but a purple player stays purple until the end of the game.
5. At the start of the game, all 100 players are blue.
6. The game lasts 25 rounds.
7. Before the first round only, eight randomly selected players change into purple players.
8. At the beginning of each round, players are paired at random.
9. Each player chooses an action, G or H for the round.
10. A player who chooses G receives 8 points, a player who chooses H receives 40 points.
11. A blue player that is paired with a purple player may change into a purple player.

12. If none of the paired players chooses H, a blue player that is paired with a purple player changes into a purple player with $p = 0.05$.
13. If any one but not the other of the paired players chooses H, a blue player that is paired with a purple player changes into a purple player with $p = 0.15$.
14. If both players choose H, a blue player that is paired with a purple player changes into a purple player with $p = 0.25$.
15. The game proceeds until the end of round 25.
16. If a player is blue at the end of the game, the their points scored across the 25 rounds are converted into a bonus payment at a rate of 2 points: 1 penny [0.01 GBP].
17. A purple player does not receive a bonus payment; scored points are thus valueless for purple players.

It should be noted that a further rule is implicit due to the asynchronous nature of play: No player observes the actions of other players. This conveys the two benefits discussed above (i.e., the paradigm is scalable to groups of different sizes, and independent choices allow for more statistical power).

S2.4 Illustrations of game rules and implications

To illustrate the game rules and their implications, we will use several illustrations of players and populations. We will represent players by blue and purple stones (see Fig. S1).

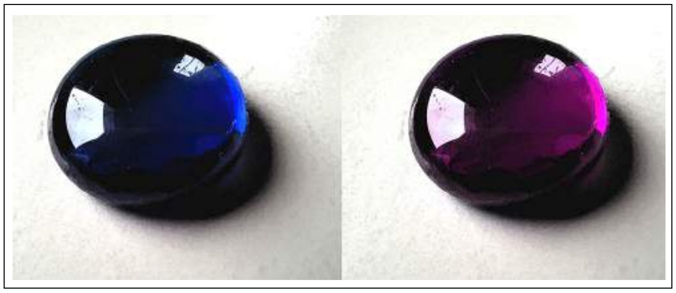

Figure S1
Representation of players: two possible states.
Photo Credit: Jan K. Woike, University of Plymouth.

Every player starts the game in the blue state, and the initial population is therefore represented by 100 blue stones (see Fig. S2, left side). Before the first round, eight randomly chosen players change their color to purple (see Fig. S2, right side).

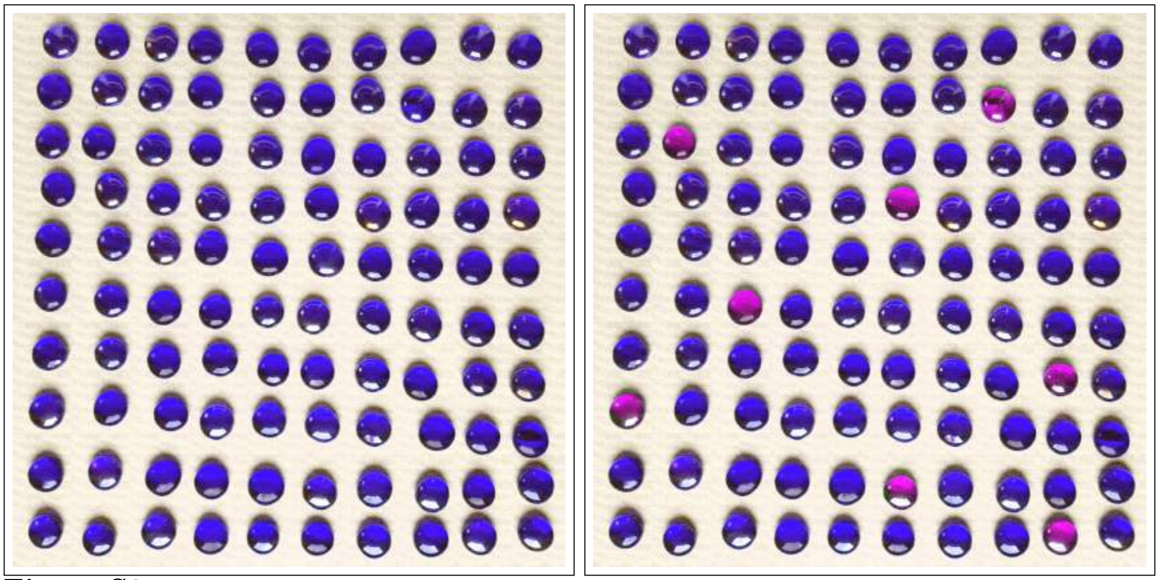

Figure S2

Population at the start of the game (left side) and before the first round (right side).

Photo Credit: Jan K. Woike, University of Plymouth.

The pairing of players in each round corresponds to randomly rearranging the stones in rows of two (see Fig. S3, left side). Each pairing results in one of four combinations of stone colors: blue/blue, blue/purple, purple/blue, or purple/purple. For two of these combinations, the actions chosen by the players have no impact on their color at the end of the round (see Fig. S3, right side): Two paired blue players will stay blue and two paired purple players will stay purple. Their action choice will still have consequences for the points they receive in the round: Players choosing action G receive 8 points, players choosing action H receive 40 points. Note that receiving points does not equate to receiving bonus money. If both players are purple, their points will ultimately not be converted into bonus payments.

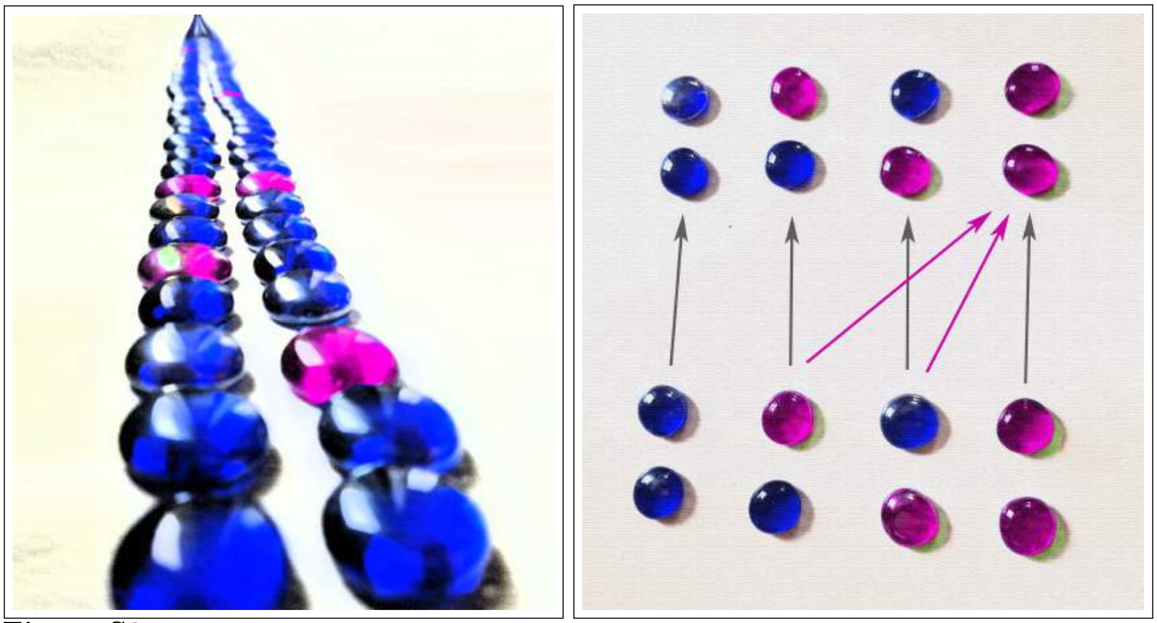

Figure S3

Pairing of players (left side) and possible color change (right side).

Photo Credit: Jan K. Woike, University of Plymouth.

A color change can only occur, if a blue player is paired with a purple player. In this case, the blue player may change into a purple player (not the other way round). The minimum probability is $p = 0.05$ (if both choose action G), but it increases by 0.1 for each H-action chosen by any of the two players up to a maximum of $p = 0.25$ (see Fig. S4).

	$\mathbf{G}_j$	$\mathbf{H}_j$
$\mathbf{G}_i$	5%	15%
$\mathbf{H}_i$	15%	25%

Figure S4

Probability of switching from blue to purple conditional on player actions.

These are all of the game mechanics. Figure S5 illustrates how a game could progress to the final round. Note that the stones representing specific players remain in fixed positions and purple players stay purple until the end of the game.

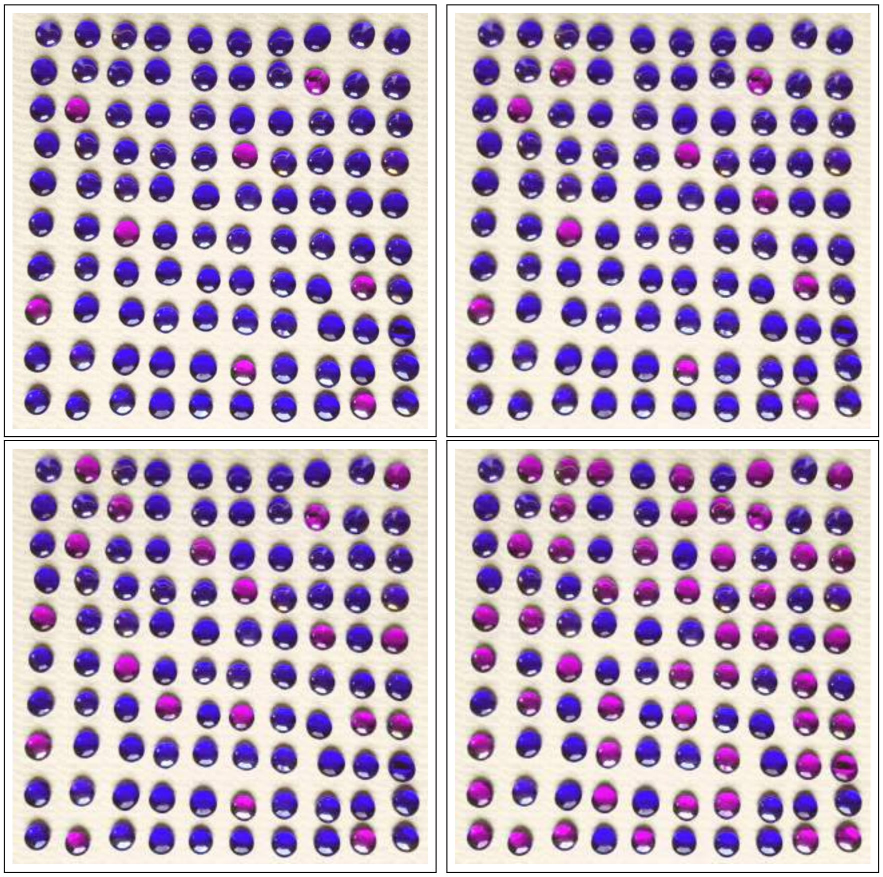

Figure S5

Progression of the game in four steps to the end (from top left to bottom right): Stone colors are shown after rounds 1, 5, 15, and 25.

Photo Credit: Jan K. Woike, University of Plymouth.

S2.5 Development of the framework

Our goals in developing the Transmission game framework were the following:

1. Map relevant elements of the COVID-19 health crisis onto game elements.
2. Establish a neutral and non-politically charged context.
3. Create a paradigm that is communicable to the general public, comprehensible and simple enough.
4. Design the paradigm to be deception-free.
5. Be able to apply this paradigm to groups of various sizes including very large groups.
6. Be able to collect data online and offline.
7. Collect data from individuals independently, preserving power for the analysis.
8. Design the paradigm to be flexible enough to test multiple forms of interventions.

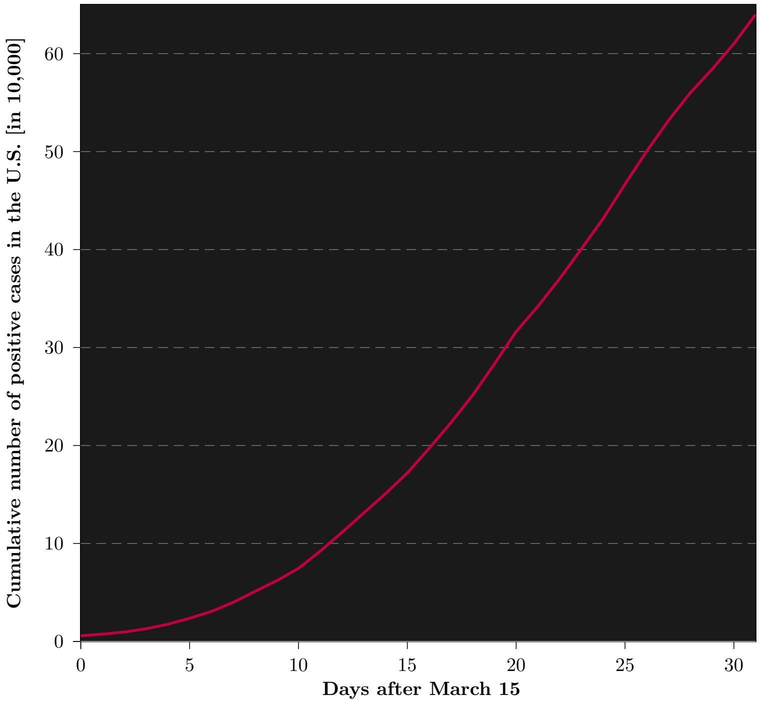

Figure S6
Cumulative number of positive cases in the United States since March 15, 2020, based on the data provided by the COVID Tracking project.

S2.5.1 Relevant features of the health crisis

From the beginning our focus was on the complex dilemma involved in mitigating the effects of the novel coronavirus. Social distancing, mask-wearing, and taking individual precautions are costly, both for the individual and organizations. At the same time, the consequences of not engaging in these and other counter-measures are costly as well. Violations of preventive rules can provide benefits to the rule violators, but incur costs on others who are negatively affected by it.

In addition, the implementation of counter-measures poses a collective action problem in the sense that they are personally costly but will remain ineffective if an insufficient number of people adheres to them. At the same time, widespread adherence might confer benefits even to isolated violators.

Another relevant aspect of the current crisis is the exponential nature of virus transmissions. At the beginning of the pandemic in the United States, the number of observed cases since the first case followed a clearly non-linear pattern. Figure S6 shows the development of total case numbers for the days following the 15th of March 2020.

Linear extrapolation of infection fails to appreciate the risks associated with rising numbers and accelerated growth.

S2.5.2 Mapping these features

Mapping the features of the current crisis onto elements in the game involved both a positive selection of features to include and a negative selection of features not to map. We decided to exclude a number of elements that play a role in the current crisis to keep the game rules at a level accessible by a typically motivated survey participant. While we made this selection for the standard paradigm, there is no reason not to include (some of) these features in future variants of the game. We would advise not to include everything at the same time, though. We believe that the current level of complexity should not be exceeded by more than a few additions.

Health status. To move from a health domain that—given the current political discourse—intersects with the political domain into a more neutral territory, we chose to simplify the scenario by replacing health status by a simple pair of colors: blue and purple. Blue represents a healthy individual, purple represents an infected individual. We did not choose green and red (*16*), as these colors have multiple connotations due to their use in warning signs and standard icons.

Binary state. We simplified the complex medical relationships by reducing health status to these two states.

In addition, we reduced the number of state transitions to a single possibility: blue participants can change to purple but not the other way round. A purple participant stays purple until the end of the game. Thus, we excluded one feature present in the current crisis, namely the possibility of recovering from the infection (*26*).

Consequences of color change. Consequences range from mild discomfort to death, but we simplified all of this in the game to a single outcome: Changing one's color to purple implies losing all potential bonus money for the game. Obviously, these consequences are less severe than the consequences of contracting the actual virus, but they correspond to the maximum level of negative consequences that can be ethically justified for participants in our experimental setting.

Time period and initial state. To study transmission we created a game with multiple rounds that starts with a low proportion of purple participants. This corresponds to the state of nations at the beginning of the epidemic after small numbers of positive cases were reported. We chose a length of 25 rounds to allow for the potential of considerable spread.

Interactions and transmission. We simplified virus spread through a population by only considering dyadic interactions. In each round, players are grouped in pairs and have one interaction in their pair, resulting in a total of 50 interactions per round (in a population of 100 players). Furthermore, we only allow color changes when a blue player is paired with a purple player, and only from blue to purple.

Protective measures and points. The risks of any real-world interaction can be mitigated by protective measures. While several types of these measures (e.g., mask-wearing, social distancing, hand-washing) exist, we simplified the choice to engage in these mitigating behaviors to a binary choice. To reduce moral and political connotations, we offer two abstract actions, namely "G" and "H". Action "G" represents a choice in favor of mask-wearing and social distancing, and action "H" represents a choice against these measures. Protective measures incur costs in terms of materials, time, and restricted opportunities to engage with others both for personal and commercial purposes. To mirror these differential

costs, we assigned a score of 8 to the restricted behavior (action G) and a score of 40 to the unrestricted behavior (action H). All players choose one of these two actions in each round of the game.

Protective measures and transmission risk. To map the relation between risk-mitigating behavior and transmission, we implemented transmission risk from a purple to a blue player as a function of both players' action choices. If both players choose action G, the blue player has a risk of 5% to turn purple. If one of the two players chooses action H and the other player chooses action G, the risk of turning purple increases by 10% to 15% (it does not matter which player chooses H). If both players choose action H the risk of turning purple increases to the maximum of 25%. We chose this value as it reflects the fact that risky interactions do not result in an infection as the most likely outcome, even for an infectious disease such as COVID-19. Even after our data collection was completed, data on the effectiveness of protective measures only allowed for estimates with a high degree of uncertainty.

Non-linear development. To create the potential of non-linear growth of the number of purple participants in the population, several requirements have to be met. The population has to be large enough to allow for a gradual acceleration of the infection rate until the majority of the population is infected (and a slowing convergence against total infection afterwards). Simultaneously, we needed a game that lasts long enough for these phases to be reached. To meet these requirements, we finally chose an initial rate of 8% to achieve a compromise: Switching to purple before the first round stays relatively unlikely, and we were able to keep the maximum color change risk at 25% and the number of rounds at 25. The simulation results for these parameters (see **SCR** and the simulation section below) confirm that this creates the desired progression in purple rates.

S2.5.3 Asynchronous implementation in an online setting

We designed the paradigm to be implementable in an asynchronous online setting. We considered asynchronous gameplay as the preferable option to avoid both sample attrition and losing power through non-independence of observations. We also believe that reduced demands regarding technology lower the barriers for those who are interested in replicating the work. This can include those working inter-culturally and in environments with restricted access to technology. For this purpose, we also discuss a paper-and-pencil variant of the game in the Extended Supplementary Materials (Section E14.1, `https://doi.org/10.7910/DVN/ILDEM0`).

S2.5.4 No deception

It is crucial for us that implementations of the paradigm are deception-free and we consider it unacceptable to use fake participants, fake choices by other players, etc. We reject the use of deception both for ethical and pragmatic reasons.

S2.5.5 Balance between complexity and comprehension

As we have described throughout this section, there were several components of pandemics that could be added to a game scenario, but that we decided not to include. The reason for this decision is that we needed to strike a balance between realism and

Table S2

List of parameter values

Number	Parameter	Value
1	Number of rounds	25
2	Number of participants	100
3	Number of purple participants at the start	8
4	Transmission probability: GG	0.05
5	Transmission probability: GH/HG	0.15
6	Transmission probability: HH	0.25
7	Value of action G	8
8	Value of action H	40
9	Points per GBP	200

comprehensiveness on the one side and feasibility and understanding on the other side. We decided to develop a paradigm that can be used with diverse samples, not a selected group of trained university students in technical disciplines.

Parameters in the basic game. Our final design can be fully characterized by nine parameters (see Table S2).

The number of rounds (1), the transmission probabilities (46), the values of the action (78) were shown at each round in the game. The conversion rate is applied to each score, indicating the possible payoff for the points scored up to the current round. Thus, there is no need to memorize numbers with the exception of the population size and the initial color switch rate (which is asked for in comprehension questions and clearly stated in the instructions).

S3 Summary of Theoretical Simulation Results

S3.1 Degree of cooperation and color switching rates

Choosing action H can be considered a competitive action and choosing action G a cooperative action. Choosing H increases the risk of color change (blue to purple) and thereby the risk of ending the game without bonus payment. At the same time, choosing action H increases a player's point score substantially, without producing any benefit for other players. Choosing action G lowers the risk of color switching both for the player themself and other players.

To explore how the game plays out if different numbers of players choose cooperative and competitive actions, we conducted several sets of simulations (see **SCR** for code and results for all of these simulations). In a first set, we determined a fixed proportion of players who would consistently choose H-actions (AllH) with the remaining players choosing G-actions for every round in the game (AllG). By systematically varying the proportion of AllH-players, we were able to observe how the simulation would play out depending on the amount of cooperation the group.

Figure S7 summarizes the results of 1,000,000 games with 50% AllH and 50% AllG players. Many elements in the game are stochastic in nature, but results across games are centered around a trajectory of small increases of the proportion of purple players in the beginning, accelerated growth in the middle of the game and some degree of slowing down in the final rounds. This dynamic is due to a combination of exponential infection dynamics with a finite population. That is, the number of purple participants starts at 8% and each color switch leads to an acceleration of further color switching until half of the population is purple. Beyond this point, there will be fewer pairings of blue with purple players.

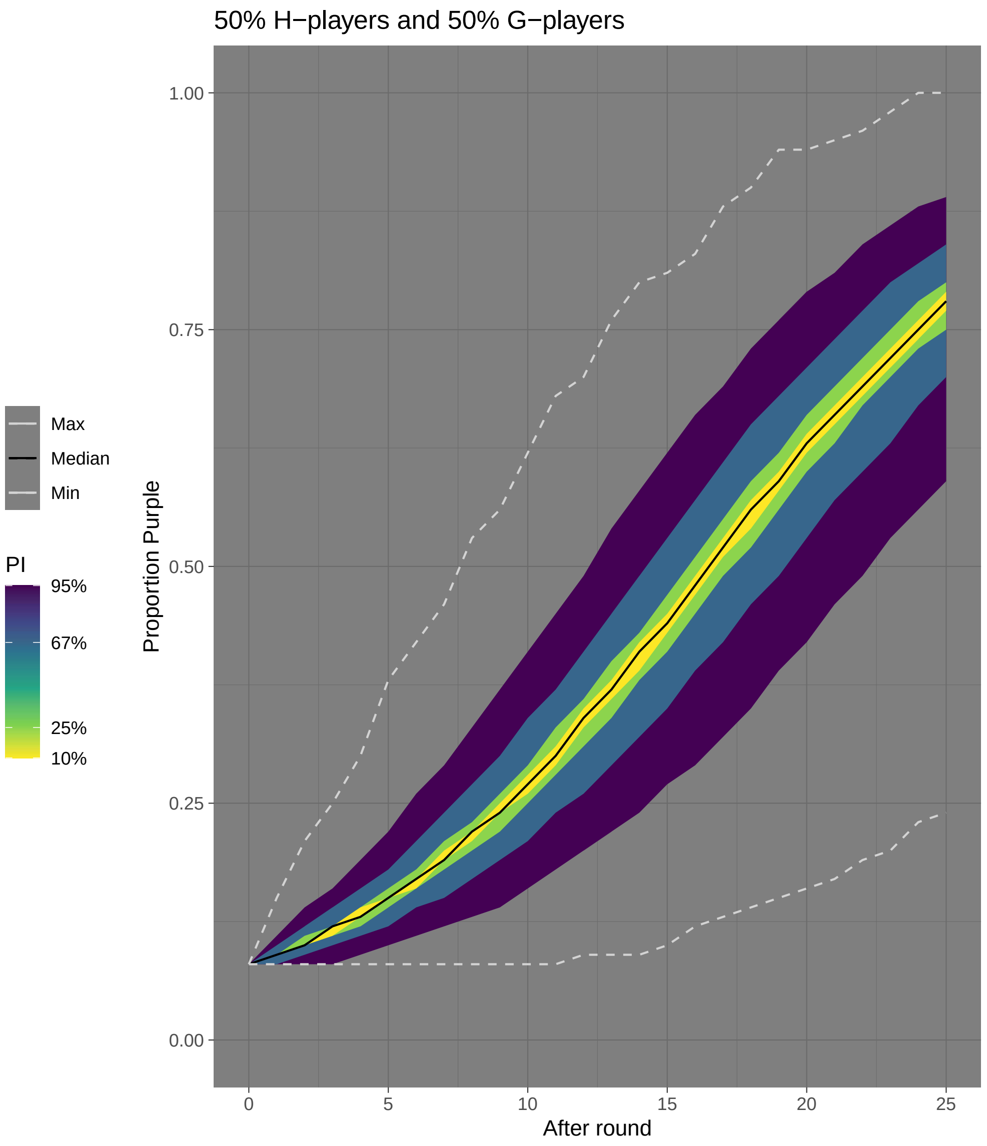

Figure S7

Distribution of observed proportions of purple participants by round across 1,000,000 games with 50% ALLG-players and 50% ALLH-players: lines mark the median, minimum, and maximum results, and areas show the inner 10%, 67% and 95% of results.

Varying the percentage of AllH players systematically from 0 to 100% in 20 steps and simulating 1,000,000 games for each percentage, reveals a clear pattern in the mean proportion of purple participants across rounds (see Fig. S8). The expected final proportion of purple participants is very sensitive to the number of risk-taking players in the group, starting from well below 0.25 and going up to over 0.95 (less than five players stay blue on average, in the latter case). Color switching can at no point be completely avoided, but it can certainly be reduced by collective cooperation. For lower percentages of AllH players final proportions of purple players change in larger increments as these groups do not face the growth limits incurred when half of the population has already switched colors. In contrast, in the upper range of proportions of AllH players a large majority of the population has already switched to purple in the final rounds of the game.

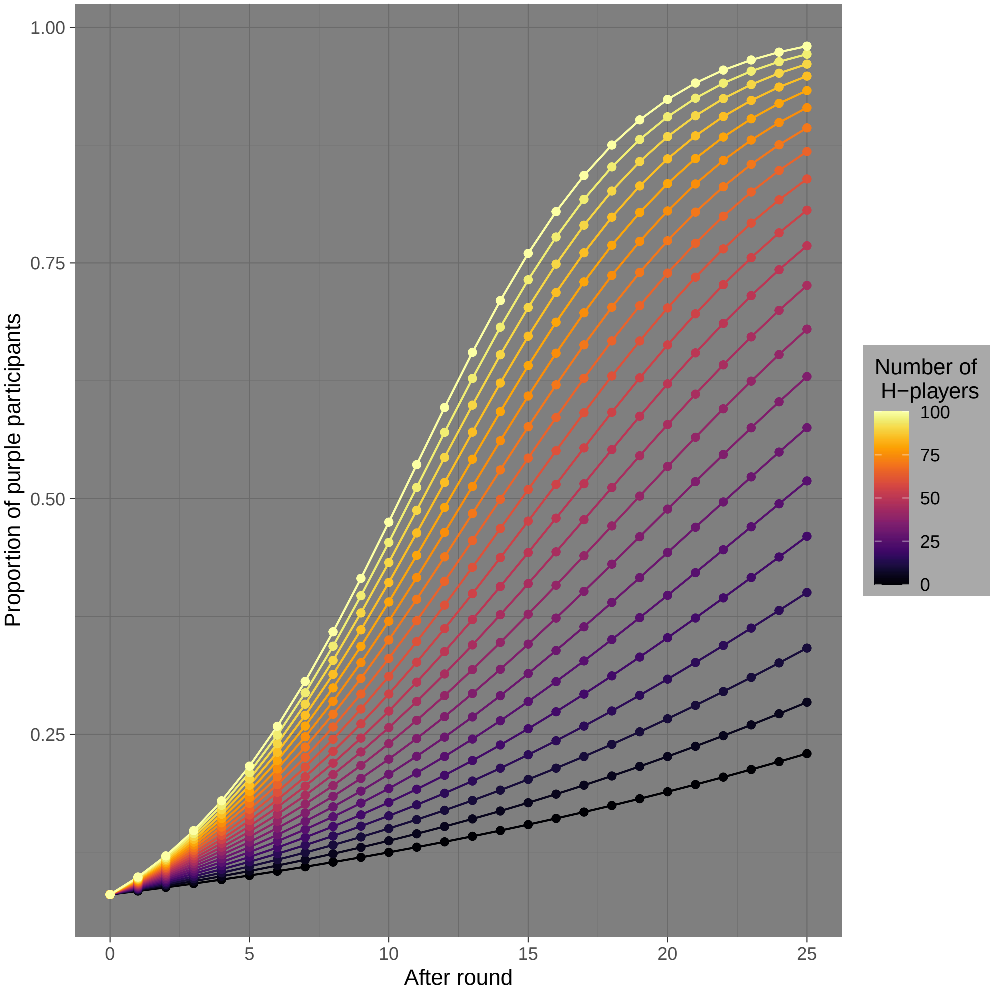

Figure S8

Average proportion of purple participants after each round for each simulated number of H-players: points mark the mean across 1,000,000 games.

S3.2 The social dilemma at the heart of the transmission game

Central to the transmission game is a social dilemma. At first glance, there are two consequences of choosing H-actions over G-actions: there is a gain in scored points and an increase in risk of switching to purple. But it is less clear how these two outcomes trade off against each other. AllG-players will always score $8 \cdot 25 = 200$ points if they stay blue and 0 otherwise. AllH-players will always score $40 \cdot 25 = 1,000$ points if they stay blue and 0 otherwise. Obviously, staying blue will depend both on the player's own actions and other players' actions.

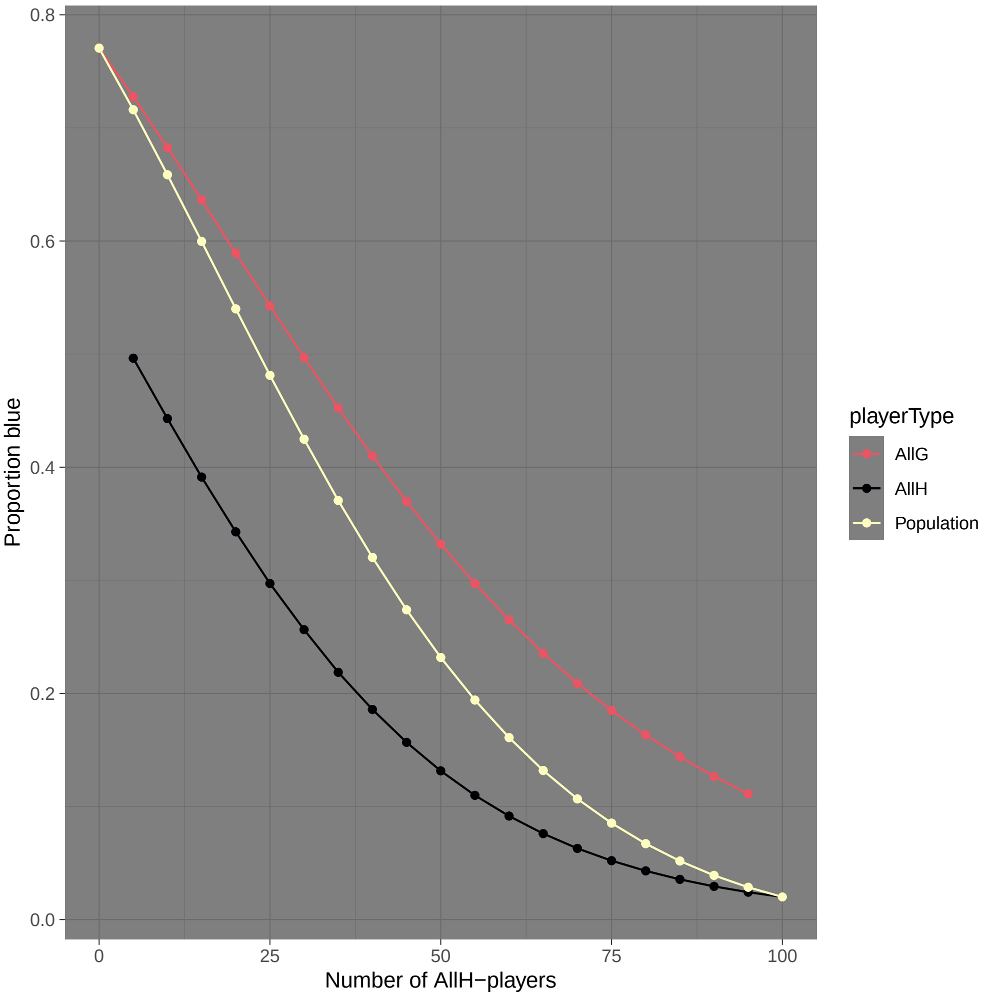

Figure S9

Average rate of staying blue as a function of the number of AllH-players for the full group (population) and the two subgroups of players.

In a first step, we show that the average survival rates depend on the proportion of AllH-players. We summarize average rates and strategy-specific rates in Figure S9. Irrespective of the proportion of AllH-players, AllG-players stay blue at a higher rate than AllH-players. At the same time, with increasing numbers of AllH-players, the rate of blue

players drops for both AllH- and AllG-players. With only a third of all players being AllH-players, the chance to stay blue even for AllG-players is even lower than the chance of AllH-players to stay blue if there are only a few AllH-players. Unsurprisingly, the average rate of staying blue in the group lies between the two subgroups's rate (with the position depending on the relative group size). In spite of this advantage in terms of survival rates, AllG-players score fewer points than AllH-players at a point rate of 5:1. Figure S10 plots average expected payoffs for the player groups.

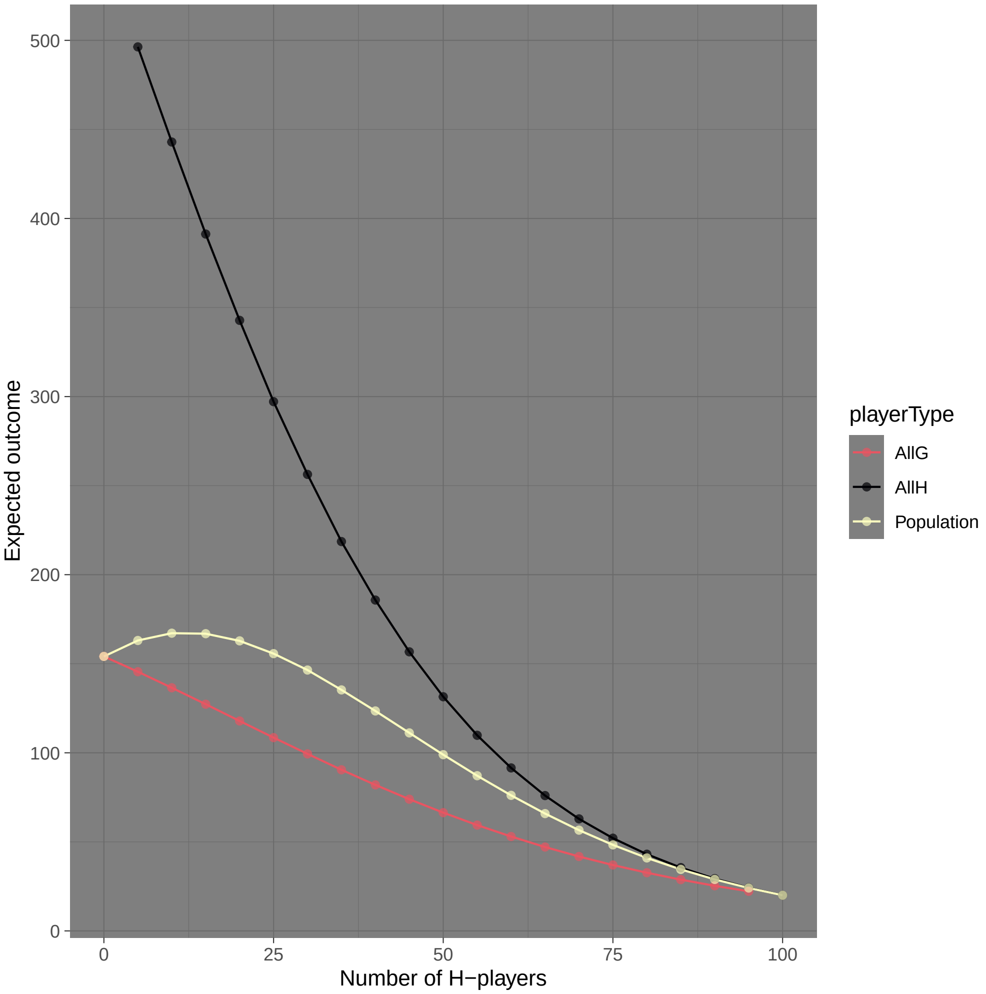

Figure S10

Average expected payoff as a function of the number of AllH-players for the full group (population) and the two subgroups of players.

Irrespective of the proportion of AllH-players, AllH-players have a higher expected payoff than AllG-players. In other words, the AllH-strategy dominates the AllG-strategy in terms of expected payoffs. Thus, there is always an individual incentive not to cooperate. At the same time, if all players choose the AllH-strategy, even the expected outcomes for AllH-players will be lower than the expected outcomes for AllG-players with perfect cooperation.

S3.3 Flattening the curve: A collusive optimum

The analysis of average group outcomes further shows that a group playing a universal AllG-strategy will reach the highest rate of staying blue, but it does not ensure the maximum average expected outcome: a limited number of H-actions increase the expected average value at the cost of a small drop in payout rates. The benefits of these choices would disproportionately favor AllH-players. In **SCR** we explore collective strategies that aim to maximize the average expected value, thus achieving a collusive optimum. We demonstrate that both the distribution of H-choices across players and the spacing of H-actions across rounds impact the final outcomes. In particular, an even distribution of H-actions across players ensures that the accumulated points are not lost due to increased risk of switching colors. Earlier H-actions lead to better outcomes than later H-actions. In early rounds, fewer players are purple and the risky interactions lead to fewer color switches than in later rounds. Thus, the best strategy we identified for a group of players that intends to maximize everyone's expected outcome is to start with a number of H-actions and then switch to G-actions. This strategy is defined by choosing a switching point between H and G. We simulated all possible switching points (including AllG and AllH) and played out 1,000,000 games for each of them. The resulting proportions of purple participants by round are shown in Figure S11.

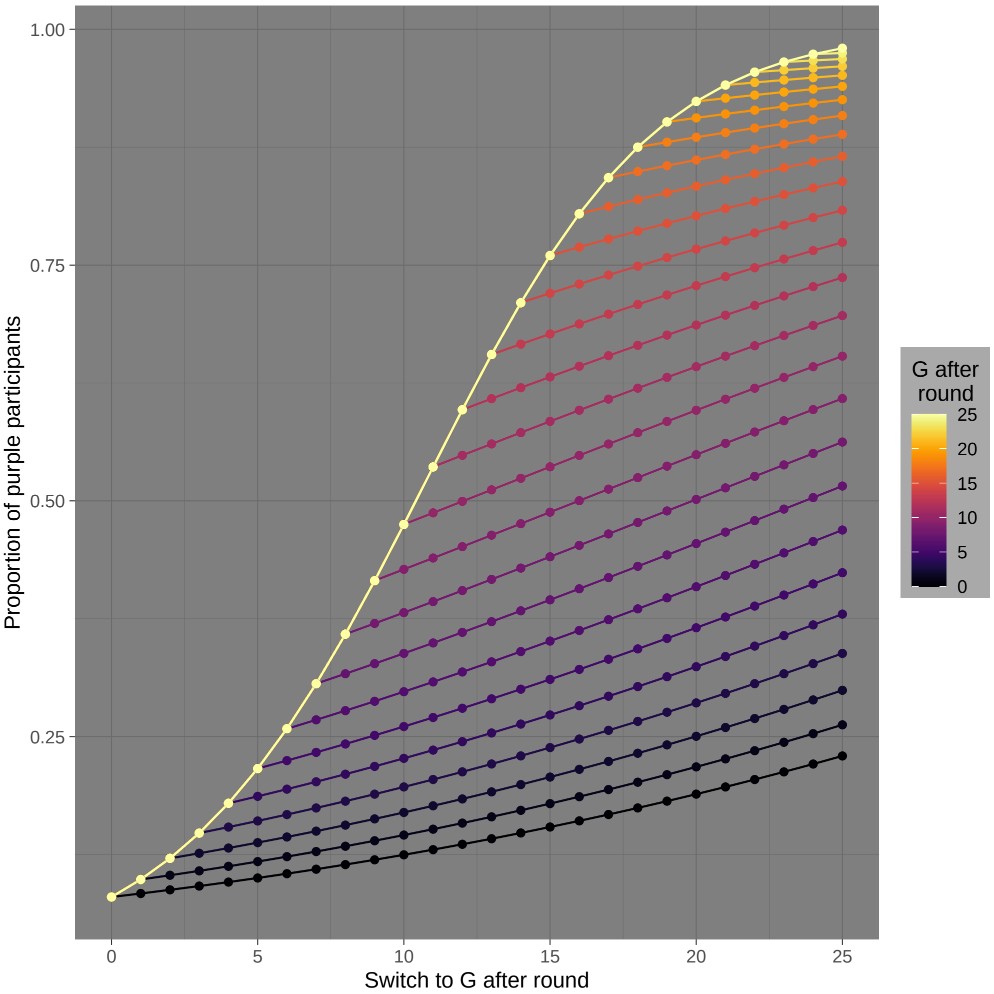

Figure S11

Average proportion of purple participants after each round for each possible collective switching point from H-actions to G-actions: points mark the mean across 1,000,000 games.

It is clear from the setup that all games have the same expected values before the switching point. The upper line corresponds to the AllH-condition with 100% AllH-players, the lowest line to the AllG-condition with 0% AllH-players. One can call the effect of switching "flattening the curve", as the change of strategy leads to a strong reduction in the slope of the curve. As the extreme strategies correspond to the extremes in the previous simulation, the range of possible final outcomes is the same. To determine the quality of switching points, we computed both the final proportion of blue players (the complement of the proportion of purple players after round 25) and the expected value for each player (all players use the same strategy here). This allows us to identify the best options (see Fig. S12).

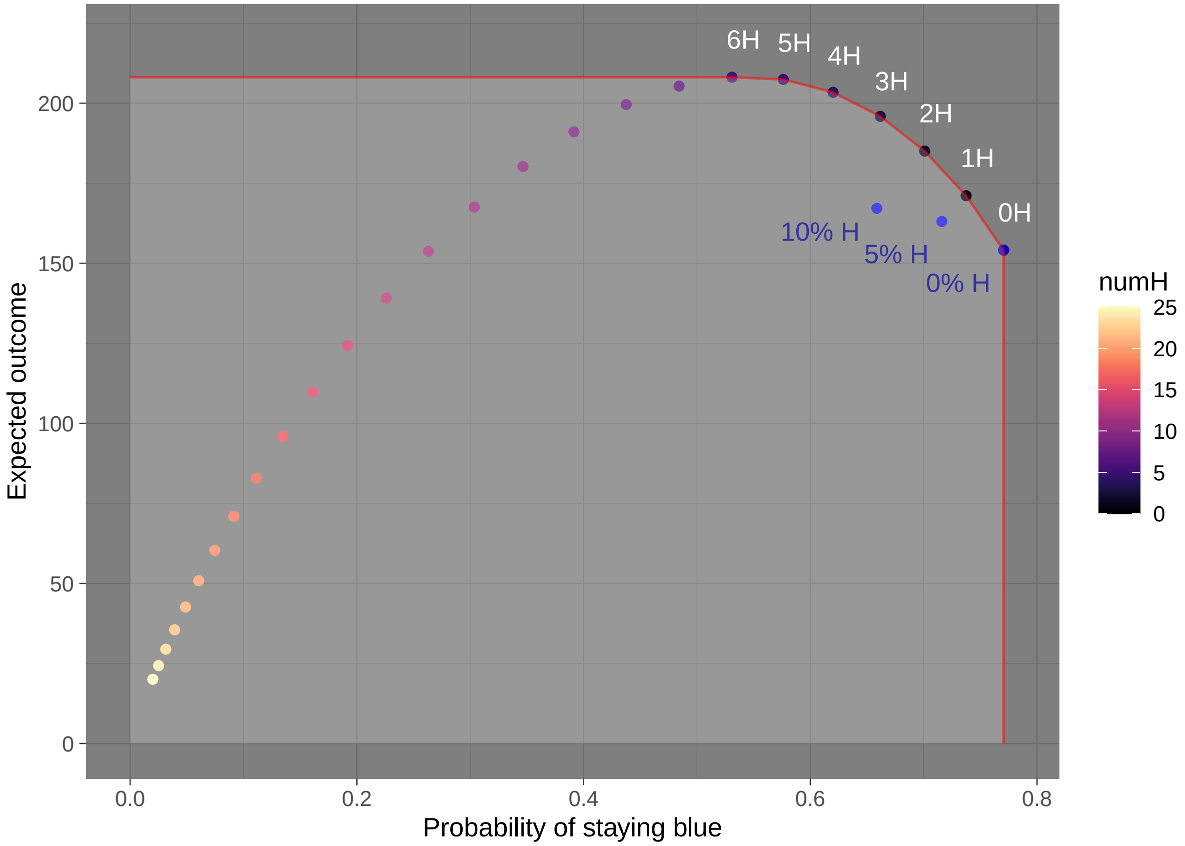

Figure S12

Scatterplot of probability of staying blue (x-axis) and the average expected outcome (y-axis) for all possible switching points: The red line marks the pareto-frontier, pareto-efficient strategies are labelled in white. For comparison, the pareto-efficient strategies for fixed proportions of AllH-players are marked in blue.

Again, we observed the tradeoff between the probability of receiving a bonus payment and the size of the bonus payment. Depending on the degree of risk-aversion, switching points between 0 and 6 are pareto-efficient: there is no other solution that outperforms these solutions on both dimensions. Only a risk-seeking group of players should even consider switching points above 6. The collusive perspective assumes that the group's aim is to achieve the best result for each individual. The highest expected outcome is achieved with a 6H-19G strategy, but the highest probability of being paid is achieved with a 25G-strategy (also corresponding to a fixed percentage of 0% H-players). The best solutions for fixed proportions above 0 achieve lower expected outcomes and lower probabilities of staying blue than these H-G-strategies. An optimal collusive strategy thus requires each individual player to understand that the temporal order of actions matters and to switch from H to G after a small number of rounds. One can assume that this realization requires some cognitive sophistication.

S4 Selected Analytical Results for the Transmission Game

During the review process, there was interest in a more precise analysis of game results. We present the analytical tools we developed in the Extended Supplementary Materials (Section E4 and Section E5, `https://doi.org/10.7910/DVN/ILDEMO`). These tools offer opportunities for revisiting some previous results. Here, we compare the new analytical results with previous simulation estimates. In the Extended Supplementary Materials, we also repose the question of equilibria and "optimal" behavior.

Showing that simulation results and analytical results align is supportive evidence for claiming that our simulations are correct implementations of the described game and our analytical tools are indeed able to compute their expected results (if one excludes the possibility of a common error in both).

S4.1 Revisiting the flattening of the curve

To demonstrate the equivalence, we choose the earlier results on flattening the curve. In this simulation, we simulated all possible strategies that start with H-actions and from some round on switch to G-actions for the remainder of the game. Depending on the switching point, we found different shapes of infection curves with large differences in final states. Figure S13 directly compares simulation results and analytical results. The maximum absolute difference across all plotted values is smaller than 0.00018 (or 0.018%), with no systematic direction of deviation.

S4.2 Precise estimation of roundwise results

The results generated by the analysis tools are of course richer than the expected end points for infection numbers. For each round, the tool generates the precise probability distribution for population states. For a selected strategy (an HG-strategy with six initial H-actions), the progression of population state distributions across rounds is shown in its entirety in Figure S14.

The mean of each distribution corresponds to the points in Figure S13 that are located on the seventh line from the bottom (the bottom-most line corresponds to 0 H-choices). Naturally, the longer the game lasts the larger the variance of game states will be (at least for infection rates below 50%). Extremely high values are still possible (starting after round 4, 100 infected players are technically possible), but not probable. See the Extended Supplementary Materials for further applications of the analysis tools (Section E6, `https://doi.org/10.7910/DVN/ILDEMO`).

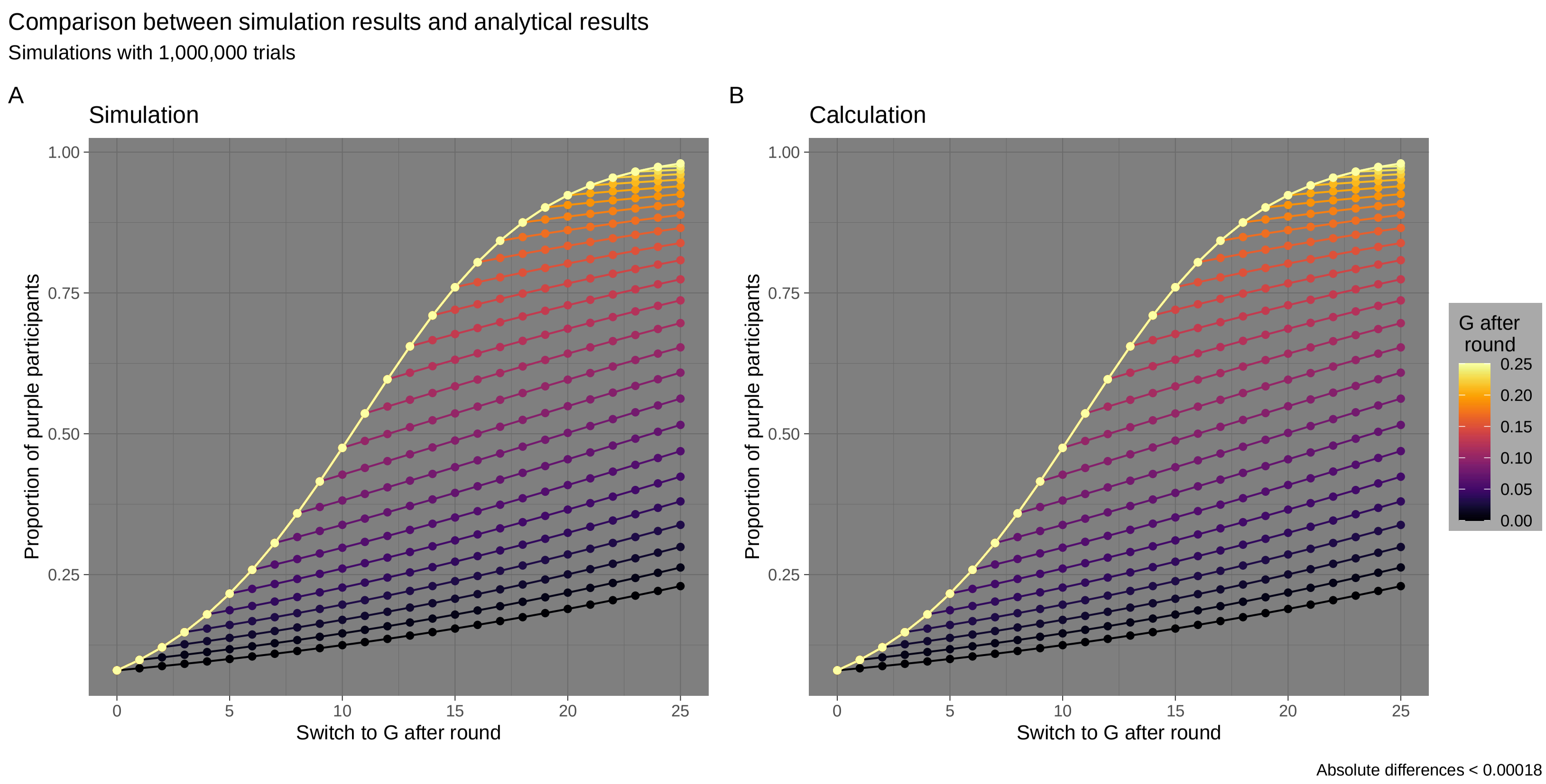

Figure S13

Average proportion of purple participants after each round for each possible collective switching point from H-actions to G-actions: (A) points mark the mean across 1,000,000 simulated games (B) points mark the expected values based on analytical results.

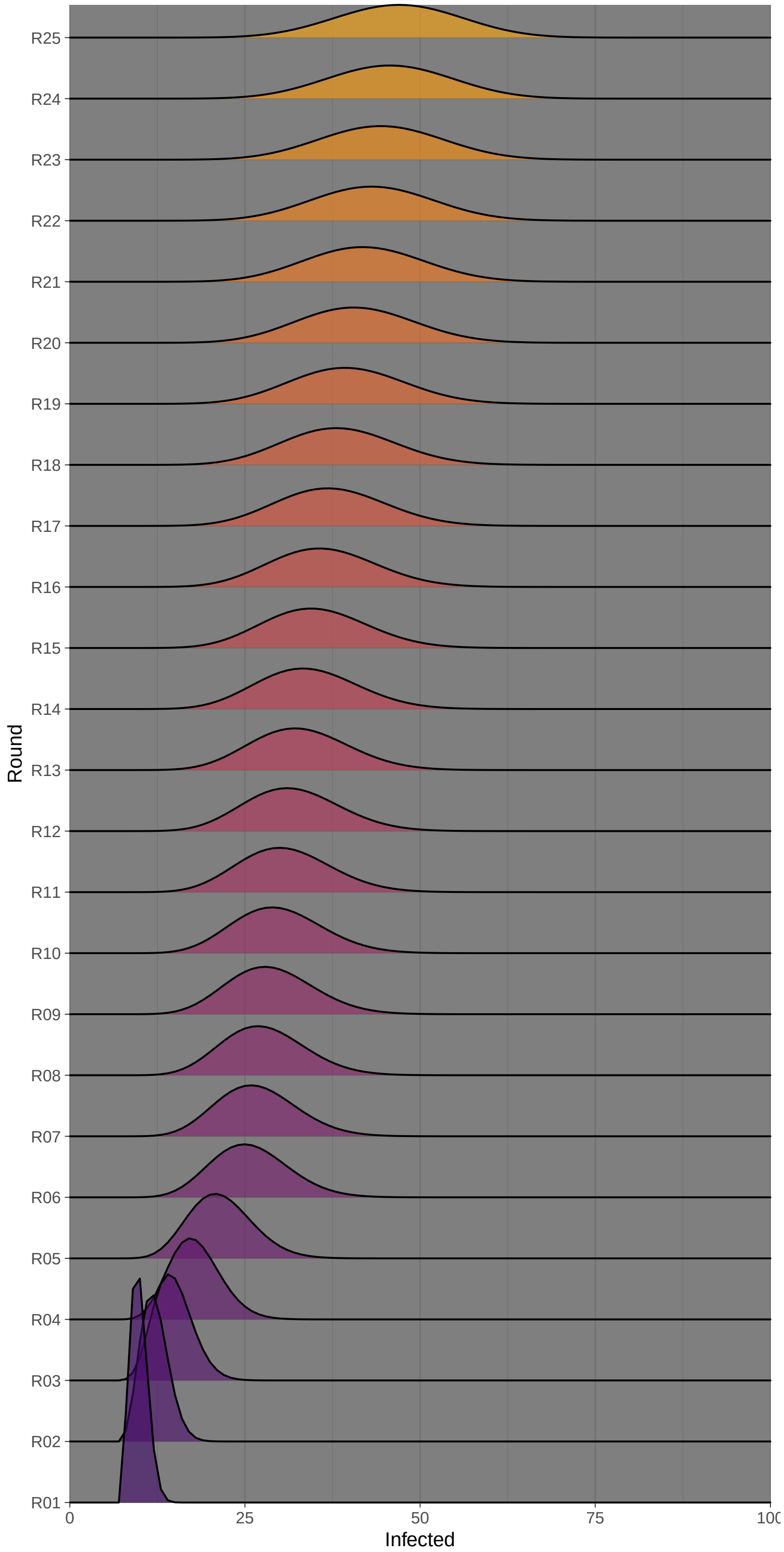

Figure S14

Derived roundwise distributions of computed population states for a HG-strategy with 6 H-actions in the first six rounds and 19 G-actions.

S5 Study 1: Selected Materials

Note: The full set of materials is found in document ***SM1*** *(Study Materials Study 1, https://doi.org/10.7910/DVN/RHR2A5.*

S5.1 Transmission game instructions

Here, we omit the comprehension checks that had to be passed to proceed from one page to the next (16 checks in total, see **SM1**).

S5.1.1 Page 1/5

[*See Figure S15.*]

In the next part, you will play a game with 99 other participants (there are 100 players in this game, including you).
The other participants might not play the game at the same time as you do, so you will not have to wait for others.
Rest assured that the other players are other participants on Prolific. We never deceive participants in our instructions or invent participants who do not exist.
We will determine the results of the game, after we have collected the responses of all players.

Next, we will explain the rules of the game.

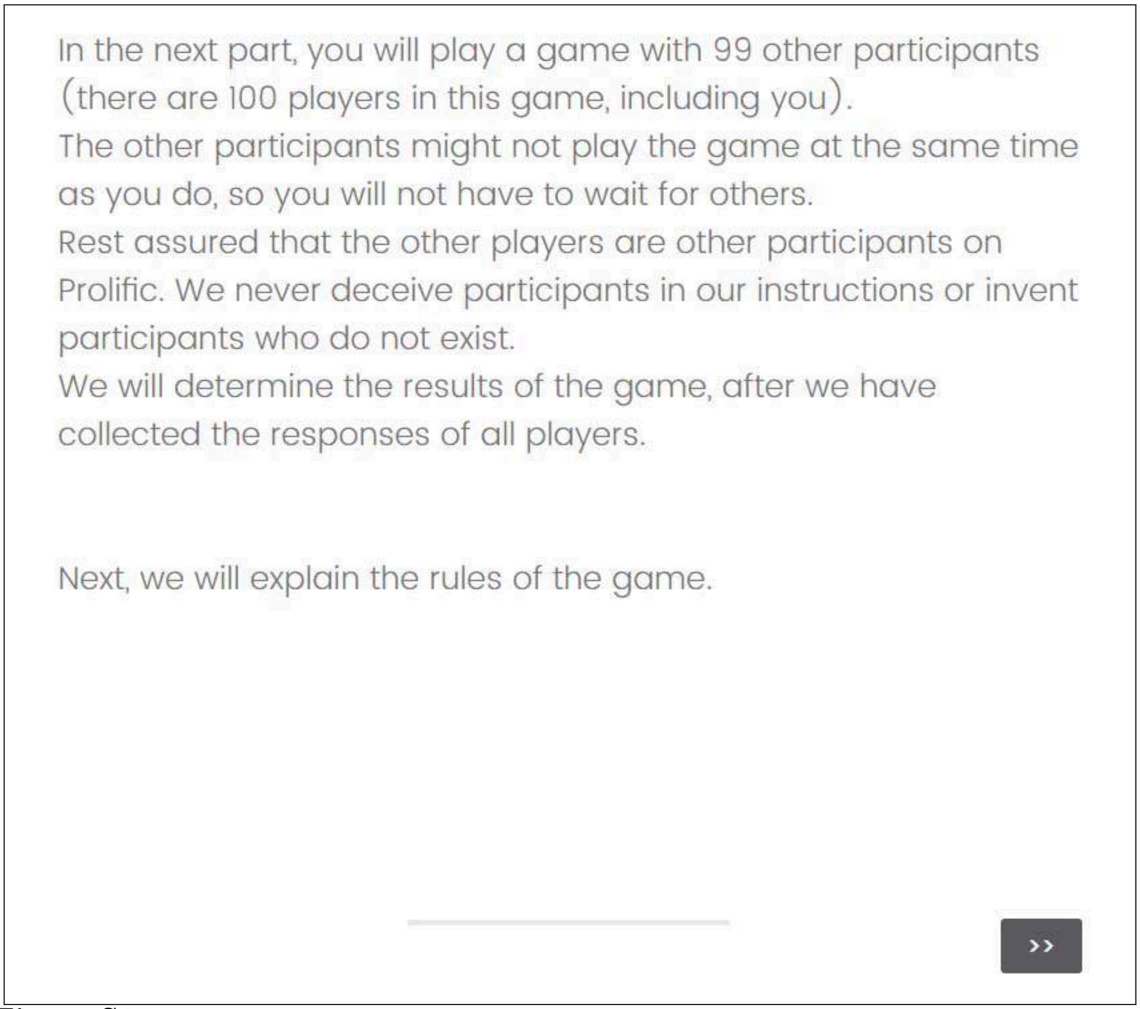
In the next part, you will play a game with 99 other participants (there are 100 players in this game, including you).
The other participants might not play the game at the same time as you do, so you will not have to wait for others.
Rest assured that the other players are other participants on Prolific. We never deceive participants in our instructions or invent participants who do not exist.
We will determine the results of the game, after we have collected the responses of all players.

Next, we will explain the rules of the game.

››

Figure S15
Game Instructions Page 1

S5.1.2 Page 2/5

[*See Figure S16.*]

The game lasts 25 rounds.
In each round you will make one decision. Your decisions will affect your own bonus payment and can affect other players' bonus payments.

Every player has a color, either blue or purple.
You and every other player all start as blue players.

Some players may change from blue to purple in the course of the game (we explain below how this can happen).
Once a player has changed to purple, they stay purple for the remainder of the game.

It is not possible to change back to blue.

Like all the other players, you will only know your color at the beginning (blue).
Once the game has started, and throughout the game, nobody will know their own color, nor will they know any other player's color.

In the course of the game, you will earn points.

At the end of the game, blue players will receive a bonus payment based on the number of points they have earned across all rounds.
Each point is worth a fixed amount of bonus money for a blue player.

In contrast, purple players will NOT receive a bonus payment for this game. Their points are not converted into money at the end of the game.

The game lasts 25 rounds.
In each round you will make one decision. Your decisions will affect your own bonus payment and can affect other players' bonus payments.

Every player has a color, either blue or purple.
You and every other player all start as blue players.

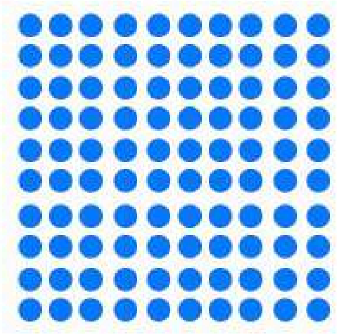

Some players may change from blue to purple in the course of the game (we explain below how this can happen).
Once a player has changed to purple, they stay purple for the remainder of the game.
It is not possible to change back to blue.

Like all the other players, you will only know your color at the beginning (blue).
Once the game has started, and throughout the game, nobody will know their own color, nor will they know any other player's color.

In the course of the game, you will earn points.

At the end of the game, blue players will receive a bonus payment based on the number of points they have earned across all rounds.
Each point is worth a fixed amount of bonus money for a blue player.

In contrast, purple players will NOT receive a bonus payment for this game. Their points are not converted into money at the end of the game.

Figure S16

Game Instructions Page 2

S5.1.3 Page 3/5

[*See Figure S17.*]

At the start of the game, just before the first round, eight randomly chosen players will change from blue to purple.
No player will know whether they have changed color or not: After the start of the game, no player knows their color.

In each round of the game, each player will be paired with one of the other 99 players (there is an equal chance to be paired with any one of them).
Both will decide on one action: either G or H. These decisions will affect your bonus payment.

A player who chooses action G will receive 8 points.
A player who chooses action H will receive 40 points.

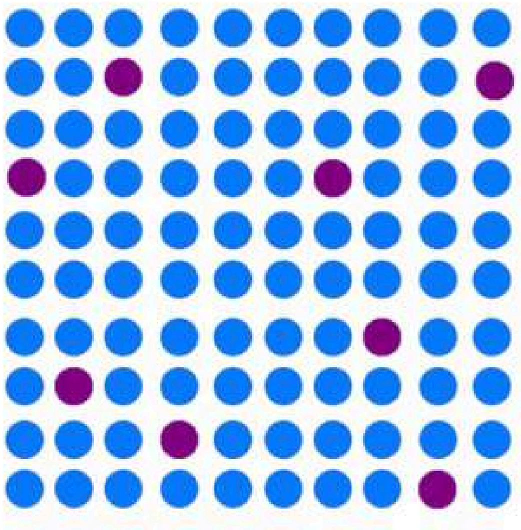

At the start of the game, just before the first round, eight randomly chosen players will change from blue to purple. No player will know whether they have changed color or not: After the start of the game, no player knows their color.

In each round of the game, each player will be paired with one of the other 99 players (there is an equal chance to be paired with any one of them).
Both will decide on one action: either G or H. These decisions will affect your bonus payment.

A player who chooses action **G** will receive **8** points.
A player who chooses action **H** will receive **40** points.

Figure S17
Game Instructions Page 3

S5.1.4 Page 4/5

[*See Figure S18.*]

If two blue players or two purple players are paired, neither player will change color in this round.

What happens if a blue player is paired with a purple player?
In this case, the blue player may change into a purple player.
The probability of this change depends on the actions that both players choose in this round.

If both choose action G (8 points), there is a 5% chance that the blue player changes into a purple player in that round.
This means, 1 in 20 blue players would change on average in this situation, 19 of 20 would stay blue.

If one of the two players chooses action H (40 points) instead, the chance is increased (by 10%) to 15%.
This means, 3 in 20 blue players would change on average in this situation, 17 of 20 would stay blue.

If both players choose action H, the chance is increased (by another 10%) to 25%.
This means, 5 in 20 blue players would change on average in this situation, 15 of 20 would stay blue.

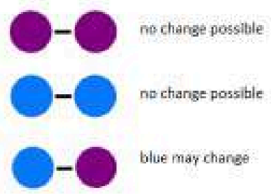

If two blue players or two purple players are paired, neither player will change color in this round.

What happens if a blue player is paired with a purple player?
In this case, the blue player may change into a purple player.
The probability of this change depends on the actions that both players choose in this round.

Chance of color change to purple

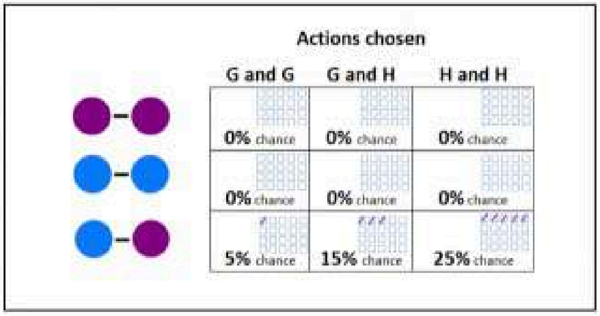

If both choose action G (8 points), there is a 5% chance that the blue player changes into a purple player in that round.
This means, 1 in 20 blue players would change on average in this situation, 19 of 20 would stay blue.

If one of the two players chooses action H (40 points) instead, the chance is increased (by 10%) to 15%.
This means, 3 in 20 blue players would change on average in this situation, 17 of 20 would stay blue.

If both players choose action H, the chance is increased (by another 10%) to 25%.
This means, 5 in 20 blue players would change on average in this situation, 15 of 20 would stay blue.

Figure S18
Game Instructions Page 4

S5.1.5 Page 5/5

[*See Figure S19.*]

As you know, you can receive 8 points or 40 points in each of the 25 rounds. How much would you earn per point if you are a blue player at the end of the game?

Points are converted into bonus money at an exchange rate of:
10 points equal 5p.

So, 100 points are worth 50p and 200 points are worth £1 of possible bonus payment.
Each choice of action G is potentially worth 4p in the end, each choice of action H is potentially worth 20p.

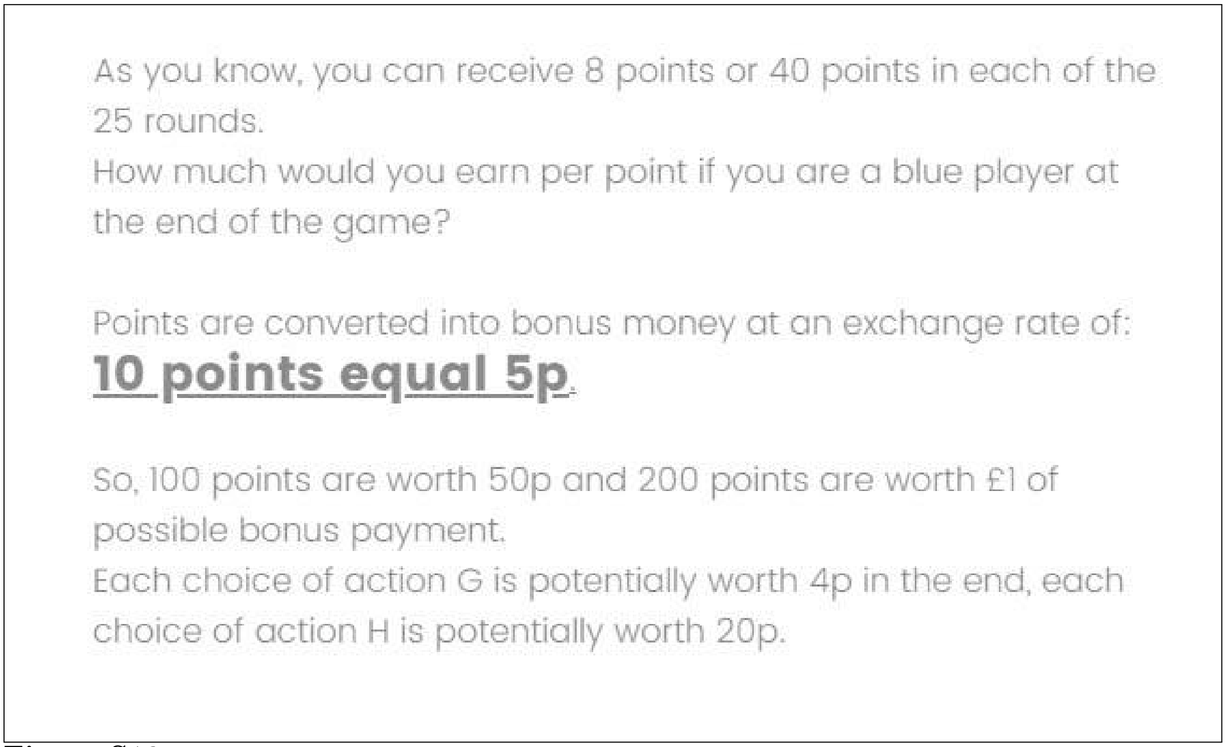

Figure S19
Game Instructions Page 5

S5.2 Transmission Game

S5.2.1 Introduction

[*See Figure S20 and Figure S21.*]

You will now play the game. Note that every other player in your game will have the same information as you have. They will also have seen the same instructions as you have.

This is a brief summary of the rules:

1. **At the start of the game** all 100 players (yourself included) are blue.
2. **Before the first round only**, eight randomly selected players change into purple players.
3. **During each round**:
   - Players are paired at random.
   - Each player chooses an action and receives points (8 points for G, 40 points for H).
   - A blue player that is paired with a purple player may change into a purple player (with a probability between 5% and 25%, see the figure below).
   - A blue player that is paired with a blue player never changes color, no matter which actions are chosen.
4. **After 25 rounds, at the end of the game**, the payoff across all rounds for blue players will be converted into bonus money. Purple players will not receive bonus money for this task.

You will not have to wait for the other players (they may play the game before or after you).
We will collect your decisions and the decisions of 99 other players, then play out the game once to determine the results.
Afterwards, we will inform you about your color at the end of the game in a message when you receive your bonus payment for the entire survey.

You will now play the game. Note that every other player in your game will have the same information as you have. They will also have seen the same instructions as you have.

This is a brief summary of the rules:

1) **At the start of the game** all 100 players (yourself included) are blue.
2) **Before the first round only**, eight randomly selected players change into purple players.
3) **During each round**:

- Players are paired at random.
- Each player chooses an action and receives points (8 points for G, 40 points for H).
- A blue player that is paired with a purple player may change into a purple player (with a probability between 5% and 25%, see the figure below).
- A blue player that is paired with a blue player never changes color, no matter which actions are chosen.

4) **After 25 rounds, at the end of the game**, the payoff across all rounds for blue players will be converted into bonus money. Purple players will not receive bonus money for this task.

You will not have to wait for the other players (they may play the game before or after you).
We will collect your decisions and the decisions of 99 other players, then play out the game once to determine the results. Afterwards, we will inform you about your color at the end of the game in a message when you receive your bonus payment for the entire survey.

Figure S20
Game Introduction

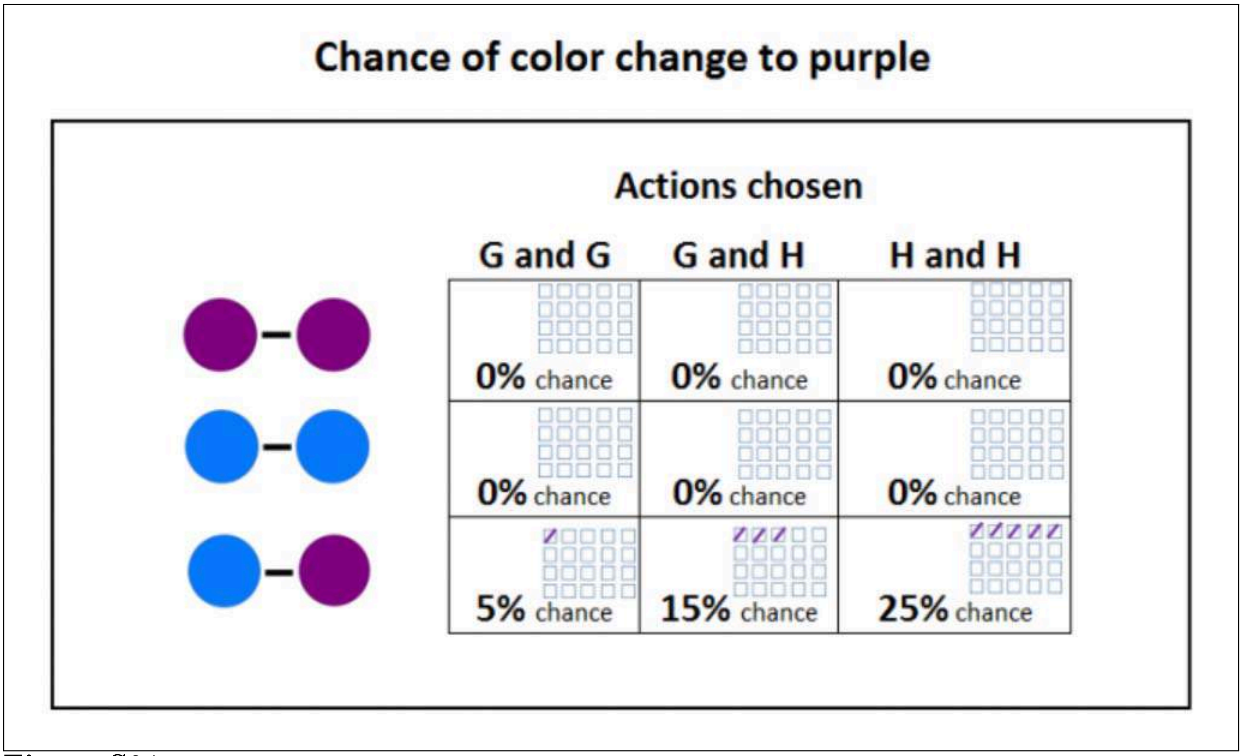

Figure S21

Game matrix (presented below game introduction)

S5.2.2 Rounds

[*See Figure S22 and Figure S23.*]

Round [*current round*]/25
Points gained so far: [*current number of points*] (+ [*points gained in previous round*])
Possible bonus: £[*current bonus*] (+£[*bonus amount gained in previous round*])
Points will be converted into bonus money after the final round (for blue players only).

Which of the two actions do you choose?

- Action G: I receive 8 points. (8)
- Action H: I receive 40 points. (40)

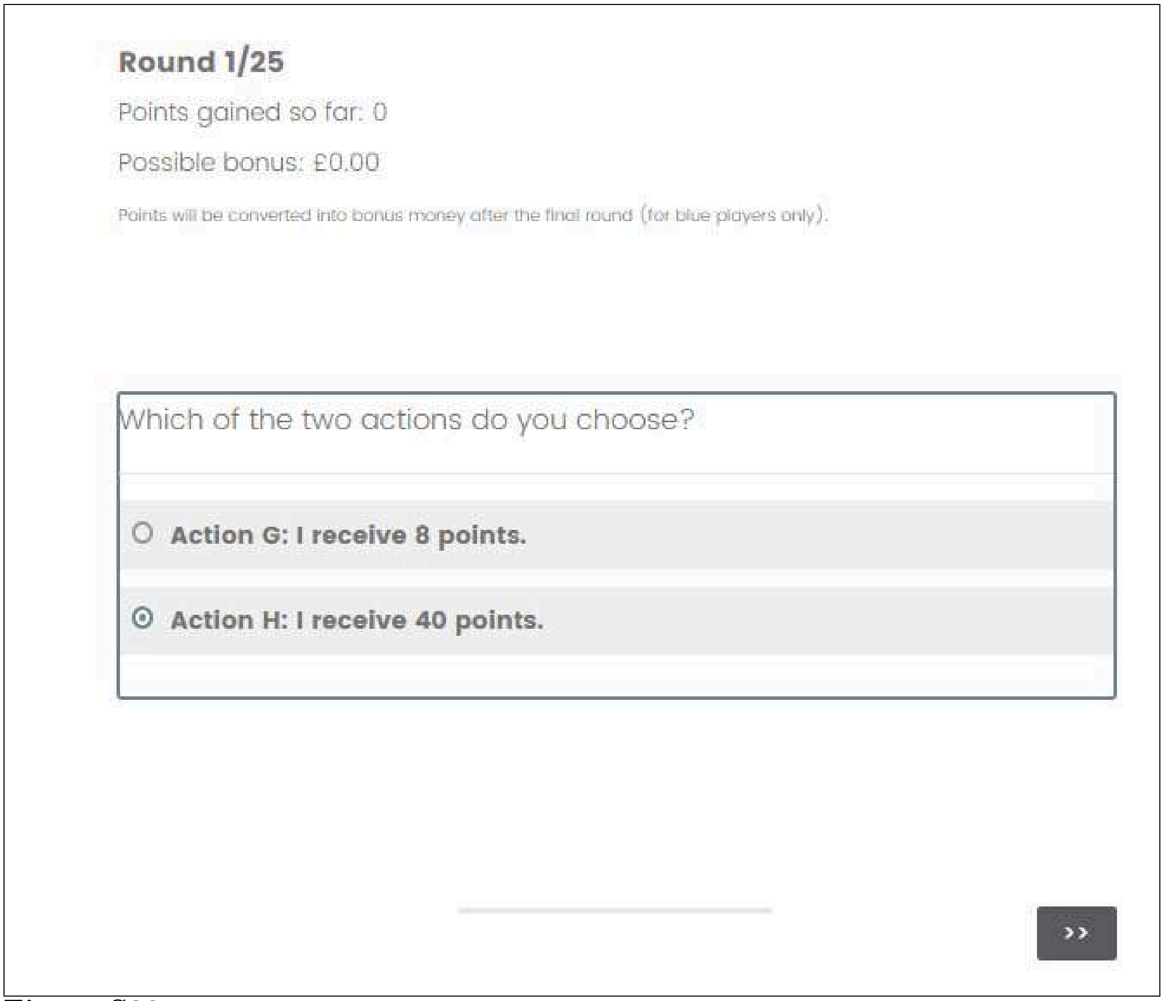

Figure S22
Game round 1 (example)

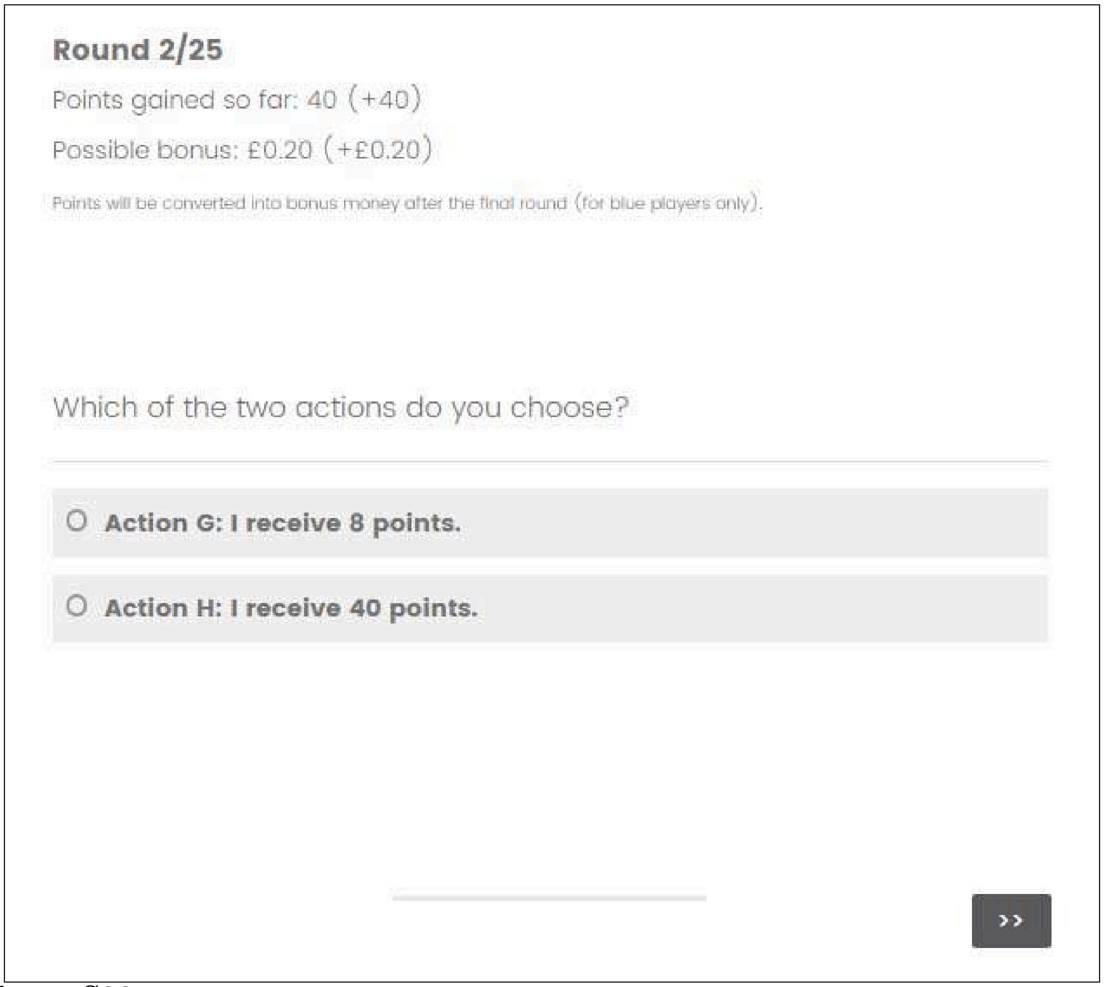

Figure S23
Game round 2 (example)

S5.2.3 Final results

[*See Figure S24.*]

End of game
Points gained: [*final number of points*]
Possible bonus: £[*final bonus amount*]
Points will be converted into bonus money (for blue players only).

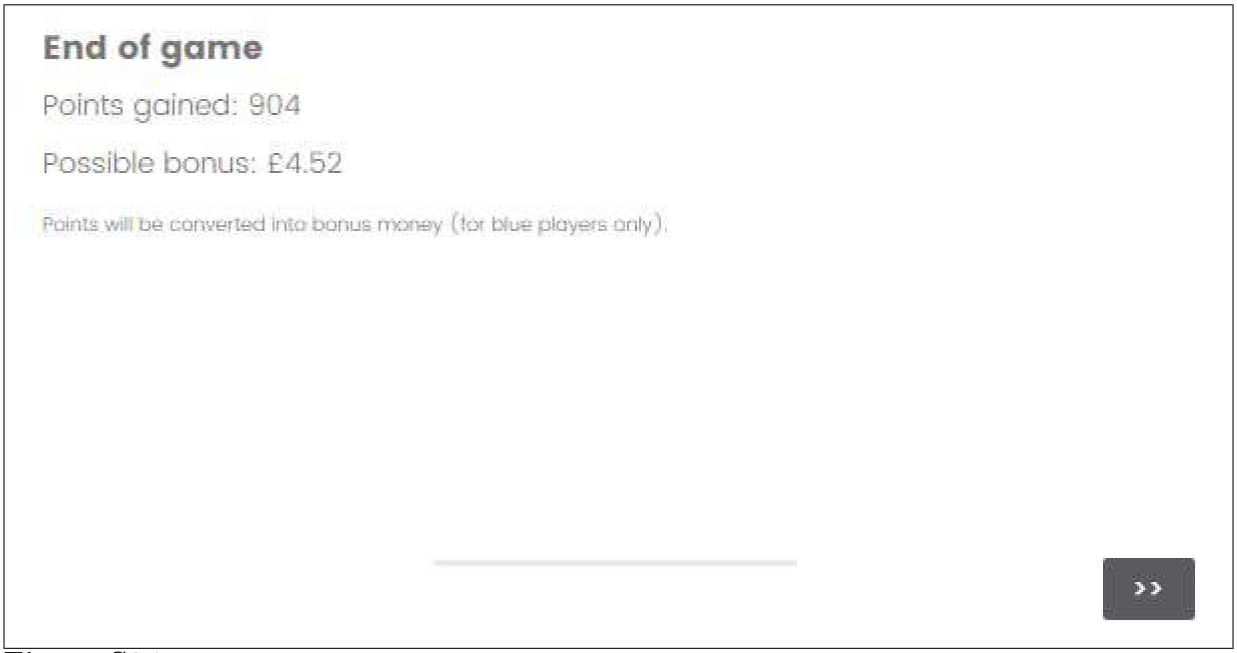

Figure S24

End of game (example)

S6 Study 1: Selected Results

Note: The full set of results is found in document ***SA1*** *(Supporting Analyses Study 1). Variables and measures are described in document* ***VM1*** *(both documents are at* `https://doi.org/10.7910/DVN/RHR2A5`*).*

S6.1 Behavior in the transmission game

A sensible statistic for summarizing behavior in the transmission game is the number of risky choices made throughout the game. The number of risky choices, n_r, is linearly related to the final score after round 25 ($S_{25} = 8 \cdot 25 + 32 \cdot n_r$). Figure S25 shows the distribution of final scores across the 100 participants, demonstrating considerable variance in the sample.

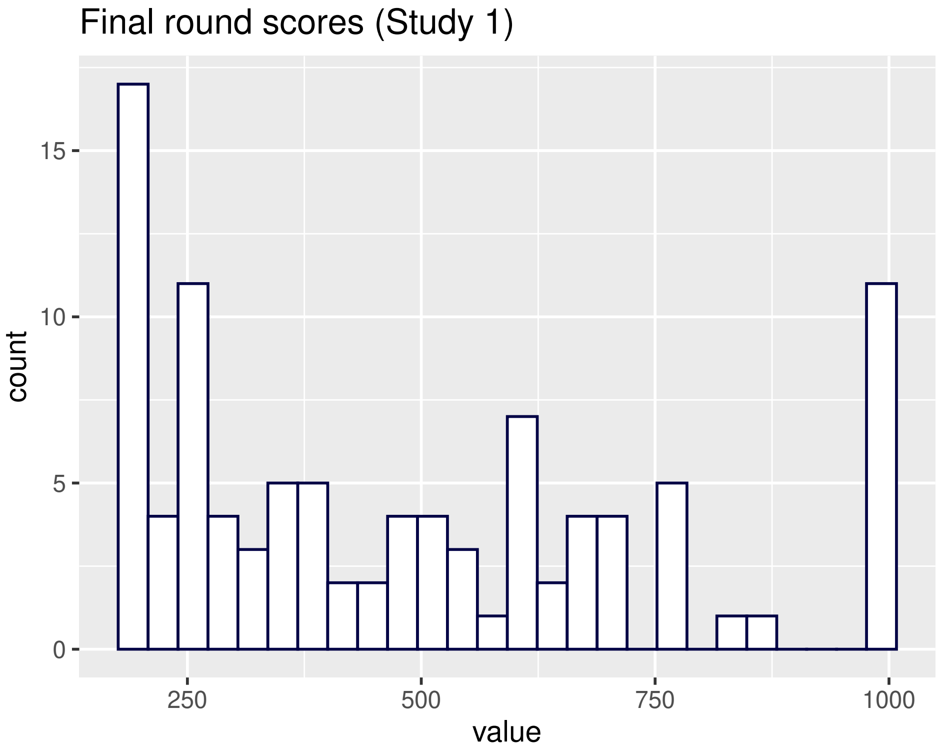

Figure S25
Histogram of scores after round 25 (n=100).

Figure S26 shows the proportion of H-choices in each round of the game. The majority of participants chooses action H in the first round, and this percentage decreases over the course of the game, yet stays above 25% for each single round.

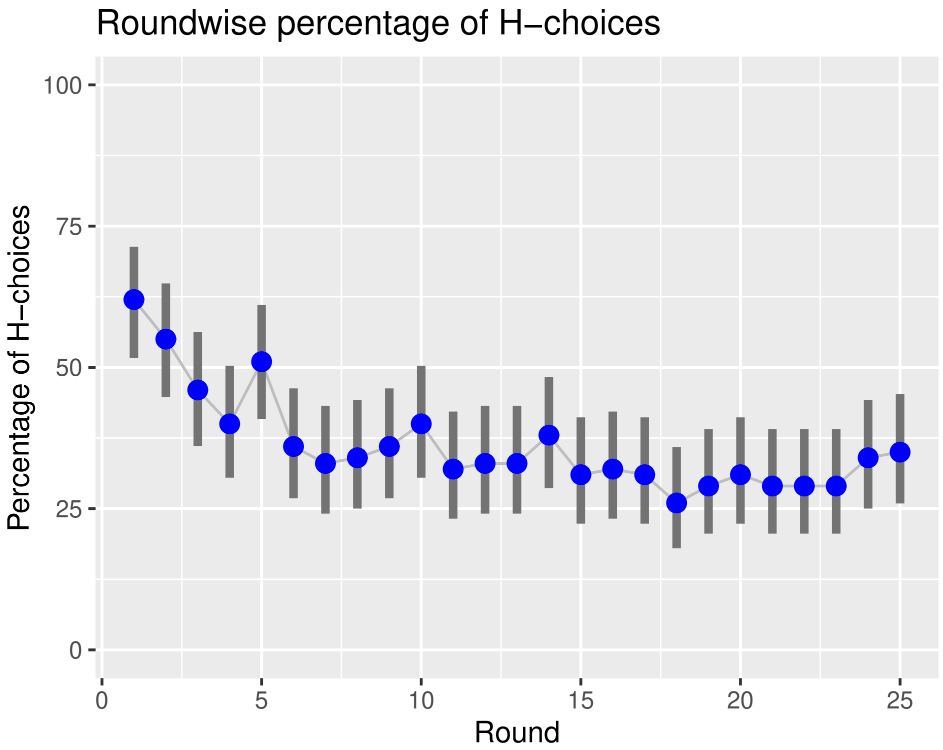

Figure S26

Proportion and 95% CI of proportion of H-choices for each round of the game.

S6.2 Postquestionnaire results

S6.2.1 Game motivation

The general postquestionnaire asked for participants' motivation in the game. The postquestionnaire included the following questions (measured on a scale from 1–5, the name at the start of each line is only used in the figure below):

- MaxBonus: I wanted to make as much bonus money as possible.
- MoreBonus: I wanted to make more bonus money than other players.
- Responsible: I felt responsible for other players.
- MakeSwitch: I wanted to make other players switch color.
- DoNotCare: I did not care at all what happened in this game.

The distribution of postquestionnaire responses and their correlation with the game score are summarized in Figure S27. Most participants were focused on achieving the maximum bonus possible, but less so on achieving more than other participants. There was substantial variance in the feeling of responsibility for other participants, but few participants wanted to make other participants switch color or indicated that they did not care about the game result.

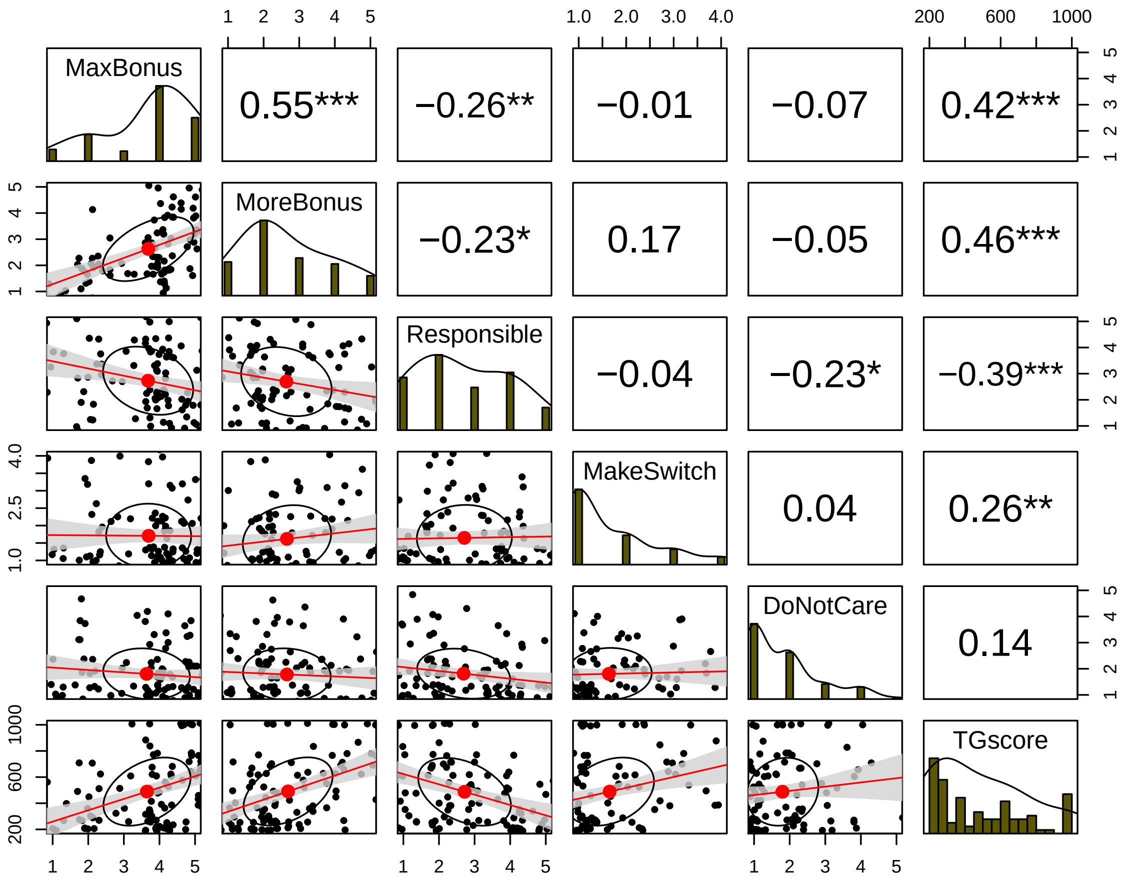

Figure S27

Histograms of postquestionnaire-responses and game score with scatterplots and correlation coefficients.

The indicated goals were clearly consistent with the observed game behavior. Participants who indicated that they wanted the maximum bonus and those who wanted to have more than others tended to have higher game scores. In contrast, those feeling responsible for others chose the H-action less often. A somewhat weaker relationship is observed between the intention to make others switch and H-actions, and only a weak correlation is seen between disinterest in game results and the game score.

S6.2.2 Strategy descriptions

The distribution of responses (see Figure S25) shows that a substantial number of participants consistently chose the same strategy throughout the game, *AllG* or *AllH*. Asked about their strategy when playing the game, a typical response for an *AllG*-player was "I stuck with the safest option always, for fear of turning purple". The focus for *AllH*-players was different, for example: "I always chose H because it was the highest potential payoff".

Players choosing each action (G and H) at least once fell into two distinct categories. Some participants indicated that they wanted to switch between options to achieve a balanced outcome. In deciding when to switch they did not offer clear guiding principles (*Switch*-players). One typical explanation was: "I switched between picking H and G to even out my playing field, I wanted the most bonus money but also didnt want to end up being purple so I took risks when needed". A more sophisticated strategy involved choosing a single switching point, after starting the game with one or more H-actions (*HG*-players). We show an independent justification for this strategy in our simulations, but several participants clearly expressed their intuition: "I picked the risky actions at the start of the game in order to built up more points as there is a lower amount of purple players but as the game continue it was safer to pick the less risky options (8 pt as opposed to 40 pt) since its likely that the number of purple players would increase as well. Due to the risk of turning purple, it was more important to me to aim for lower pts than to risk losing all pts."

More of these explanations are compiled in **VM1** (`https://doi.org/10.7910/DVN/RHR2A5`). In general, strategies seemed to correspond well with the objective game features, validating that our instructions succeeded in conveying the game rules. See document **VM1** for a selection of responses regarding associations with the transmission game in open-format answers. The majority of participants (about 80%) did not associate the game with aspects of COVID-19. Some participants offered analogies in the intended direction: "COVID-19 and Social Distancing. In a blue and purple pairing, if both choose G there is less likely to be a spread of purple. If one chooses G and the other H, then theres still a chance of spread but less of it. If both choose H, the risk is highest."

S6.2.3 Estimates of other participants' behavior

Participants estimated the percentage/number of purple players for six different rounds (1, 5, 10, 15, 20, 25). Figure S28 summarizes their estimates: Median responses predict a close to linear increase from the initial purple proportion (8%) to over 70% purple players. The outliers indicate players who most likely gave estimates for G-actions.

Median estimates for the number of players choosing the H-action in each round showed a clearly decreasing trend starting at 80% and ending at about 30% (see Fig. S29). These numbers tended to over-estimate the actually observed round proportions.

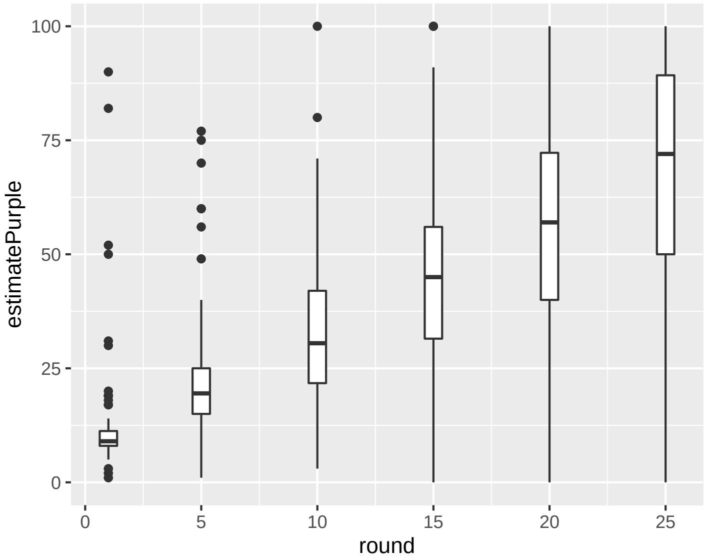

Figure S28

Boxplots of estimates of the number of purple players after rounds 1, 5, 10, 15, 20, and 25.

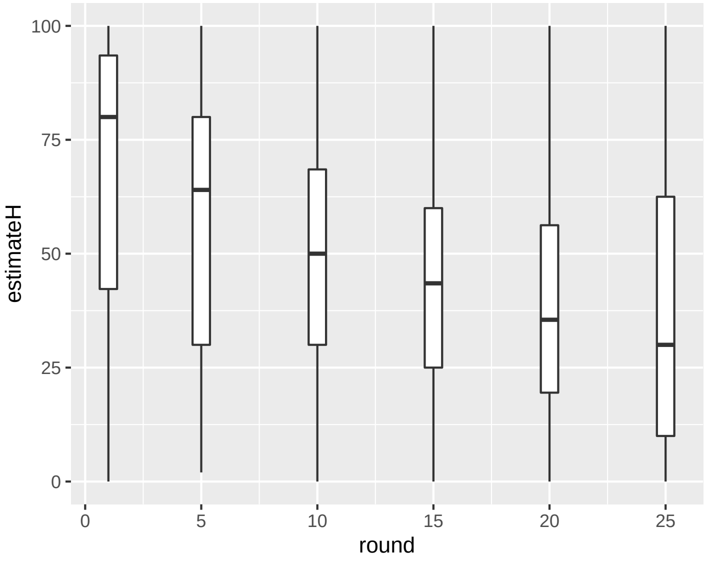

Figure S29

Boxplots of estimates of the number of H-actions in rounds 1, 5, 10, 15, 20, and 25.

S6.3 Predictors of game results

The goal of Study 1 was to identify predictors of game results using a wider array of plausible scales and variables. Thus, we advise caution in interpreting these findings without replication (as attempted in Study 2). The range of considered variables is exhaustively presented in **SM1**, **VM1**, and **SA1** (all at `https://doi.org/10.7910/DVN/RHR2A5`). Here, we reduce the complexity by presenting only relationships with variables that we identified as promising in these analyses.

These variables, and their relationship with game results, are presented in Figure S30.

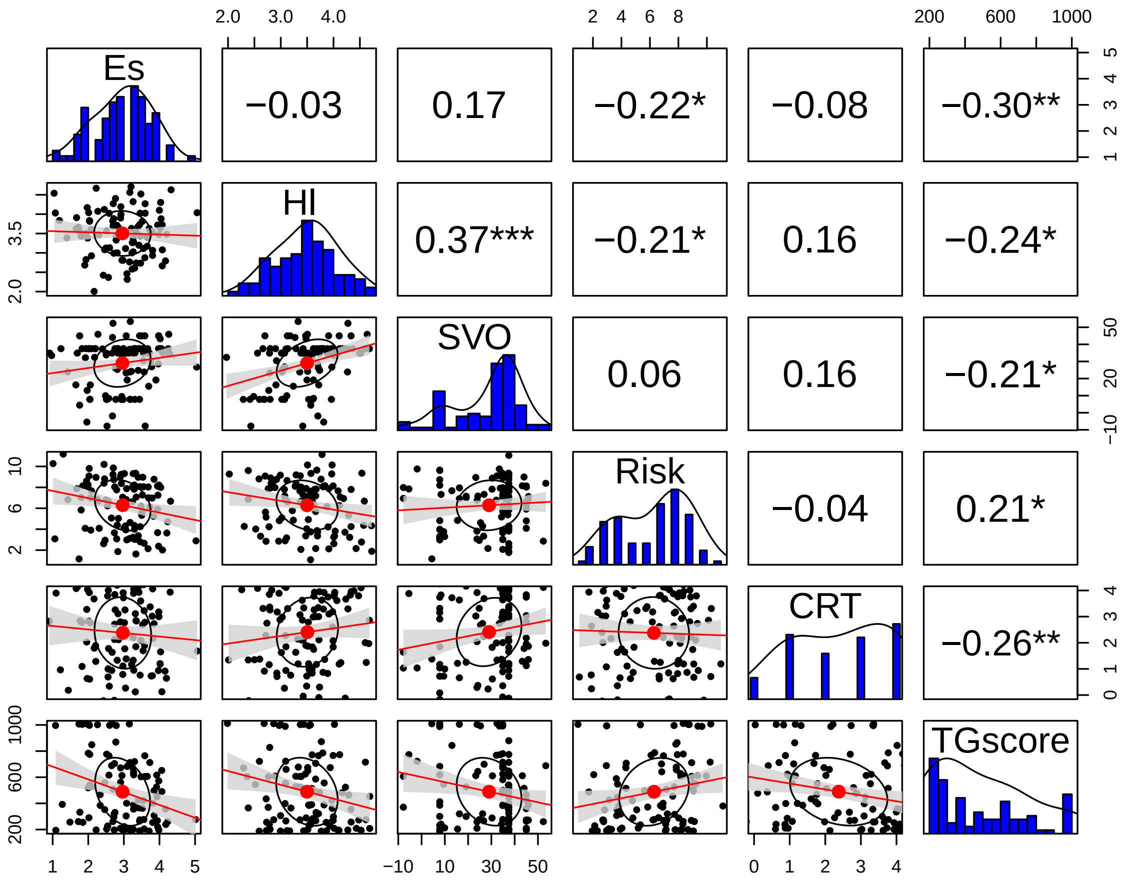

Figure S30

Predictors of game behavior: Histograms, correlations and scatterplots for variables predicting the game score (TGScore), which are, in order, the brief HEXACO-Emotionality scale (Es), the longer HEXACO-Honesty/Humility scale (Hl), the angle computed for social value orientation (SVO, higher values indicate altruistic choices), the general risk item (Risk), and the score in the cognitive reflection test (CRT).

S7 Study 2: Selected Materials

Note: The full set of materials is found in document ***SM2*** *(Study Materials Study 2, see Section S1).*

S7.1 Transmission Game: Condition-specific instructions

S7.1.1 Instructions: Injunctive norms condition

[*See Figure S31.*]

We would like to highlight some consequences of your choices:

You will have noticed that you can obtain a **personal benefit** by choosing action H (=40 points) over action G (=8 points).
But be aware that this benefit in points comes at a **potential cost paid by others, and even by yourself**.
If you are a purple player and choose action H, there is a higher chance that a blue player you are paired with will turn purple (thereby losing their bonus money for the game).
If you are a blue player paired with a purple player and you choose action H, then you will have a higher chance of turning purple yourself (thereby losing your bonus money for the game).

All points scored across the rounds are worth nothing to a purple player.

Choosing action H may therefore endanger your own bonus money and the bonus money of other participants.
Choosing action G protects others and yourself from changing into purple players and losing bonus money.

In brief:
Choose action G to protect your and other players' bonus money.

S7.1.2 Screenshots: Injunctive-norms

We would like to highlight some consequences of your choices:

You will have noticed that you can obtain a **personal benefit** by choosing action H (=40 points) over action G (=8 points).
But be aware that this benefit in points comes at a **potential cost paid by others, and even by yourself**.
If you are a purple player and choose action H, there is a higher chance that a blue player you are paired with will turn purple (thereby losing their bonus money for the game).
If you are a blue player paired with a purple player and you choose action H, then you will have a higher chance of turning purple yourself (thereby losing your bonus money for the game).

All points scored across the rounds are worth nothing to a purple player.

Choosing action H may therefore endanger your own bonus money and the bonus money of other participants.
Choosing action G protects others and yourself from changing into purple players and losing bonus money.

In brief:
Choose action G to protect your and other players' bonus money.

Figure S31
Instructions (injunctive norms)

S7.1.3 Instructions: Chain reaction condition

[*These instructions consist of nine pages, each featuring an image of the game development. All images can be seen in the screenshots, presented in order after the text collection.*]

Page 1/9. We would like to show you an example of how a single change of player color can result in a chain reaction of color changes.
In this example, one player begins the first round as a purple player and is paired with a blue player.

Page 2/9. Now imagine that—as a consequence of both players' actions—the blue player changes to purple.
There is now one more purple player in the game who will not receive a bonus payment at the end. But the effects of this change do not end here.

Page 3/9. The purple player will meet 24 other players over the course of the game. It is possible that none of these 24 other players change color.
But if only one of the two players chooses action H in each round, the chance of changing color would be 15% in each round (and it would be 25% if both choose H).

Page 4/9. In this example, 5 of the 24 players change color to purple.
In total, there are now six more players who have changed color as a consequence of the first pair of actions.
But the effects of the initial change do not end here.

Page 5/9. Each of the five purple players who changed color (as a consequence of being paired with the player who changed first) will meet other players during the remaining rounds of the game.

Page 6/9. In this example, 19 more players (in addition to the previous 6) change color due to these interactions.

Page 7/9. In total, there are now 25 players who have changed color as a consequence of the first pair of actions. But the effects of the initial change do not end here.

Page 8/9. Each of the 19 players at the end of the previous chain will meet other players in the remaining rounds of the game, and so forth.

Page 9/9. Many more players may change color (and may lose their bonus for this game) as a consequence of the very first pair of choices.

Players can change their color for other reasons as well and some of the dots above might actually be the same player (since two players may meet the same third player in different rounds)—but this does not change the lesson of this example:

In this game, if one player changes color, other players (potentially many players) may also change color in a chain reaction.

The chance of changing color is higher when at least one player chooses action H and highest when both choose action H.

S7.1.4 Screenshots: Chain reaction

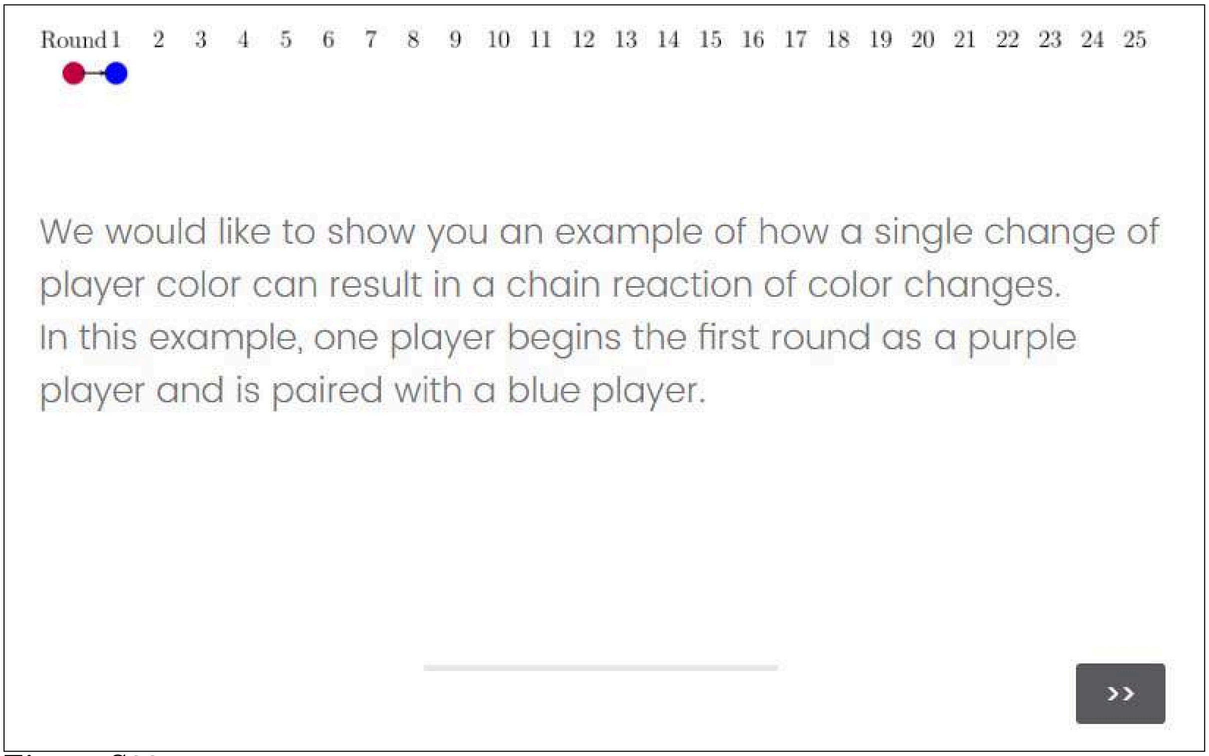

Figure S32

Instructions (chain reaction, page 1/9)

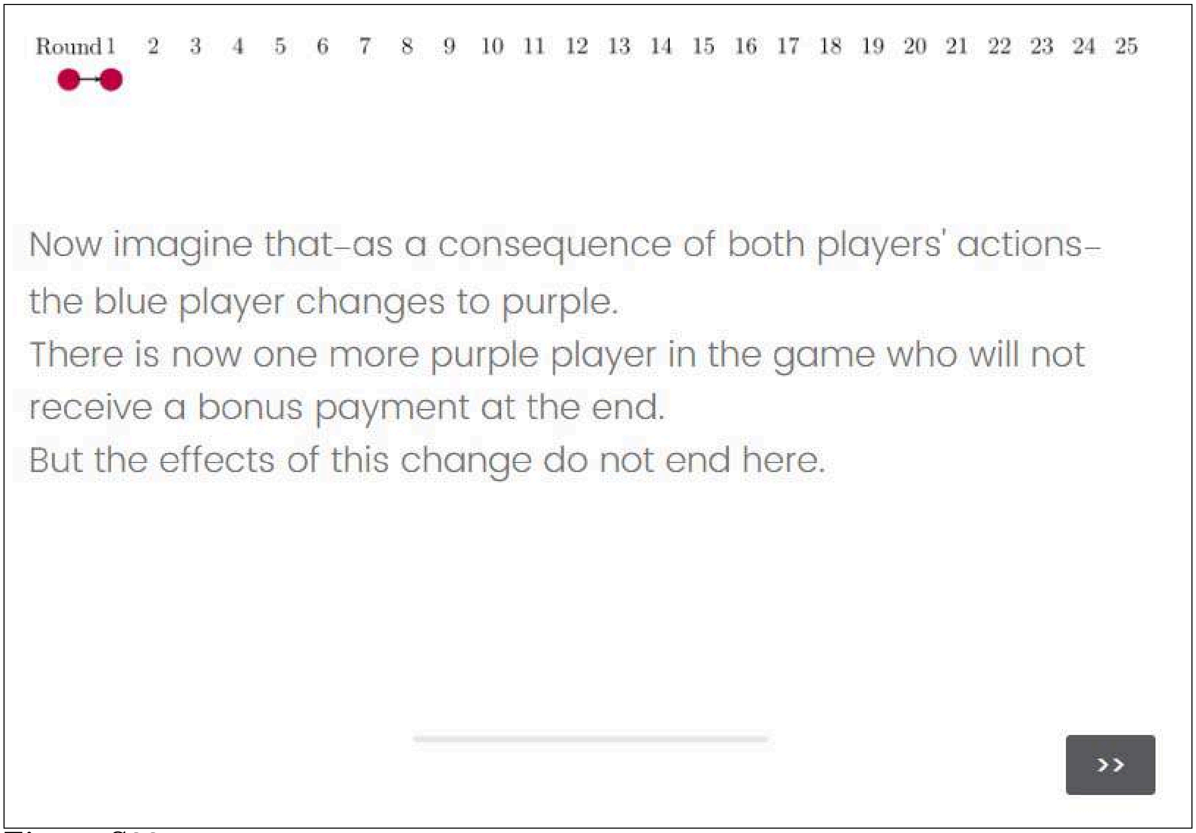

Figure S33
Instructions (chain reaction, page 2/9)

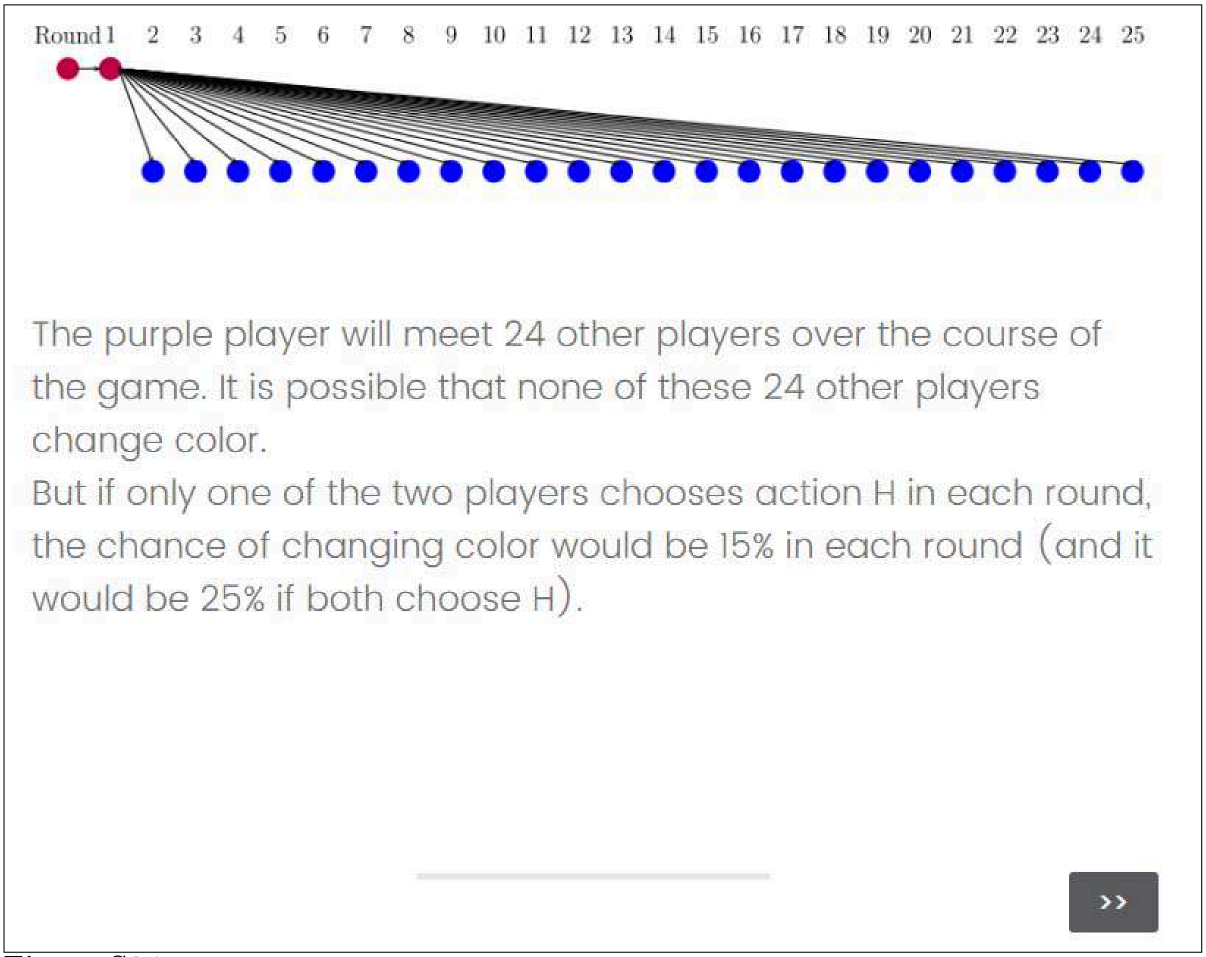

Figure S34

Instructions (chain reaction, page 3/9)

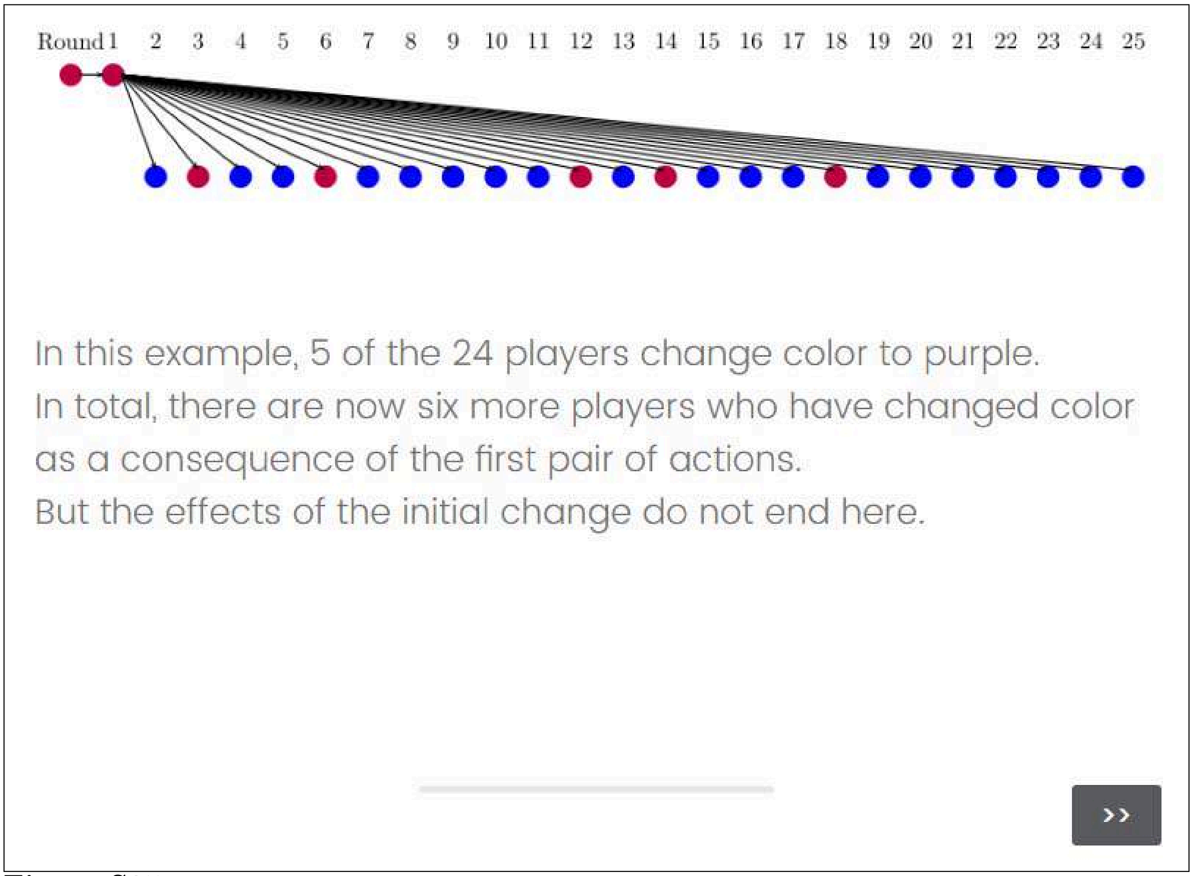

Figure S35
Instructions (chain reaction, page 4/9)

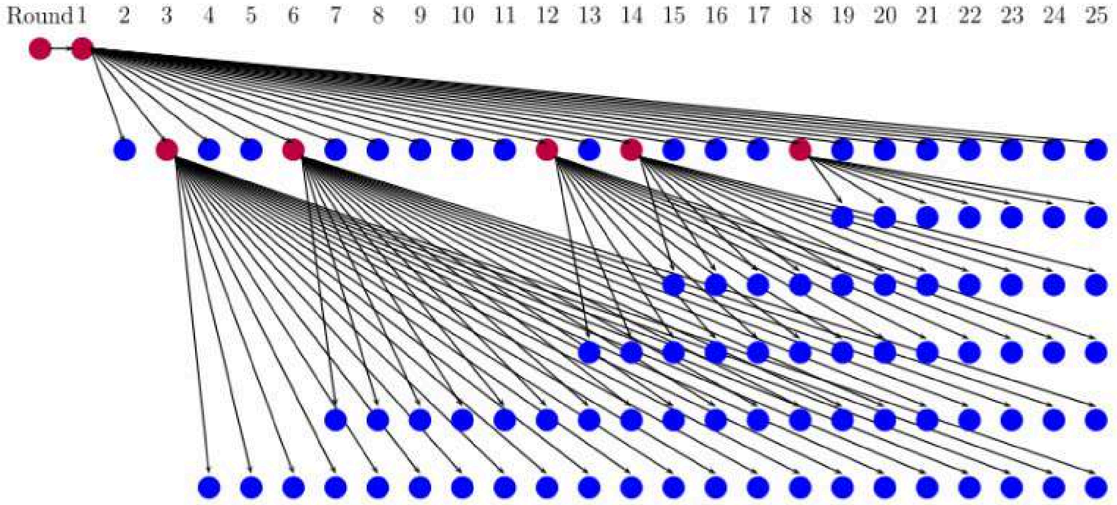

Each of the five purple players who changed color (as a consequence of being paired with the player who changed first) will meet other players during the remaining rounds of the game.

>>

Figure S36

Instructions (chain reaction, page 5/9)

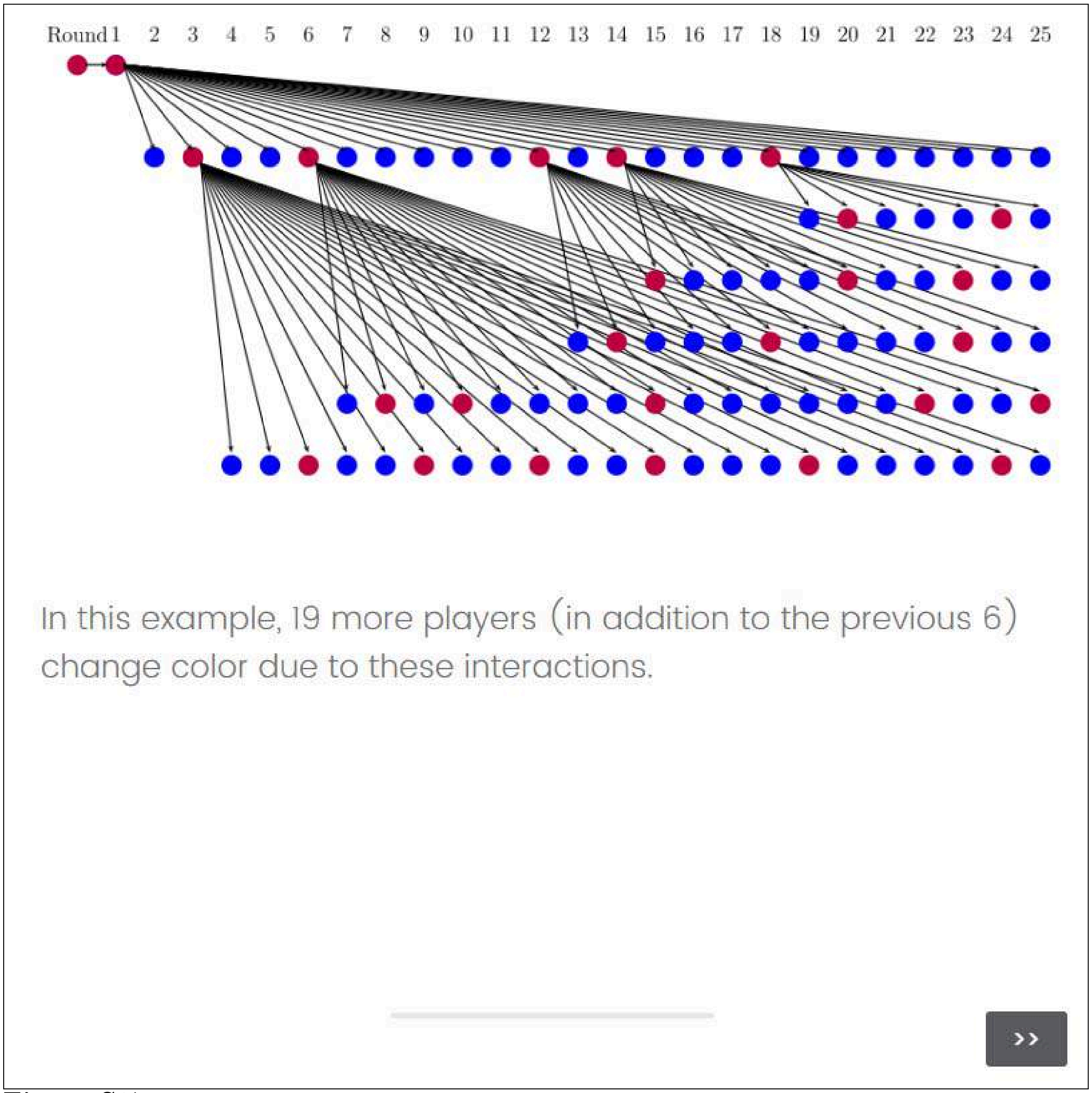

Figure S37
Instructions (chain reaction, page 6/9)

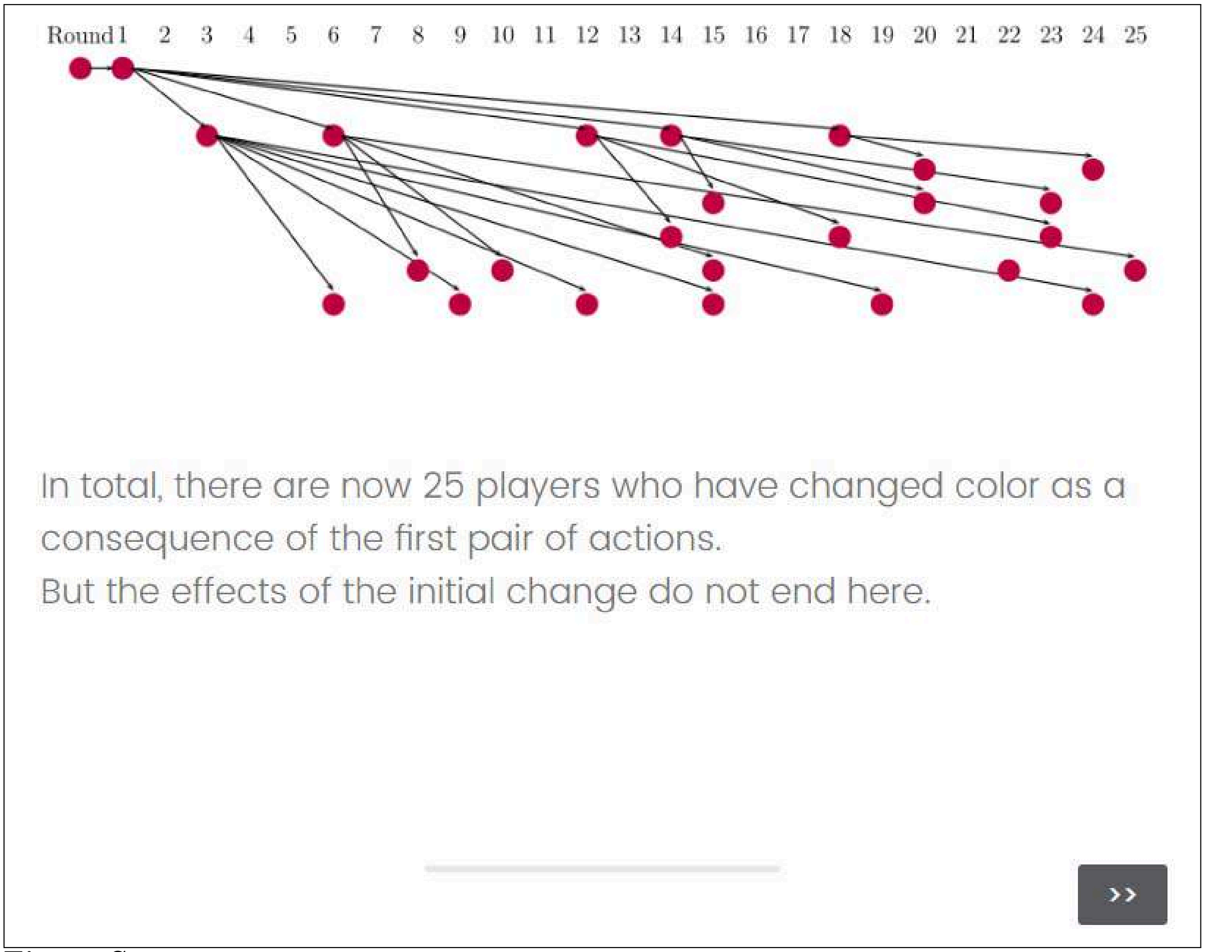

Figure S38
Instructions (chain reaction, page 7/9)

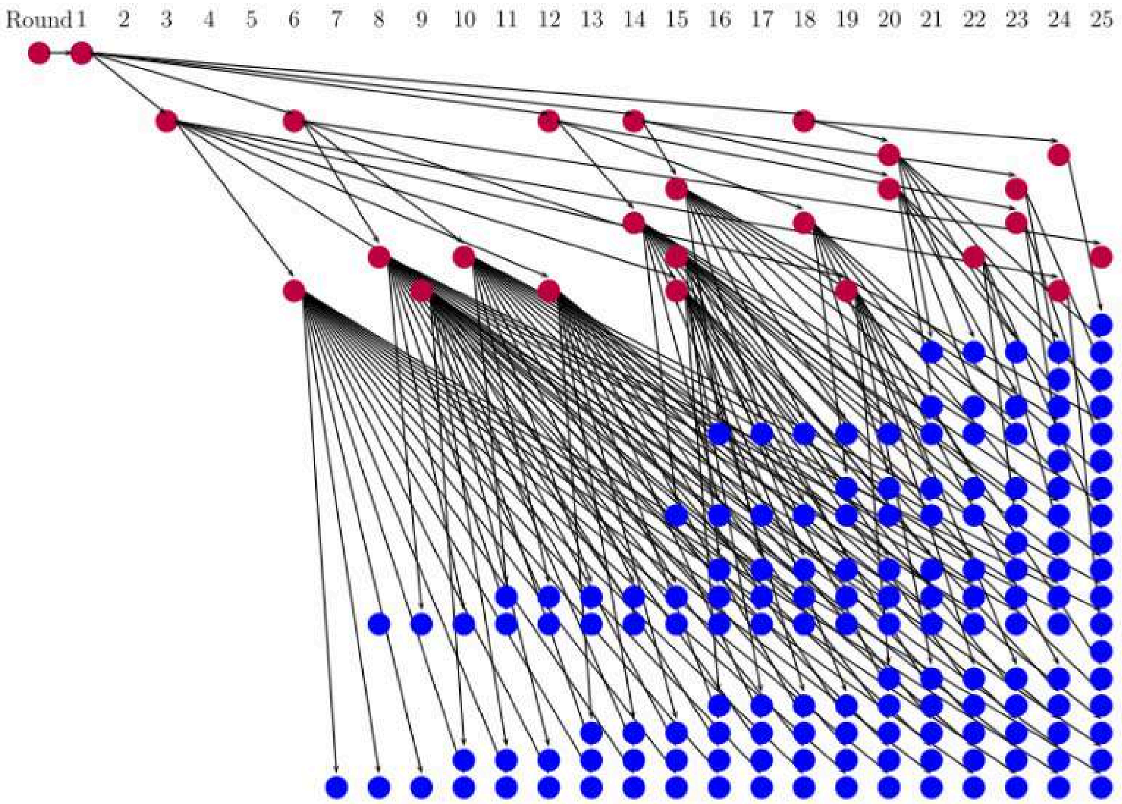

Each of the 19 players at the end of the previous chain will meet other players in the remaining rounds of the game, and so forth.

\>>

Figure S39

Instructions (chain reaction, page 8/9)

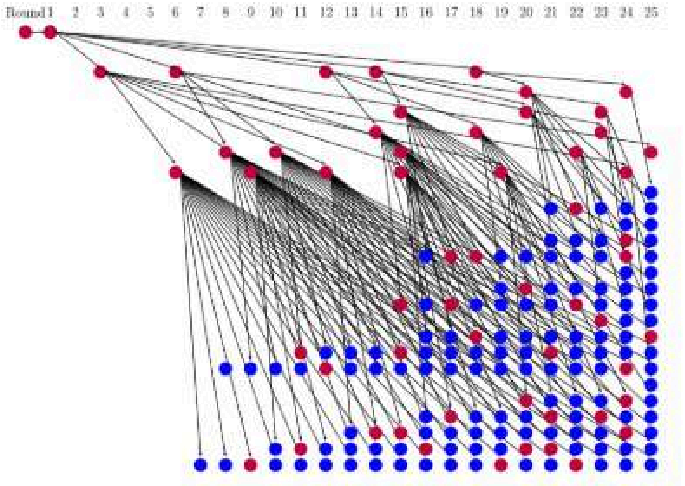

Many more players may change color (and may lose their bonus for this game) as a consequence of the very first pair of choices.

Players can change their color for other reasons as well and some of the dots above might actually be the same player (since two players may meet the same third player in different rounds)—but this does not change the lesson of this example:

In this game, if one player changes color, other players (potentially many players) may also change color in a chain reaction.

The chance of changing color is higher when at least one player chooses action H and highest when both choose action H.

››

Figure S40

Instructions (chain reaction, page 9/9)

S7.1.5 Instructions: Simulator condition

Page 1/2. Before starting the game, you will see simulations of how the game could play out.
These simulations explore the relationship between player decisions and color changes.
There are many different ways that 100 players in this game could decide, and it would take a long time to study all of them.

The simulations therefore focus on games in which each player follows a simple rule: They either choose action H all the time or they choose action G all the time.
You will be able to observe five game types:

1. **99% H: 99** of the 100 players always choose **H** (and **1** player always chooses **G**).
2. **75% H: 75** of the 100 players always choose **H** (and **25** players always choose **G**).
3. **50% H: 50** of the 100 players always choose **H**(and **50** players always choose **G**).
4. **10% H: 25** of the 100 players always choose **H** (and **75** players always choose **G**).
5. **1% H: 1** of the 100 players always chooses **H** (and **99** players always choose **G**).

You will find the simulator on the following page.

Page 2/2. You can simulate games by clicking on the buttons below the graph. The button label tells you which type of game is simulated. For example, the "75% H" button will simulate a game in which 75 players always choose option H and 25 players always choose option G for all 25 rounds, as described before.

Each simulation result will be shown in the graph below.

All the way on the right (on top of the y-axis), you can see how many of the 100 players changed to purple after the 25th round. The higher the point, the more players are purple (between 0 and 100). All these players would not receive any bonus money if the game were real, not simulated.

All the way to the left (above the 0 on the x-axis), you can see how many of the 100 players start the first round as purple players (always 8).

In between, you can see how many players are purple at the end of each round.

As you know, outcomes in the game partly depend on chance. Therefore, two games of the same type might result in different numbers of purple players. By simulating a game type several times, you can experience how different these numbers can be.
You can simulate each game type as often as you like. Each time you will see an entirely new game from start to finish. Each game starts with 100 blue players and the

eight purple players are determined at random. Different players will be paired and actions may result in different consequences.

You need to click each button at least three times before you can continue to the next page. By clicking on the same button, you can observe how the same type of game can result in different consequences when played several times. By clicking on different buttons, you will be able to compare different game types to see how game results depend on player behavior.

Each button click will simulate a new game (the order of clicks does not matter).

[*The simulator is presented below this page, see screenshot.*]

S7.1.6 Screenshots: Simulator

[*The instructions are shown in Figure S41, Figure S42, and Figure S43. The simulator part in Figure S43 reacts to button clicks by simulating single games with the specified rate of risk-seeking decision makers, followed by an animated transition of the results graph showing the number of purple players across rounds in the simulated game. The simulations are done on the participant's computer individually. The screenshots contain examples for simulations with 99% (see Fig. S44) and 25% (see Fig. S45). There are more examples in* ***SM2****, together with the JavaScript code for the simulator. Numbers shown in button captions are counted down with each click, and the continue button is shown once all counters reach zero. There is no enforced upper limit on the number of simulations per participant.*]

Before starting the game, you will see simulations of how the game could play out.
These simulations explore the relationship between player decisions and color changes.
There are many different ways that 100 players in this game could decide, and it would take a long time to study all of them.

The simulations therefore focus on games in which each player follows a simple rule: They either choose action H all the time or they choose action G all the time.
You will be able to observe five game types:

1. **99% H**: **99** of the 100 players always choose **H** (and **1** player always chooses **G**).
2. **75% H**: **75** of the 100 players always choose **H** (and **25** players always choose **G**).
3. **50% H**: **50** of the 100 players always choose **H** (and **50** players always choose **G**).
4. **10% H**: **25** of the 100 players always choose **H** (and **75** players always choose **G**).
5. **1% H**: **1** of the 100 players always chooses **H** (and **99** players always choose **G**).

You will find the simulator on the following page.

>>

Figure S41
Instructions (simulator, page 1/2)

You can simulate games by clicking on the buttons below the graph. The button label tells you which type of game is simulated. For example, the "75% H" button will simulate a game in which 75 players always choose option H and 25 players always choose option G for all 25 rounds, as described before.

Each simulation result will be shown in the graph below.

All the way on the right (on top of the y-axis), you can see how many of the 100 players changed to purple after the 25th round. The higher the point, the more players are purple (between 0 and 100). All these players would not receive any bonus money if the game were real, not simulated.

All the way to the left (above the 0 on the x-axis), you can see how many of the 100 players start the first round as purple players (always 8).

In between, you can see how many players are purple at the end of each round.

As you know, outcomes in the game partly depend on chance. Therefore, two games of the same type might result in different numbers of purple players.
By simulating a game type several times, you can experience how different these numbers can be.

You can simulate each game type as often as you like. Each time you will see an entirely new game from start to finish.
Each game starts with 100 blue players and the eight purple players are determined at random.
Different players will be paired and actions may result in different consequences.

You need to click each button at least three times before you can continue to the next page.
By clicking on the same button, you can observe how the same type of game can result in different consequences when played several times.
By clicking on different buttons, you will be able to compare different game types to see how game results depend on player behavior.

Each button click will simulate a new game (the order of clicks does not matter).

Figure S42

Instructions (simulator, page 2/2, upper part)

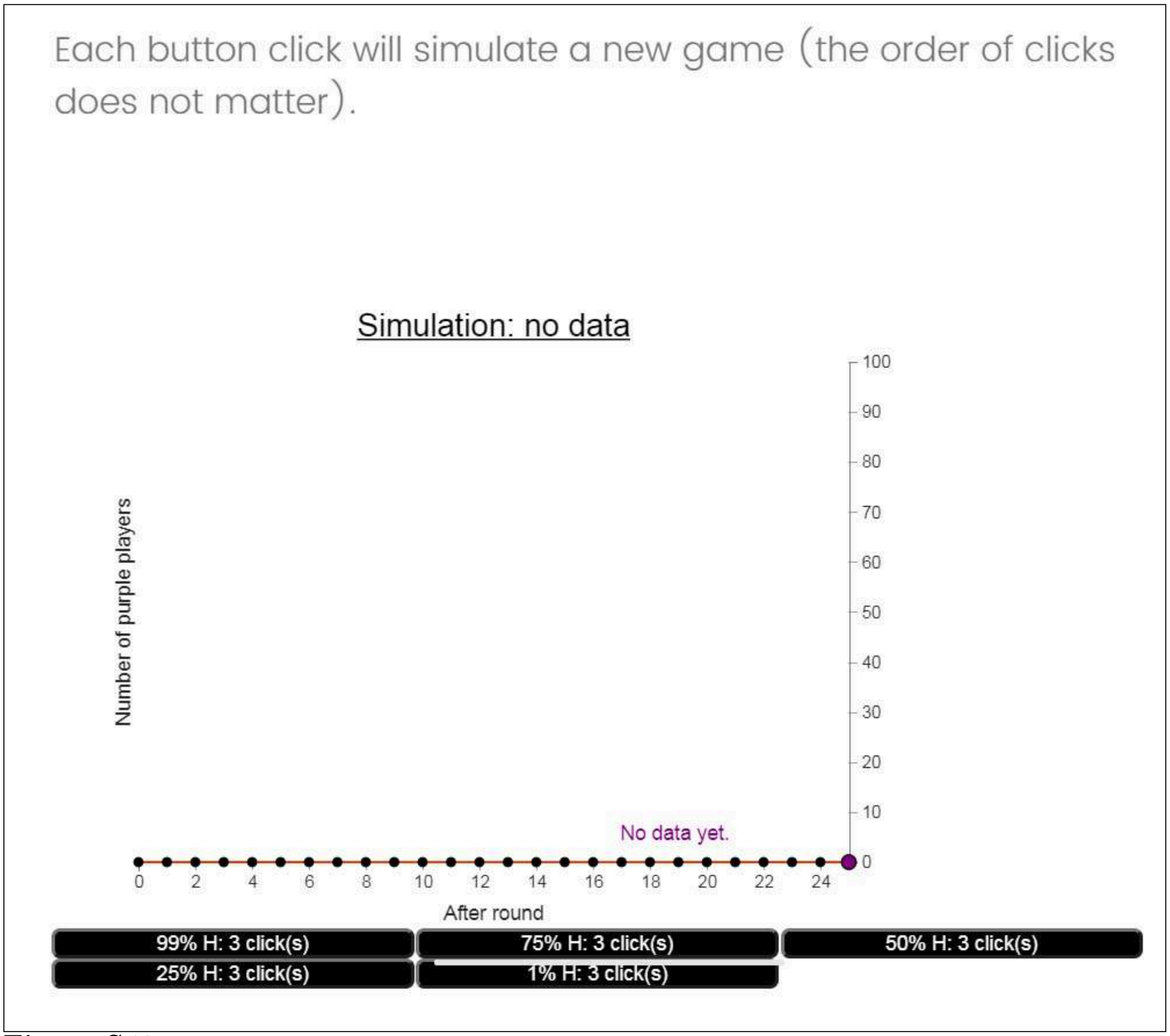

Figure S43

Instructions (simulator, page 2/2, lower part)

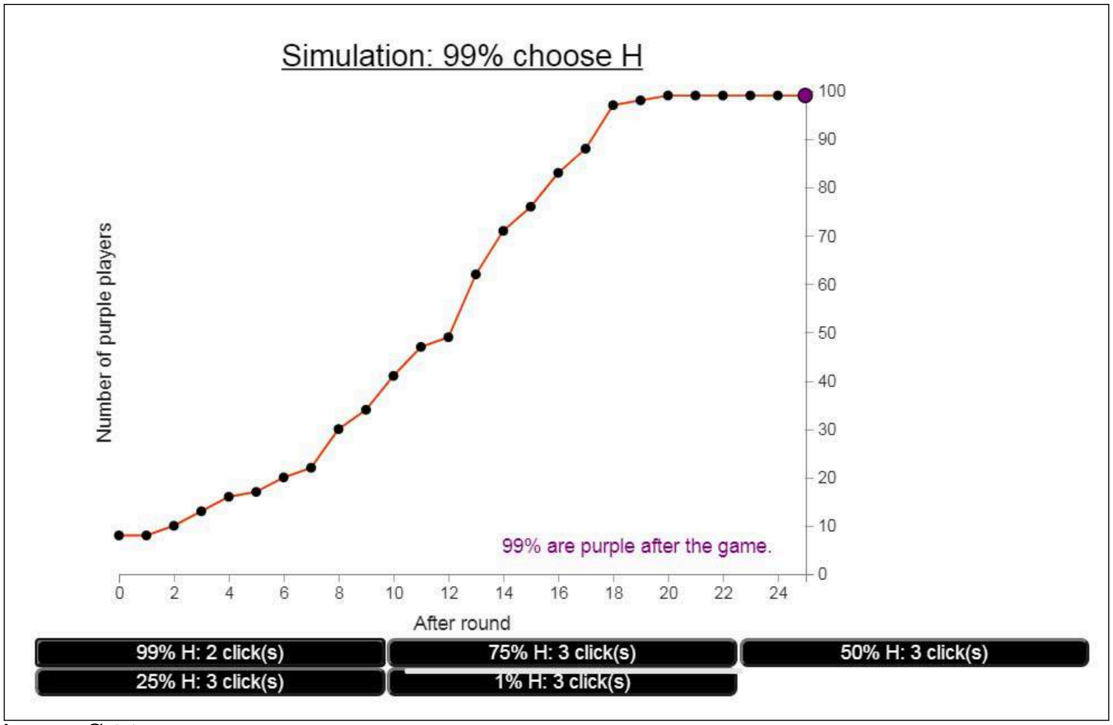

Figure S44
Instructions (simulator, example of 99% H results)

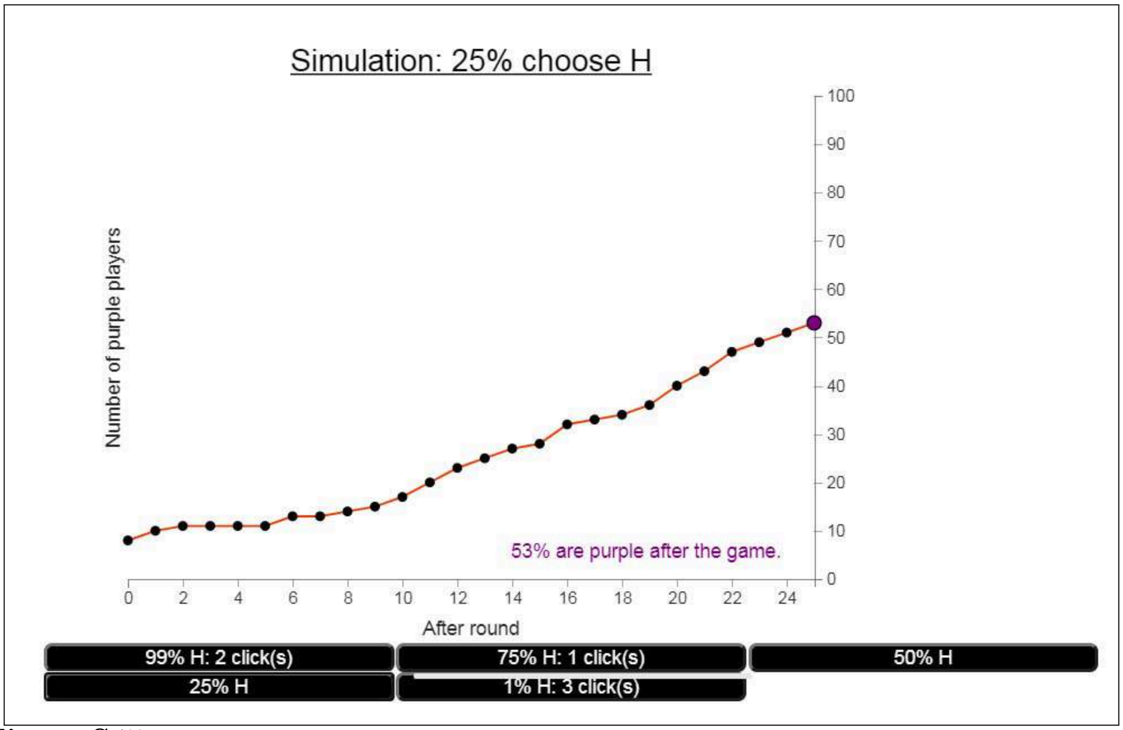

Figure S45
Instructions (simulator, example of 25% H results)

S7.2 Condition-specific parts in the game

S7.2.1 Introduction: Addition in the vicarious learning condition

[*The following text was shown below the standard introduction part (see Fig. S46).*]

In the course of the game, we will inform you about a group of participants that played the game earlier.

Before each decision, you will be able to see how many players in the previous group were purple at the beginning of each round of their game.
A graph will show you these numbers up to the round you are about to play.

This number may differ from the number of purple players in your own game.

S7.2.2 Introduction: Addition in the descriptive norms condition

[*The following text was shown below the standard introduction part (see Fig. S47).*]

In the course of the game, we will inform you about a group of participants that played the game earlier.

Before each decision, you will be able to see how many players in the previous group chose action G in the round you are about to play.

This number may differ from the number of players choosing G in your own game.

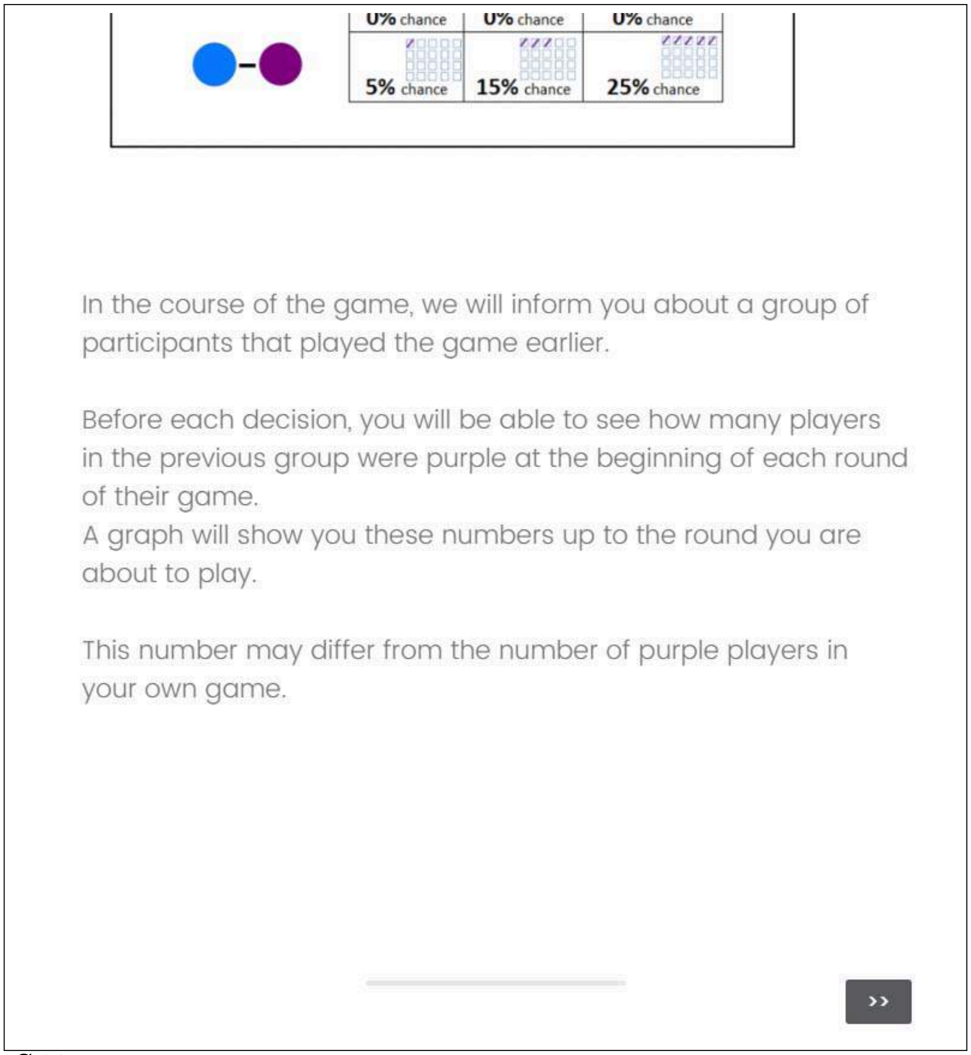

Figure S46

Instructions (vicarious learning condition, the text was shown below the standard instructions)

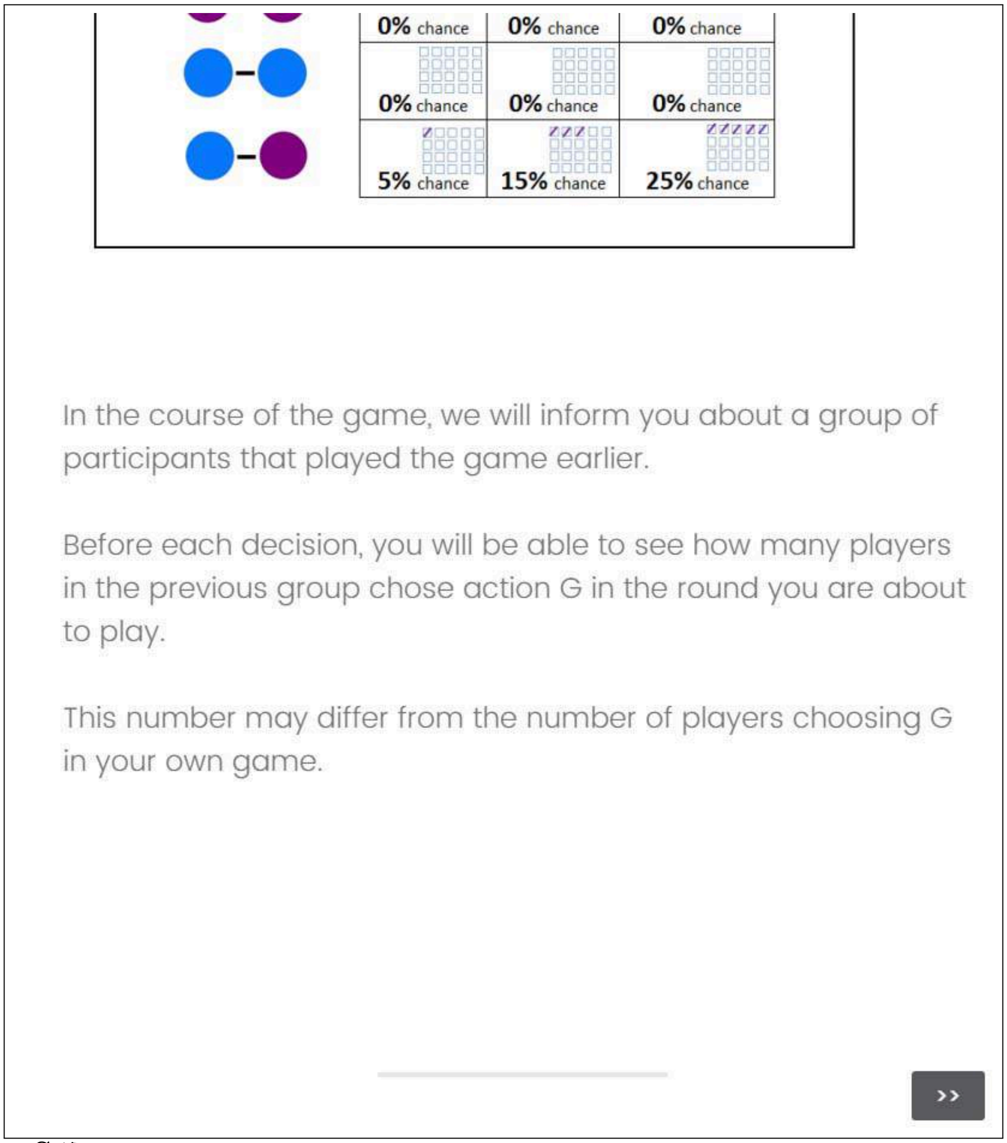

Figure S47

Instructions (descriptive-norms condition, the text was shown below the standard instructions)

S7.2.3 Rounds (vicarious learning condition only)

[*See Figure S48, Figure S49, and Figure S50.*]

Round [*current round*]/25
Points gained so far: [*current number of points*] (+ [*points gained in previous round*])
Possible bonus: £[*current bonus*] (+£[*bonus amount gained in previous round*])
Points will be converted into bonus money after the final round (for blue players only).

[*Graph of the number of purple participants in the previous group up to before the current round.*]

Above you can see information about the previous group of participants at the same point in the game.

Which of the two actions do you choose?

- Action G: I receive 8 points. (8)
- Action H: I receive 40 points. (40)

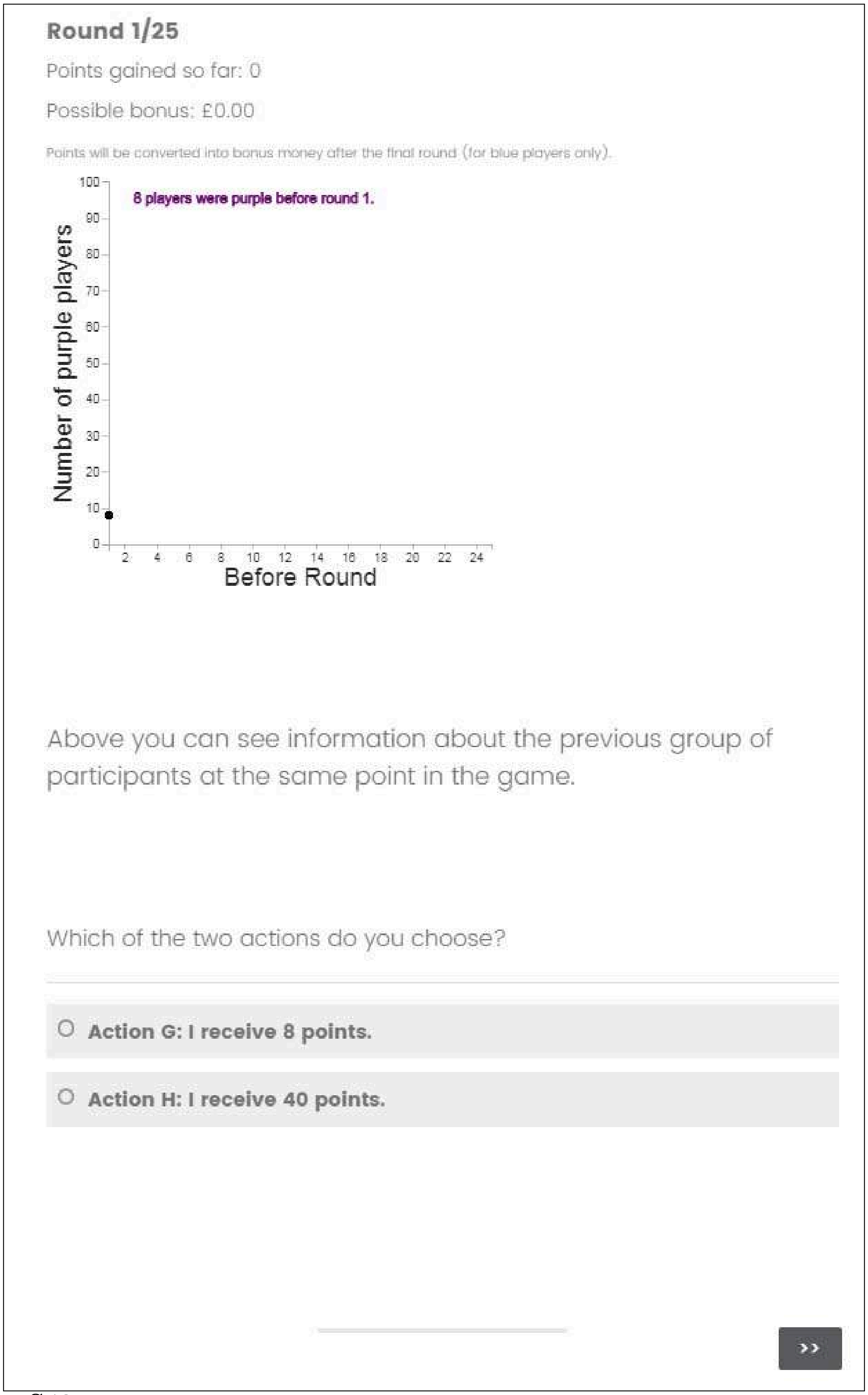

Figure S48

Game round 1 (example in vicarious condition)

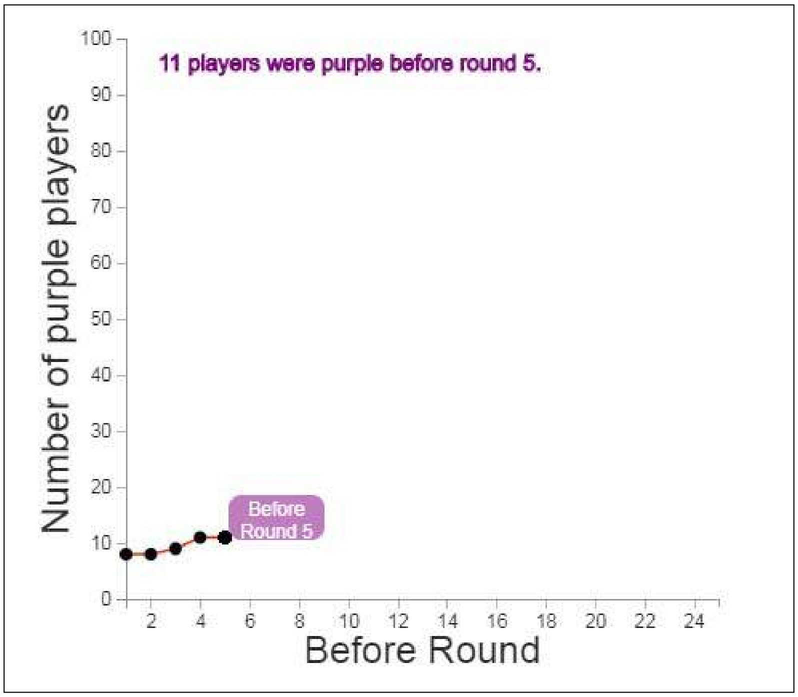

Figure S49

Graph in game round 5 (example in vicarious condition with tooltip shown)

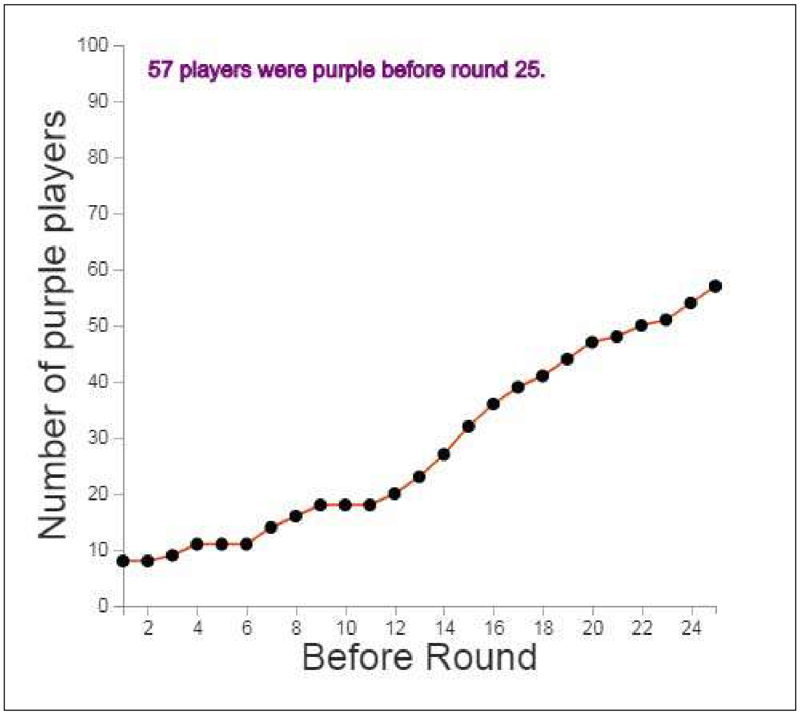

Figure S50

Graph in game round 25 (example in vicarious condition)

S7.2.4 Rounds (descriptive norms condition only)

[*All participants in this condition saw the same observed numbers, see Figure S51 and Figure S52.*]

Round [*current round*]/25
Points gained so far: [*current number of points*] (+ [*points gained in previous round*])
Possible bonus: £[*current bonus*] (+£[*bonus amount gained in previous round*])
Points will be converted into bonus money after the final round (for blue players only).

In the previous group of participants, [*number of participants in previous group who chose G*] of 100 participants chose action G in round [*current round number*].

Which of the two actions do you choose?

- Action G: I receive 8 points. (8)
- Action H: I receive 40 points. (40)

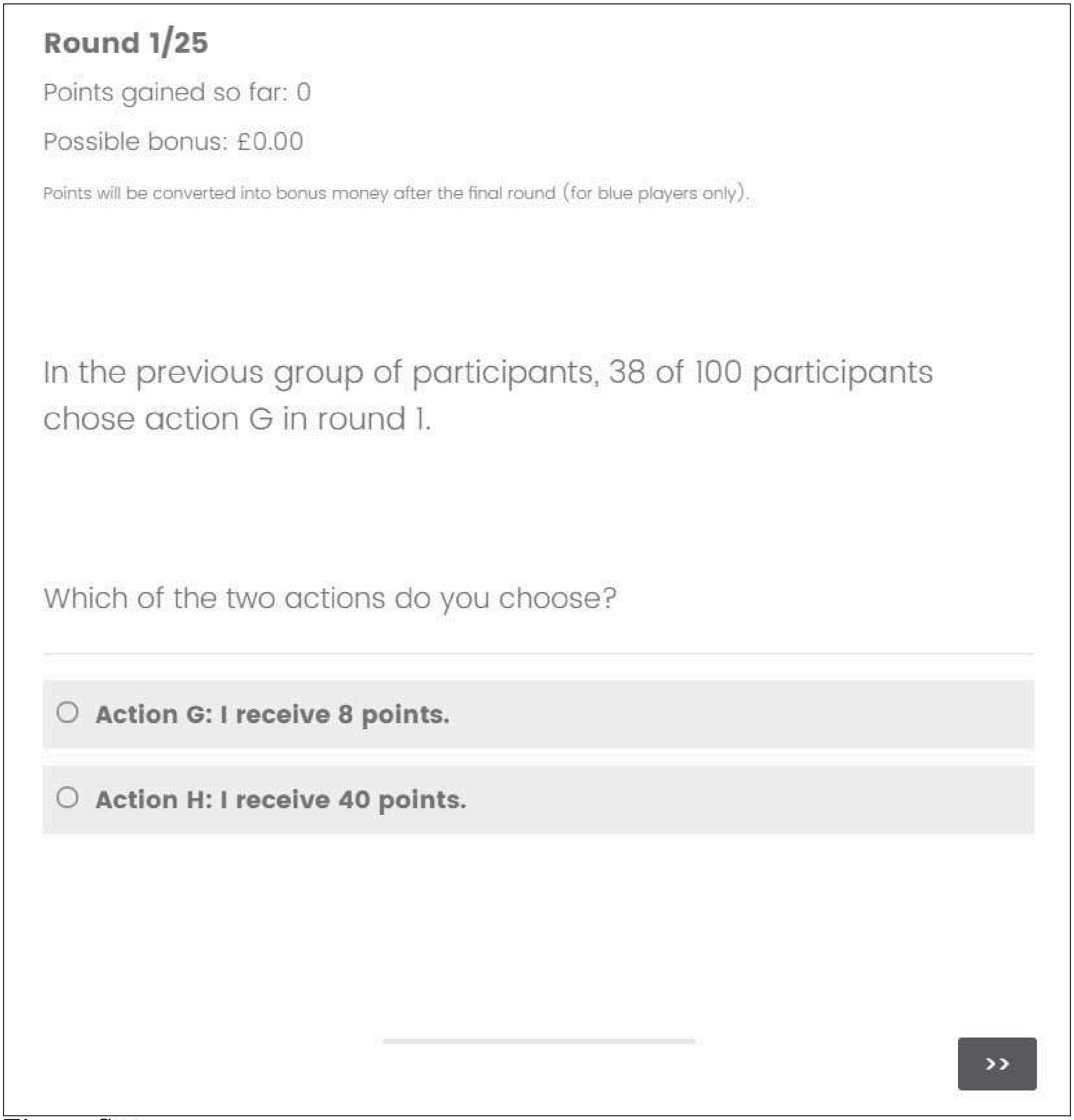

Figure S51

Interface in game round 1 (example in descriptive-norms condition)

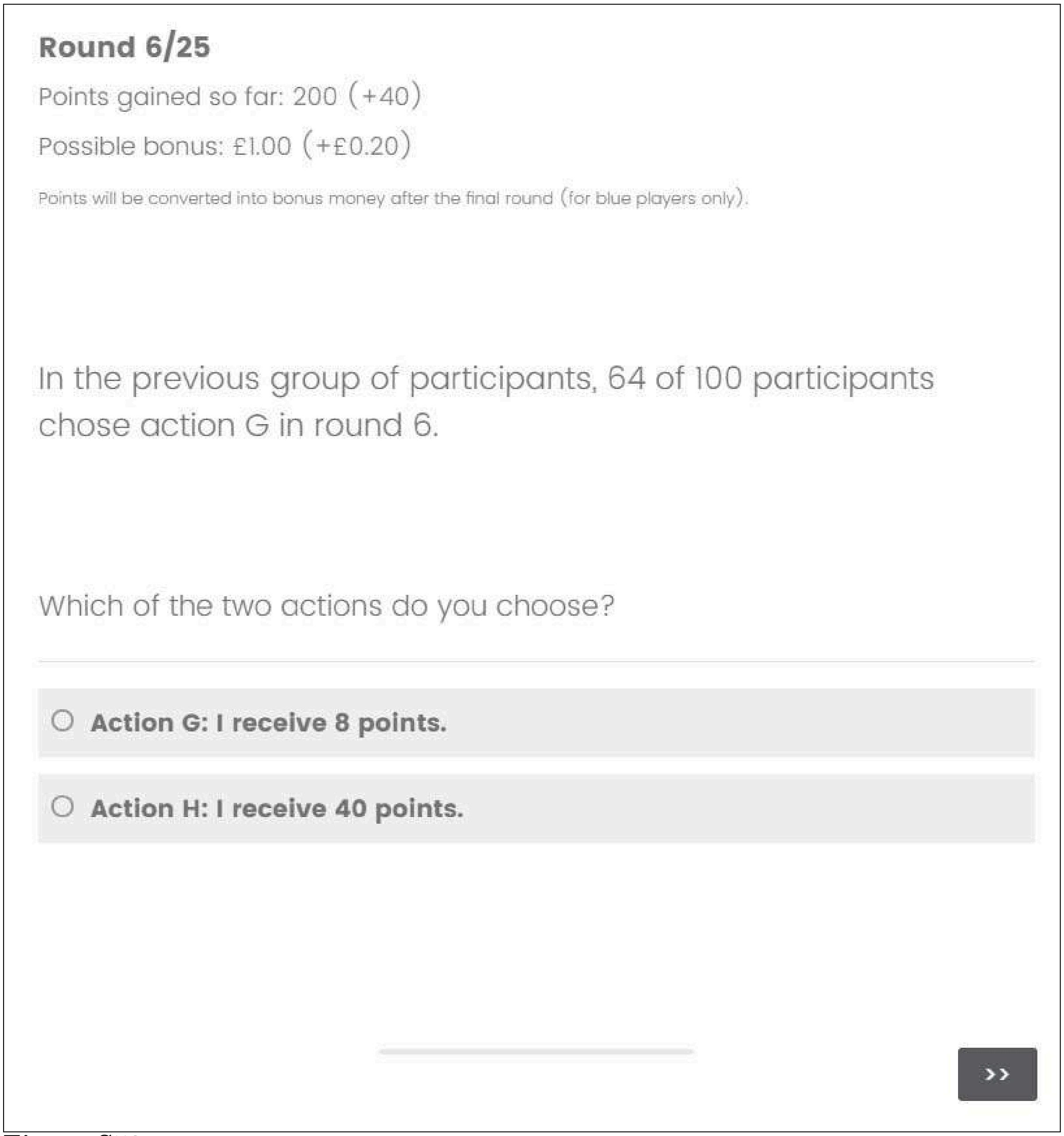

Figure S52

Interface in game round 6 (example in descriptive-norms condition)

S7.2.5 Rounds (injunctive norms condition only)

[*The same message was added in all rounds; see Figure S53.*]

Round [*current round*]/25
Points gained so far: [*current number of points*] (+ [*points gained in previous round*])
Possible bonus: £[*current bonus*] (+£[*bonus amount gained in previous round*])
Points will be converted into bonus money after the final round (for blue players only).

Choose action G to protect your and other players' bonus money.

Which of the two actions do you choose?

- Action G: I receive 8 points. (8)
- Action H: I receive 40 points. (40)

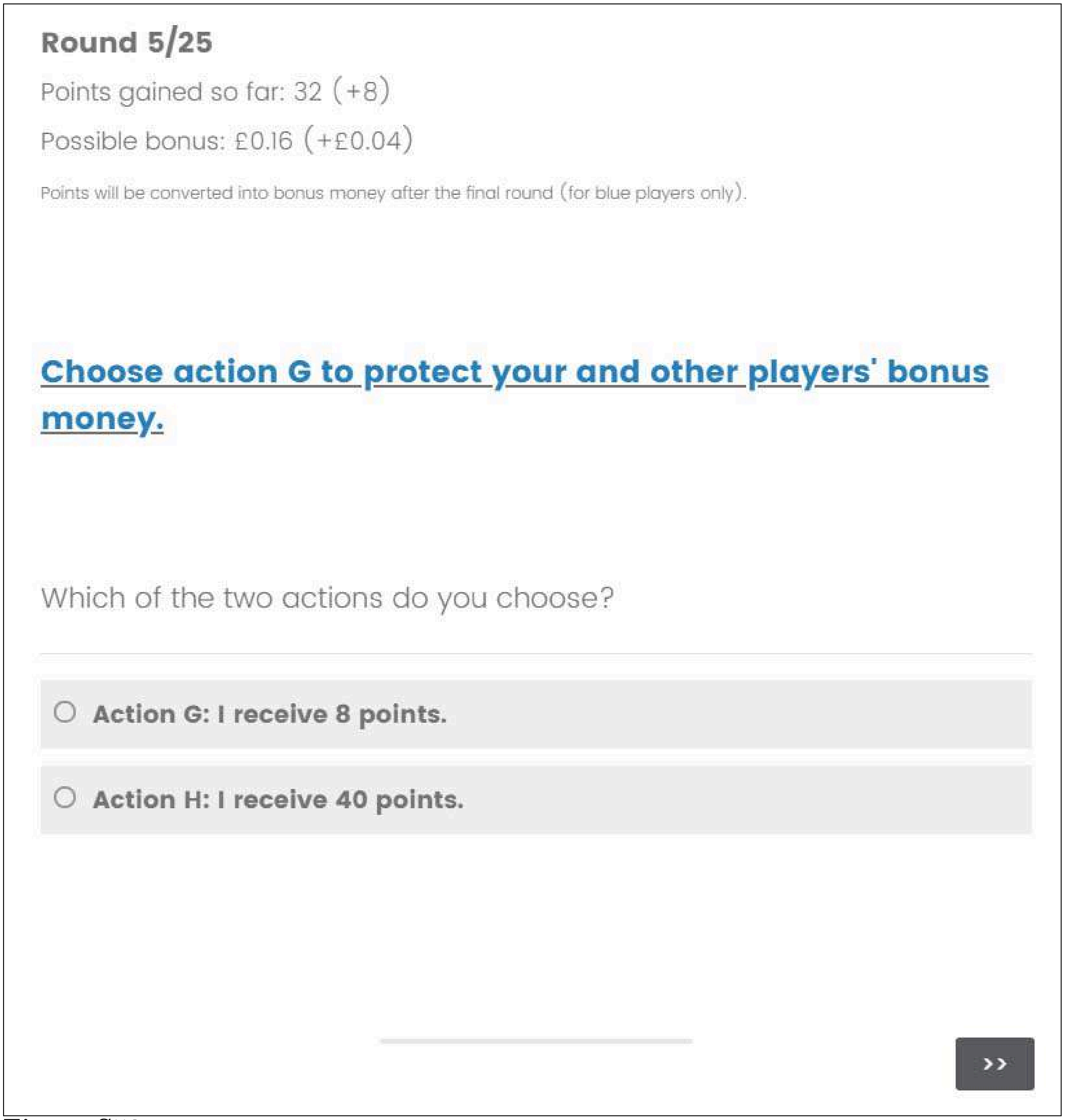

Figure S53

Game round 5 (example in injunctive-norms condition)

S8 Study 2: Selected Results

Note: The full set of results is found in document ***SA2*** *(Supporting Analyses Study 2). Variables and measures are described in document* ***VM2*** *(both at* *`https://doi.org/10.7910/DVN/ILDEMO`*).*

You can find the full set of analyses and the R code for calculations and figure creation in **SA2**. Here, we show the main results relating to the game, and some results linking political attitudes and COVID-19 related scales.

S8.1 Behavior in the transmission game

S8.2 Risky choices by round

Figure S54 allows for a comparison of game behavior across rounds between conditions. One condition is set apart from the others: players in the injunctive norms condition chose safer actions more frequently than in any other condition. In the control condition, the majority initially chose the risky option and, similar to Study 1, the frequency of risky choices decreased over the course of the game with a slight increase during the final rounds. Both in the simulator and the chain reaction condition, H-choices tended to be less frequent than in the control condition. H-choices in the descriptive norms condition, on the other hand, tended to be more frequent than in the control condition. In the vicarious learning condition, risky choices started at a higher frequency than in the control condition but ended at a lower frequency than in the control condition in the final rounds.

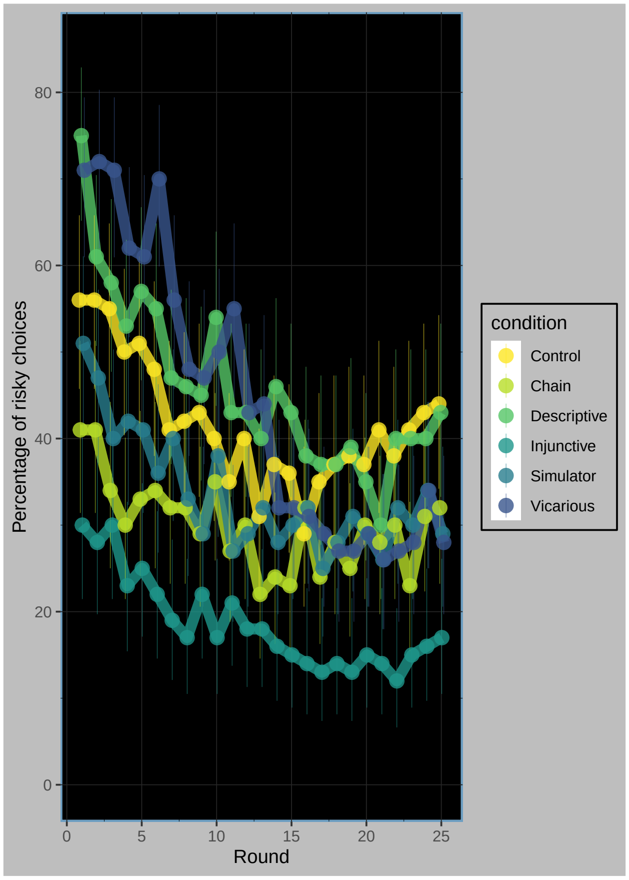

Figure S54

Transmission Game round-wise results in Study 2: Points mark the percentage of H-choices in each round for the six groups, and error bars mark the 95-percent CI for percentages.

S8.3 Postquestionnaire-results

The postquestionnaires consisted of several parts. All participants responded to the first questionnaire, measuring expectations and general game motivation. Participants in the intervention conditions also responded to a second postquestionnaire with additional, intervention-specific questions.

S8.3.1 Expectations

Participants were asked to estimate the proportion of purple participants after several rounds and the proportion of H-actions chosen in several rounds. Figure S55 plots individual estimates and fit lines for each condition.

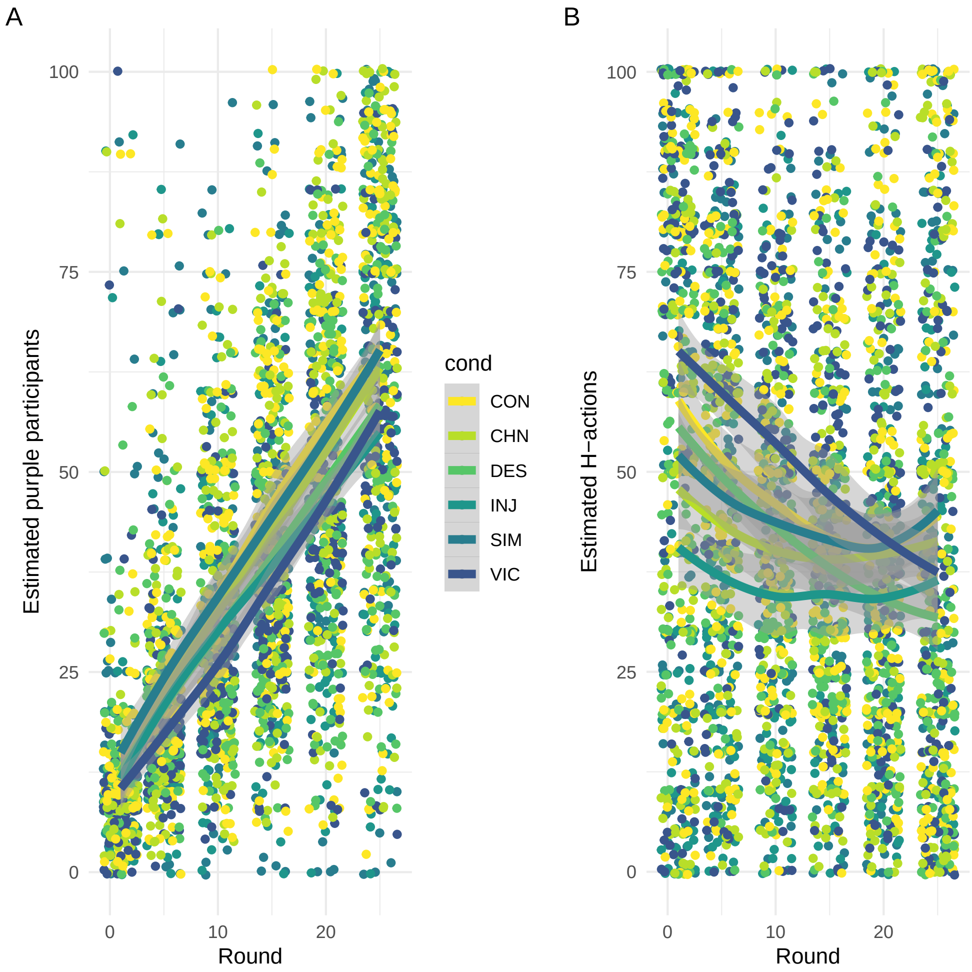

Figure S55

Postquestionnaire expectations: Plots show individual estimates (points) and trend lines split by condition (DES: descriptive-norms, VIC: vicarious learning, CON: control condition, SIM: simulator, CHN: chain condition, INJ: injunctive-norms) with condition indicated by color for (A) estimates of the number of purple participants after rounds 1, 5, 10, 15, 20, 25, and (B) estimates of the proportion of H-actions chosen in rounds 1, 5, 10, 20, and 25.

Changes in the number of purple participants are estimated to be linearly increasing over time (at least based on the average estimates). Points in the upper left and lower right of the diagram correspond to participants who might have estimated the number of blue participants, instead. The differences between conditions were relatively small (and underestimated the expected differences, see below). Slightly lower rates were estimated for the vicarious learning, injunctive norms, and descriptive norms conditions. These differences do not in general correspond to observed differences in behavior. Both the simulator and the chain reaction intervention increased the estimates for purple participants relative to the other interventions.

In contrast, the highest proportion of risky actions was expected in the vicarious learning conditions, but with a near-linear reduction and lower levels in the final rounds. Consistent with the actual behavior, the lowest proportion of H-choices was estimated in the chain reaction and injunctive norms conditions. Differences in estimated actions are larger than differences in estimated color switches. A comparison with actual expected color switches will be made in the simulation section, below.

S8.3.2 Motivations (general postquestionnaire)

The general postquestionnaire asked for participants' motivation in the game with eleven questions (on a scale from 1–5, the name at the start of each line is only used in the figures below):

- MaxBonus: I wanted to make as much bonus money as possible.
- MoreBonus: I wanted to make more bonus money than other players.
- Responsible: I felt responsible for other players.
- MakeSwitch: I wanted to make other players switch color.
- AfraidSwitch: I was afraid to switch color in this game.
- Anticipate: I tried to anticipate what others were doing in this game.
- FollowGut: I followed my gut in this game.
- TakeRisk: I wanted to take some risk in this game.
- ProtectOthers: I wanted to protect others in this game.
- DidNotCare: I did not care at all what happened in this game.
- AllReceive: I wanted that the entire group receives as much money as possible.

Relationships between these questions and game behavior are split up into two Figures (see Fig. S56 and Fig. S57).

As in Study 1, most participants indicated that they wanted to have the maximum bonus possible; fewer participants wanted to have more bonus money than others. Both answers to these questions were significantly correlated with the number of H-choices in all groups.

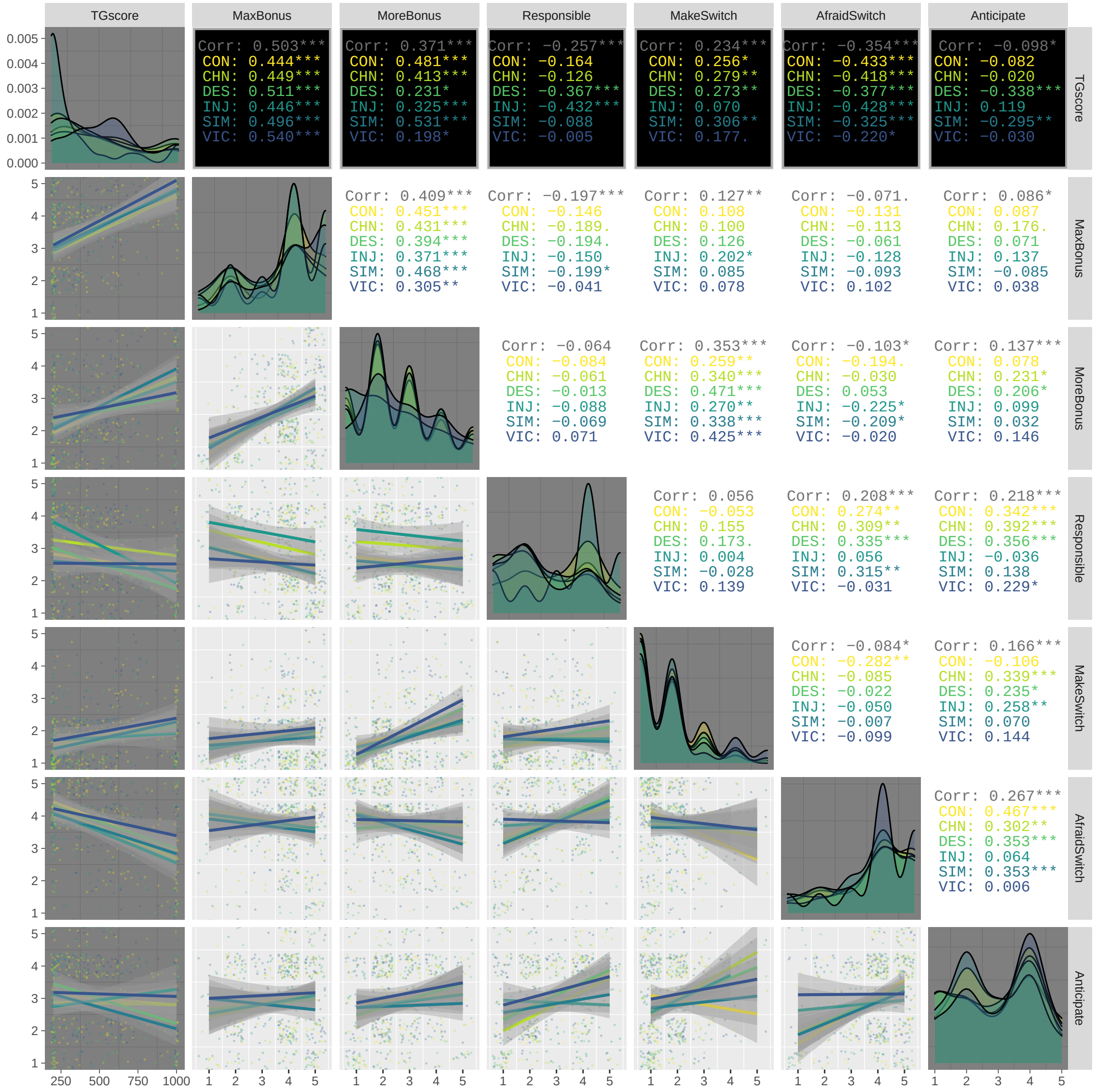

Figure S56

Relationship between game results and postquestionnaire responses (part 1): Distribution of values (diagonal), general and group-specific bivariate correlations (above diagonal) and scatter plots with linear fit line (below diagonal).

Feeling responsible and being afraid to switch were correlated negatively with the number of H-choices. Most participants were afraid to switch colors; this was more frequent in the vicarious learning condition. The negative correlation for this variable was found in each of the six groups. Feelings of responsibility for others were most pronounced in the injunctive norms condition (in which the highest negative correlation with risk-taking was observed). The second highest (and still significant) correlation was observed in the descriptive norms group. There was virtually no relationship in the vicarious learning condition, and reduced negative relationships in the rest of the groups.

Few participants responded that they wanted to make others switch color, and we observed positive correlations between this intention and the number of H-choices in all groups, most pronounced in the simulator condition and least pronounced in the injunctive norms condition. Participants were split in most conditions between answering that they had anticipated others' actions or not. There was a weak negative correlation with H-choices across all groups, with higher values in the descriptive norms and simulator condition (both of which explicitly focused on the behavior of others, either in the form of direct information or in the form of simulation assumptions).

The strongest predictor—in the statistical sense—of H-choices in the game was the response to the risk-taking question. In all groups high correlations between admitted risk-taking and the number of H-choices were found. Participants who responded affirmatively that they were following their gut tended to choose H more often, those who were willing to protect others tended to choose H less often (similar to responsibility). Few participants admitted that they did not care about their result and those tended to choose H more often, whereas those who wanted everyone to receive a similar outcome chose H less often. In general, the pattern of postquestionnaire responses aligned with behavior, with some notable differences between conditions.

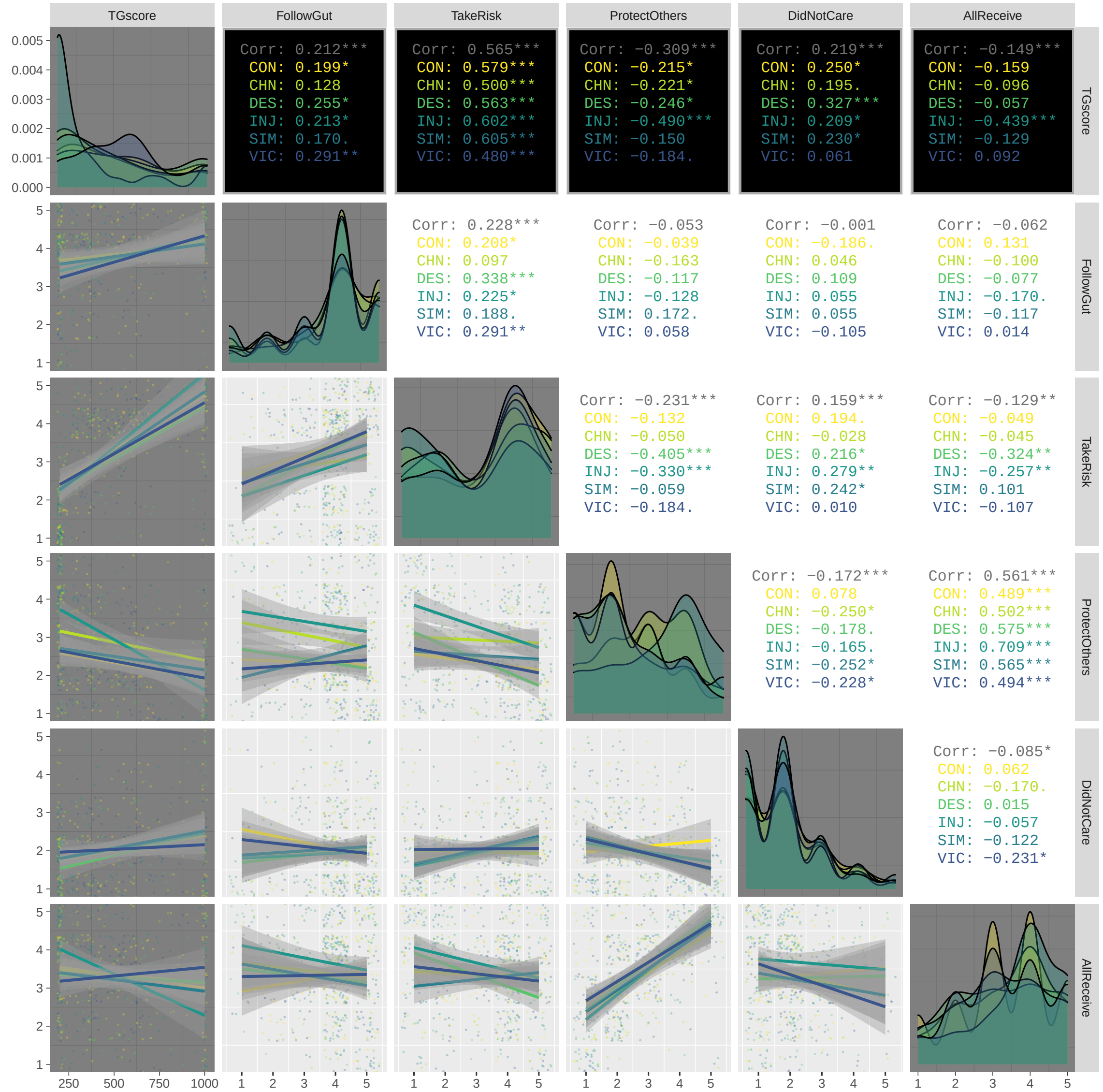

Figure S57

Relationship between game results and postquestionnaire responses (part 2): Distribution of values (diagonal), general and group-specific bivariate correlations (above diagonal) and scatterplots with linear fit line (below diagonal).

S8.3.3 Motivations (specific postquestionnaires)

To analyze the specific postquestionnaires across conditions we grouped similar questions together (see Fig. S58).

Participants in general indicated to like and understand the five interventions (vicarious learning, descriptive norm, injunctive norm, chain reaction, and simulator); they felt influenced by the experienced intervention and did not consider it a waste of their time. Few participants believed that they had reduced the number of G-choices in response to the intervention, with some of them simultaneously indicating that they had also reduced the number of H-actions.

There was a small negative correlation between liking the intervention and H-choices, mainly driven by the injunctive norms and simulator condition. Similarly, there was a negative correlation between participants' indicating that they had understood the intervention and H-choices. The strongest relationship was found in the simulator condition, which might have required the most cognitive effort to understand, followed by the vicarious learning condition (which also featured a graphical presentation), and the chain reaction condition (also a relatively complex intervention).

There was a negative correlation between indications that the interventions had influenced decisions in the game and the number of H-choices. A strong relationship was only observed in the injunctive norms and the simulator condition. Participants who felt that the intervention was a waste of their time tended to choose a larger number of H-actions. This was especially true for the injunctive norms condition.

The two questions asking participants to imagine that the intervention had not taken place seemed to have caused interpretation problems for some participants. The general direction of the relationship was as expected: Participants who affirm that they would have chosen more H-actions without the intervention selected fewer H-actions in the game. A strong relationship was only found in the simulator condition, though.

Finally, participants who indicated that they had been influenced by the intervention also tended to indicate that they had reduced the number of H-choices. These correlations were strong for all conditions. A positive correlation with being influenced and reducing the number of G-choices was only present in the vicarious learning and descriptive norms condition. This may indicate that these two interventions may have had a somewhat ambiguous effect. A subgroup of participants might have increased the number of H-choices in reaction to these interventions.

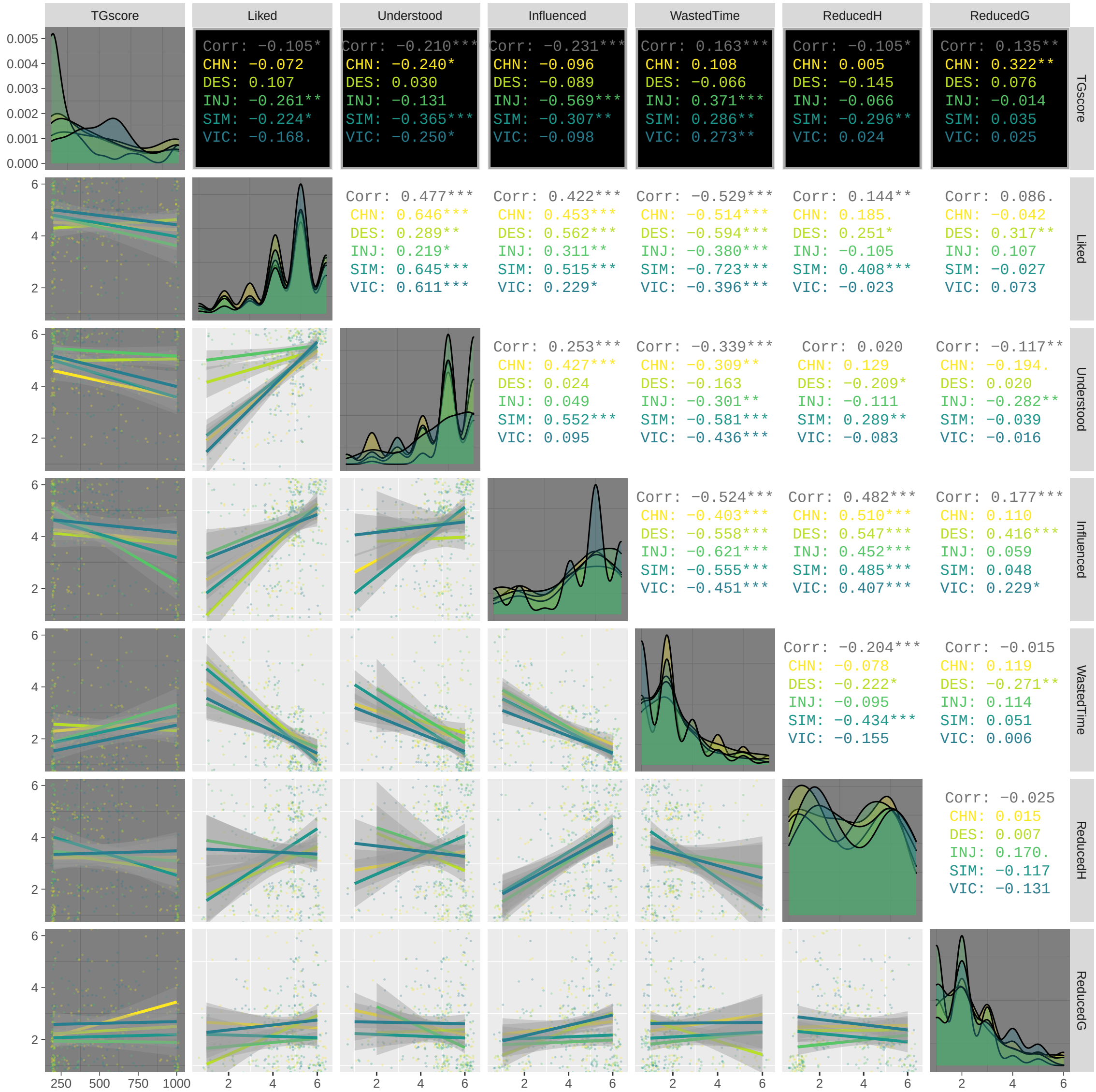

Figure S58

Relationship between game results and condition-specific postquestionnaire responses: Distribution of values (diagonal), general and group-specific bivariate correlations (above diagonal) and scatter plots with linear fit line (below diagonal).

Participants' understanding of an intervention was negatively correlated with considering it a waste of their time and highly positively correlated with liking it. This might help to explain the differences between interventions. Both the simulator and the chain reaction intervention were cognitively demanding, and not every participant might have positively engaged with them. These interventions seemed to have worked for some, but not all of the participants. In contrast, understanding the injunctive norms intervention is simpler and straight-forward.

It is possible that the vicarious learning condition signalled to some participants in the beginning that the risk was low and that H-actions could be relatively safely chosen. This corresponds to the high initial number of H-actions seen in this condition. The descriptive norms condition, in contrast, might have signalled both that a substantial number of other participants had chosen H-actions in the past and that a similarly substantial number had chosen G-actions. This intervention might therefore have had an ambiguous effect with some participants finding reasons and justifications for choosing H and others for choosing G.

S8.4 Predictor variables and game results

Figure S59 presents correlations between predictors and game results. There was a significant positive correlation between general risk-taking and the number of H-choices in every condition (it is slightly weaker in the chain reaction condition). The CRT scores were consistently and negatively correlated with the number of H-choices. This correlation was strongest in the cognitively most demanding interventions: chain reaction and simulator. There was a negative correlation between SVO angles (prosocial preferences) and the number of H-choices. This relationship was non-existent in the chain reaction and descriptive norms condition, and strongest in the injunctive norms and vicarious learning condition.

The relationships between personality scales and game behavior that we had observed in Study 1 did not materialize in Study 2. There was no systematic pattern for the honesty and the emotionality scale. We observed a negative relationship between emotionality and the risk item, a positive relationship between honesty/humility and SVO, in basically all conditions.

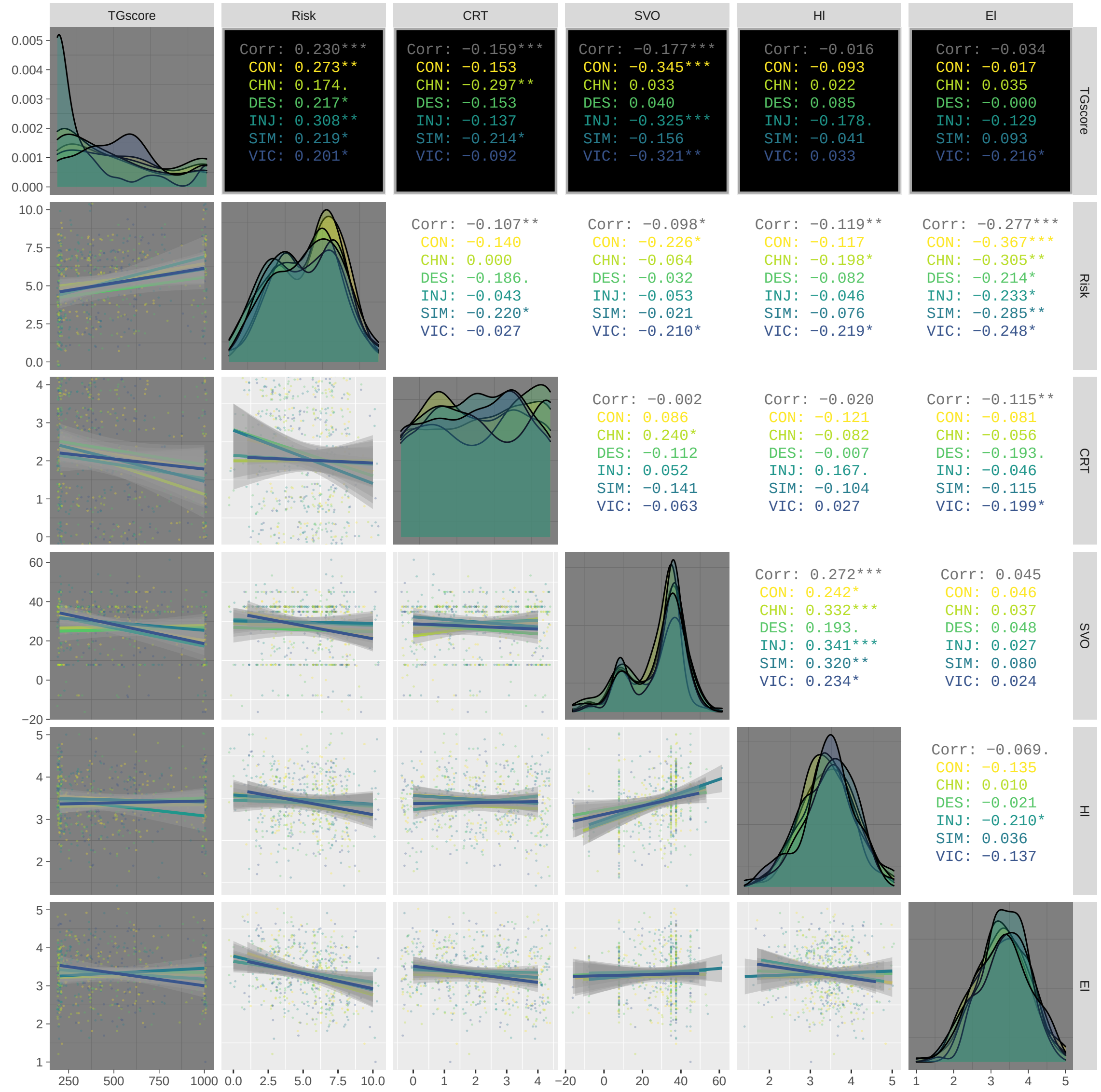

Figure S59

Predictor variables and game results: Distribution of values (diagonal), general and group-specific bivariate correlations (above diagonal) and scatterplots with linear fit line (below diagonal).

S8.5 Associations with the transmission game

An independent coder categorized comments about the game and hypothetical messages to players choosing G-actions and H-actions, respectively. The coder had no information about other variables, neither game behavior nor condition. See the Extended Supplementary Materials for an analysis of the player messages.

We asked participants whether the game reminded them of anything they had encountered before. Table S3 lists the non-exclusive categories that we used to code responses and the frequency with which each category was coded. We combined some similar categories with low frequency counts after the coding was completed. For example, all but one participant who described a social dilemma, described the prisoner's dilemma.

Only a minority of participants (14.3% with the most permissive criterion) connected the game to the novel coronavirus or epidemiology in general. This is encouraging, as it means that participants did not perceive the paradigm as an obvious disease scenario—even at a point in time when they were experiencing a large number of virus-related surveys. At the same time, the connection between game and pandemic was not perceived as arbitrary. More than half (26 out of 51) of the participants who mentioned COVID-19 or the coronavirus by name—and some participants talking in more general terms about diseases—translated game elements back into virus-related entities or behaviors. The following are examples of such responses:

- It reminded me of the choice between gain and risk many are facing now during the pandemic. Expose yourself at work or isolate and make no money.

Table S3

Categories for associations with the transmission game in open-format asnwers.

Main category	Subcategory	n	Percent
Virus	COVID-19	51	8.5
	Virus (unspecific)	4	0.7
	General epidemiology	31	5.2
	Total	86	14.3
Economic Game	Social dilemma	19	3.2
	Other game	16	2.7
	Total	35	5.8
Game of chance	Lottery/gamble	15	2.5
	Unspecific	5	0.8
	Total	22	3.7
Novel	Not seen before	94	15.7
	Total	94	15.7
Other	Biology	4	0.7
	Finance/Business	11	1.8
	Unspecific	130	21.7
	Total	145	24.2
No comment	No comment	246	41.0
	Total	246	41.0

- This seems to replicate the dilemma currently occurring with shelter-in-place orders and the individual decision for people to either stay at home or go out. You can play it safe and help everyone else out, or you can be risky to have the better potential bonus (participation in regular activities) but your risk of turning purple or not getting a bonus is increased, and also raising the odds other people turn purple as well.
- Reminds me of "Flattening the curve" with COVID.
- This game reminds me of mathematical epidemiological models in which a virus spreads through a group of people. I would guess that the purpose of this study is to gauge to what degree people making self servicing and dangerous decisions affects the population at large.

Comparing the number of virus-mentions across conditions, there was a slightly higher number in the chain reaction condition ($n = 18$) than in other conditions ($5 < n < 10$). Other associations included economic games (social dilemmas for the most part), games of chance, or a diverse range of experienced activities, exercises or demonstrations. A substantial number of participants (15.7%) explicitly stressed the novel character of the transmission game.

S8.6 Selected Simulation Results

We simulated 1,000,000 games for each group of 100 participants, using the 2,500 decisions made by each group. Figure S60 summarizes the results for these simulated games. The pattern of these results roughly corresponds to the comparison of H-choices across conditions, with some notable distinctions. Conditions with the fewest number of H-choices resulted in distinctly lower rates of color switching and subsequently in distinctly higher expected outcomes for group members. Among conditions with high numbers of H-choices, the curves illustrate the importance of the temporal spacing of H-choices: Results for the vicarious learning condition were much better than for the descriptive norms condition. Participants in the vicarious learning condition seemed to react to the increase in purple participants by switching to G-choices later in the game, which more than compensates their slightly higher rates of H-choices in early rounds. Their curve shows some evidence of flattening and they have a higher expected outcome than participants in the control and descriptive norms condition. These outcomes already take zero-scores for purple participants into account.

To test for the robustness of our results, we repeated the full set of simulations while using a resampling method: For each trial we constructed a new group by randomly sampling participants from the original group with replacement. This resulted in a slight increase in variance, but mean results were close to indistinguishable from the original results (see Fig. S61 for a side-by-side comparison).

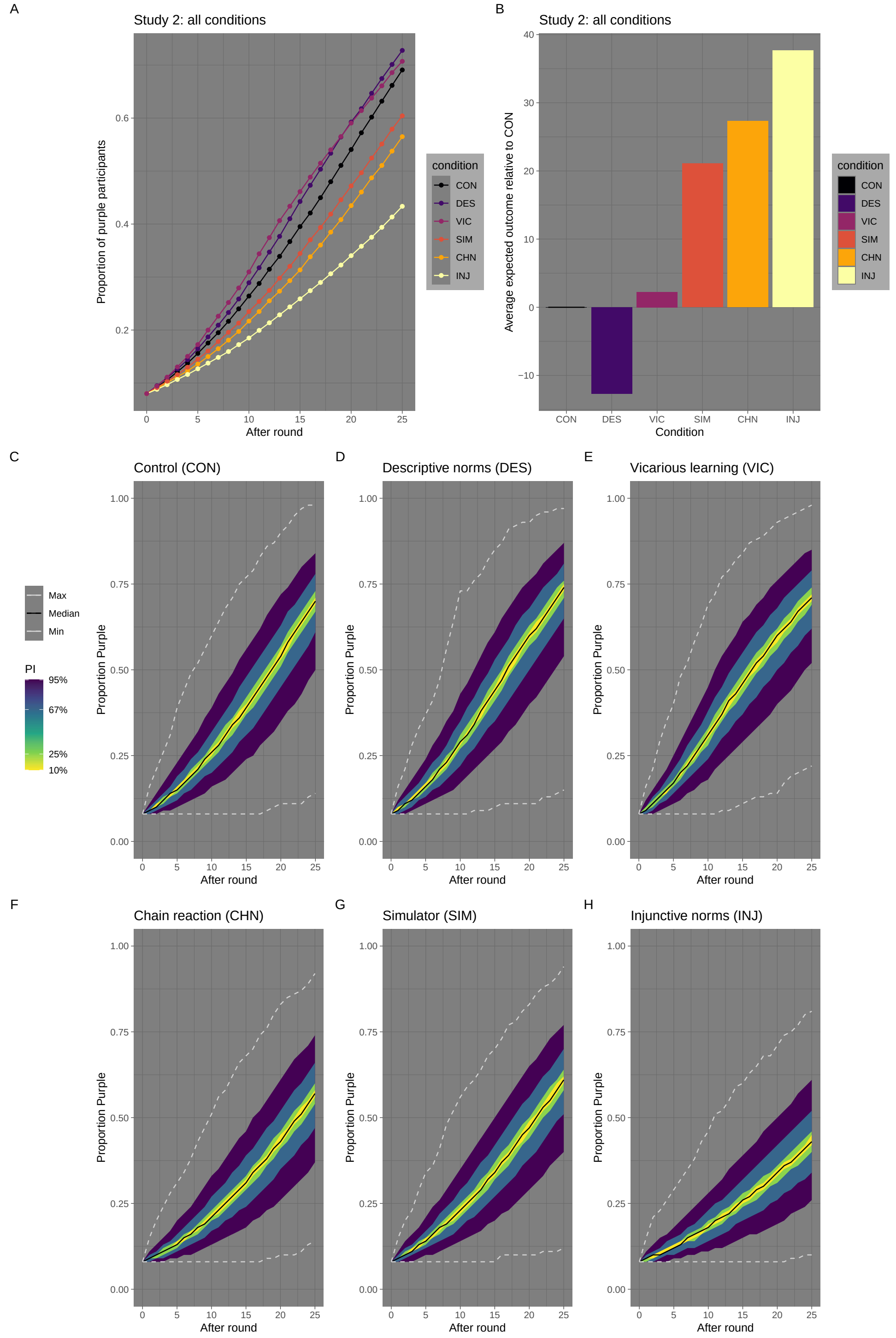

Figure S60

Results for 6,000,000 simulated games based on the observed decisions in six conditions in Study 2: Two panels show results summaries across conditions: (A) Average proportion of purple participants for each round within each condition, and (B) difference in average outcomes from the control condition. Six panels show results within conditions: Graphs show distributions of observed proportions of purple participants by round, indicating the median, minimum, maximum, and the inner 10%, 67% and 95% of results, for the (C) control (D) descriptive norms, (E) vicarious learning, (F) chain reaction, (G) simulator, and (H) injunctive norms condition.

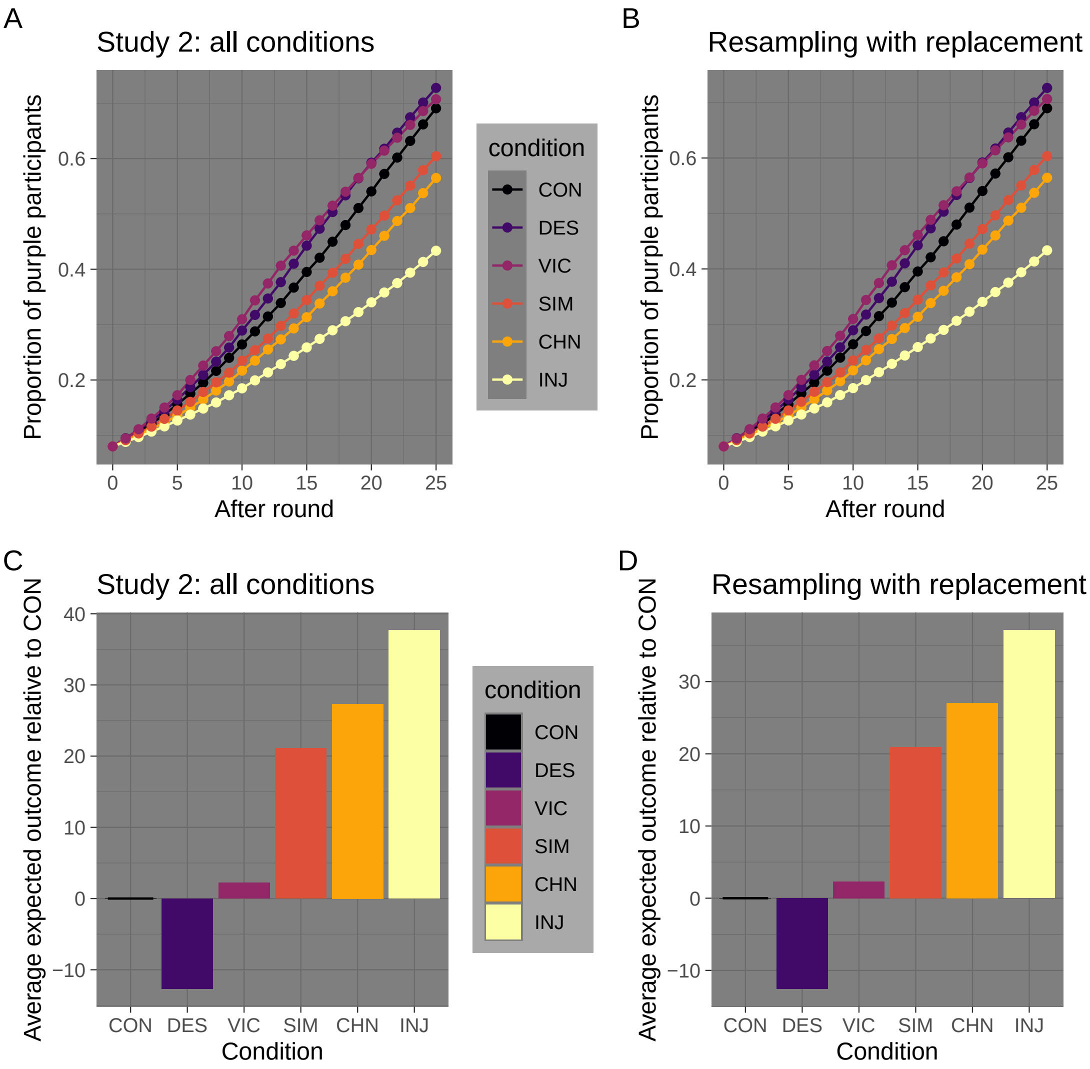

Figure S61

Comparison of original simulation results and simulation results after resampling: Upper panels show round-wise average proportions of purple participants for each of the six conditions (A) for the original, and (B) for the resampled groups. Lower panels show average outcomes for each of the six conditions (C) for the original, and (D) for the resampled groups.

Finally, Figure S62 demonstrates that the basic social dilemma structure of the game is reflected in outcomes for each of the six conditions. Here, results are shown split by strategy used in each of the six conditions. We still observed higher rates of receiving a bonus for players using the AllG strategy (see **SCR** for additional results, `https://doi.org/10.7910/DVN/ILDEMO`), but given the large score differences, AllH players had a higher expected outcome than AllG players in each of the six conditions, with mixed strategies falling in between in most conditions. HG-players on average outperformed Switch-players.

Replicating the observations for theoretical populations, the difference in payoffs for competitive and cooperative strategies was higher in conditions with a larger proportion of cooperative choices. Free-riders thus profit from other participants' efforts to reduce the amount of color-switching. It is important to consider these results jointly with the difference in payoff rates. While risk-taking players can expect higher average outcomes, they are faced with a lower probability of receiving a bonus at all. The observed correlation between general risk-taking and competitive choices might indicate that participants were at least to some degree aware of this tradeoff. The observed correlation with social preferences might also indicate that participants were at least to some degree aware of the social dilemma structure of the game.

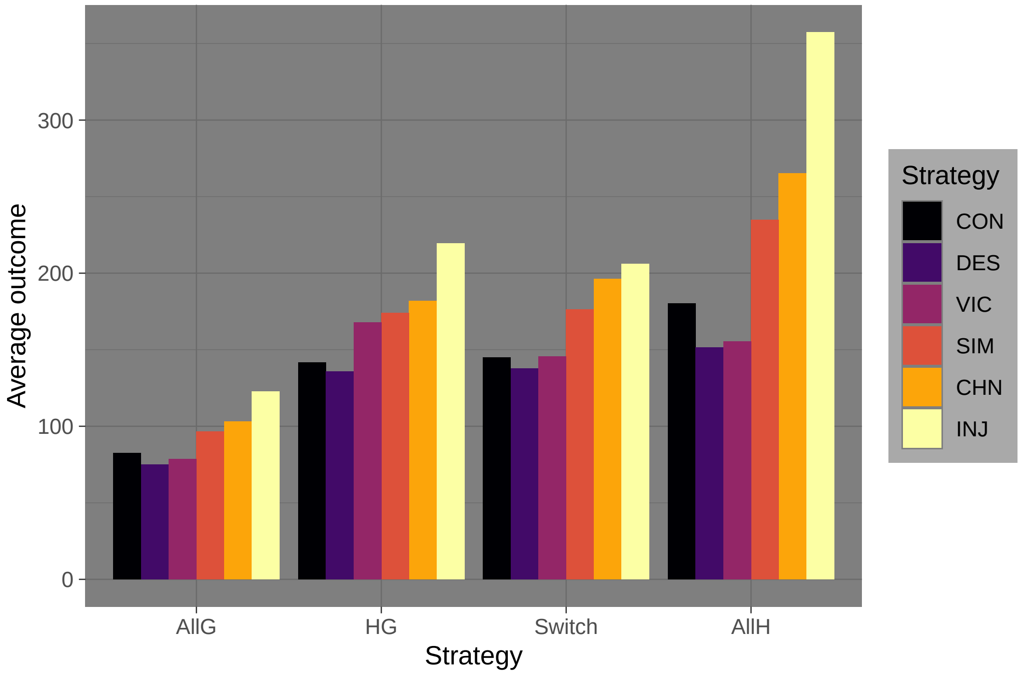

Figure S62

Average outcomes across simulated games for participants categorized as using each of the four possible strategies for each of the six conditions.
